# Evolution in the Model Genus *Antirrhinum* Based on Phylogenomics of Topotypic Material

**DOI:** 10.3389/fpls.2021.631178

**Published:** 2021-02-12

**Authors:** Ana Otero, Mario Fernández-Mazuecos, Pablo Vargas

**Affiliations:** Real Jardín Botánico (RJB-CSIC), Madrid, Spain

**Keywords:** biogeography, genotyping-by-sequencing, high-throughput sequencing, *locus classicus*, molecular dating, phylogeny, Plantaginaceae, systematics

## Abstract

Researchers in phylogenetic systematics typically choose a few individual representatives of every species for sequencing based on convenience (neighboring populations, herbarium specimens, samples provided by experts, garden plants). However, few studies are based on original material, type material or topotypic material (living specimens from the locality where the type material was collected). The use of type or topotypic material in phylogenetic studies is paramount particularly when taxonomy is complex, such as that of *Antirrhinum* (Plantaginaceae). In this paper, we used topotypic materials of *Antirrhinum* at the species level (34 species proposed by previous authors), 87 specimens representing the species distributions and >50,000 informative nucleotide characters (from ∼4,000 loci) generated by the genotyping-by-sequencing (GBS) technique: (i) to test two explicit taxonomic hypotheses widely followed by local taxonomic treatments; (ii) to robustly estimate phylogenetic relationships; (iii) to investigate the evolution of key morphological characters and biogeographic centers of differentiation. Two GBS phylogenies based on two datasets (87 localities and 34 topotypic specimens) revealed that: (1) Sutton’s (1988) taxonomic account is the most congruent with phylogenetic results, whereas division of *Antirrhinum* into three major clades disagrees with Rothmaler’s (1956) infrageneric classification; (2) monophyly of populations currently included in the same species is primarily supported; (3) the historically recognized *Antirrhinum majus* group is not monophyletic; (4) sister-group relationships are robust for eight species pairs; (5) the evolutionary radiation of 26 species since the Pliocene is underpinned given a high rate of diversification (0.54 spp. Myr^–1^); (6) a geographic pattern of speciation is reconstructed, with northern Iberia as the center of early diversification followed by more recent speciation in southeastern Iberia; and (7) multiple acquisitions of key taxonomic characters in the course of *Antirrhinum* diversification are strongly supported, with no evidence of hybridization between major clades. Our results also suggest incipient speciation in some geographic areas and point to future avenues of research in evolution and systematics of *Antirrhinum*.

## Introduction

In botanical systematics and taxonomy, validly published species names are based on type materials, which are deposited in reference collections (herbaria). This way, any species is anchored to a single name and type material, i.e., a single specimen designated by taxonomists following the rules of the *International Code of Nomenclature for Algae, Fungi, and Plants*^1^. As a result, researchers can name any individuals or populations based on key characters contained in the type specimen, with the assistance of any other original materials.

Plant taxonomists have historically found two significant patterns: (1) not all plant groups have clear-cut characters circumscribing populations into species; and (2) unrelated species share similar morphological characters that have been independently acquired in the course of evolution (convergence and parallel evolution). The advent of molecular phylogenetics helped to tackle these two taxonomic obstacles. Taxonomy takes advantage of DNA sequence data not only for improving species circumscription, but also for species-level identification with the development of DNA barcoding (Li et al., 2015). In contemporary taxonomic studies, DNA sequences are frequently included to validate new species, whereas many historical specimens do not have sequences associated. These historical specimens include original material (the very specimens used for the first description of a species) and type material (physical specimen that serves as exemplar for any formal species name, preferentially selected from among the original material). However, sequencing of original and type materials usually faces two problems: (i) tissue destruction during DNA extraction, which may cause permanent damage to unique specimens (herbarium policies); and (ii) high DNA fragmentation because of specimen age, which hinders sequencing (Staats et al., 2011). Consequently, the utility of old herbarium specimens (including original and type materials) in phylogenetic studies is limited. To circumvent these two problems, an alternative approach relies on topotypic specimens, i.e., those collected at the type locality of a species, which usually corresponds to the *locus classicus* (the locality where plants were originally collected and studied for description of a new species). Topotypic specimens are expected to retain the genetic identity of the type material better than plants collected in any other locality. Unfortunately, researchers do not commonly use topotypic specimens, but rather identify species of a natural group (e.g., a genus) using the most recent taxonomic studies and then choose a few individual representatives of each species for sequencing based on convenience (neighboring populations, herbarium specimens, samples provided by experts, and garden individuals). Most phylogenetic reconstructions unfortunately ignore that any species name is ultimately linked to the type material, which is particularly critical when taxonomy is complex. In other words, to which degree can we rely on species-level phylogenetic relationships based on sequenced specimens whose genetic makeup may differ significantly from that of the type materials?

Taxonomy is particularly complex for plant groups actively evolving in biodiversity hotspots such as the Mediterranean region (Vargas et al., 2018). One of these groups is the model genus *Antirrhinum* L. (snapdragons), of which around 40 species have been historically proposed to circumscribe morphological differentiation of populations primarily distributed throughout the western Mediterranean. Published studies have repeatedly failed to obtain a well-supported phylogenetic structure using Sanger sequencing of nuclear and plastid DNA regions (Vargas et al., 2004, 2009; Carrió et al., 2010). A more resolved phylogeny was obtained by analyzing AFLP data, but species delimitation was still largely unresolved (Wilson and Hudson, 2011). A phylogenomic analysis based on genome-wide data is, however, promising to disclose evolutionary relationships among populations and species.

In this study, we investigated the phylogenetic structure of snapdragons (*Antirrhinum* species) by analyzing genome-wide genotyping-by-sequencing (GBS) data obtained from topotypic material of most species (36 specimens). In addition, materials of widespread species were collected from distant localities covering species distributions to test the monophyly of currently recognized taxa of *Antirrhinum*. Therefore, the main aim was to obtain a solid species-level phylogeny using specimens that are strongly linked to valid names. To this end, we tested two explicit taxonomic hypotheses proposed by the two worldwide taxonomic accounts of *Antirrhinum* published to date (Rothmaler, 1956; Sutton, 1988). We expected that species relationships and clade composition would help to propose a more consistent taxonomic and evolutionary framework for *Antirrhinum* at the species and supra-specific levels. Specific objectives were: (1) to analyze phylogenetic relationships of the populations and species described by previous authors; (2) to reconstruct primary and secondary centers of species diversification in a spatio-temporal framework; (3) to investigate the evolution of key morphological characters; and (4) to test the radiation hypothesis (abundant speciation from a common ancestor in a short period of time) proposed by previous studies.

## Materials and Methods

### Study System

**FIGURE 1:**
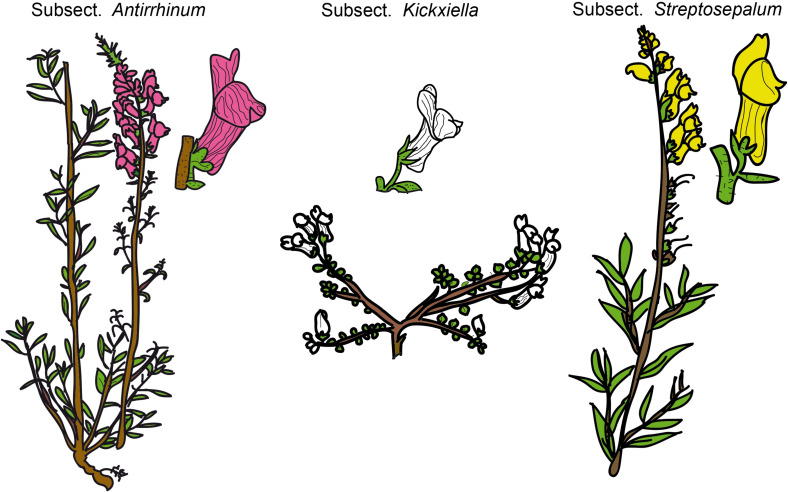
Schematic drawings of the three morphotypes of *Antirrhinum* based on the Rothmaler (1956) subsections. Drawings have been adapted from Güemes (2009).

**FIGURE 2:**
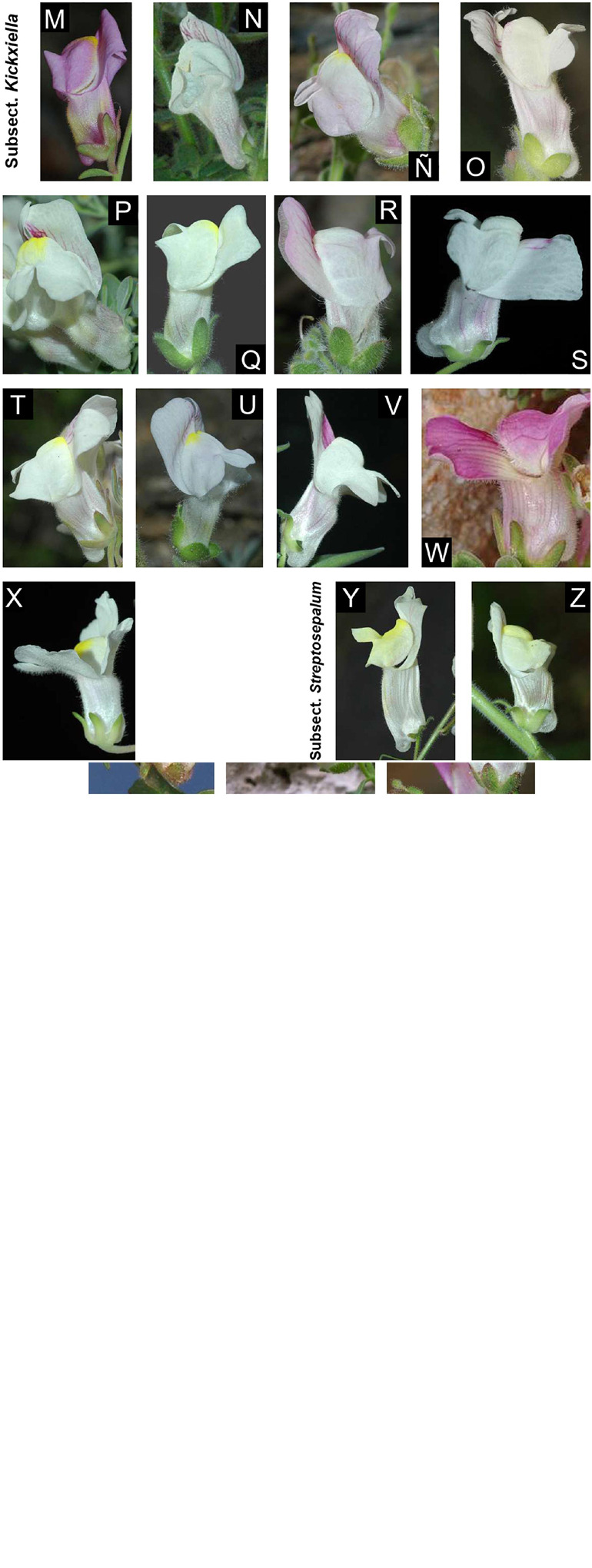
Diversity of flower color and shape in the 26 recognized species in this study. The photographs are arranged by subsections: subsect. *Antirrhinum* **(A–L)**; subsect *Kickxella* **(M–X)**; and subsect. *Streptosepalum* (Y-Z). Photographs: **(A)** *A. australe*; **(B)** *A. barrelieri*; **(C)** *A. cirrhigerum*; **(D)** *A. controversum*; **(E)** *A. graniticum* subsp. *graniticum*; **(F)** *A. latifolium*; **(G)** *A. linkianum*; **(H)** *A. majus* subsp. *majus*; **(I)** *A. majus* subsp. *striatum*; **(J)** *A. onubensis*; **(K)** *A. siculum*; **(L)** *A. tortuosum*; **(M)** *A. charidemi*; **(N)** *A. grosii*; **(Ñ)** *A. hispanicum*; **(O)** *A. lopesianum*; **(P)** *A. microphyllum*; **(Q)** *A. molle*; **(R)** *A. mollissimum*; **(S)** *A. pertegasii*; **(T)** *A. pulverulentum*; **(U)** *A. rupestre*; **(V)** *A. sempervirens*; **(W)** *A. subbaeticum*; **(X)** *A. valentinum*; **(Y)** *A. braun-blanquetii*; and **(Z)** *A. meonanthum*. All photographs were taken in Spain by Pablo Vargas, except for **(F)** van der Strate, Saxifraga Foundation; **(G)** (Luis Nunes; Wikipedia); **(K)** (Denis Barthel; Wikipedia), and **(W)** (José Quiles; www.florasilvestre.es). Locations for each photograph is detailed on Supplementary Material (see Supplementary Data Sheet 1).

Snapdragons (species of *Antirrhinum*) have been considered a model system for the study of plant genetics, development, and evolution since the 20th century because of their easy cultivation and morphological diversity (Schwarz-Sommer et al., 2003; Fernández-Mazuecos and Glover, 2017). The complexity of *Antirrhinum* systematics has historically been ascribed to the recent diversification of the genus, putatively accompanied by hybridization and introgression. In fact, dissimilar taxonomic treatments exist, of which those of Rothmaler (1956) and Sutton (1988) are the most complete to date. Rothmaler (1956) divided *Antirrhinum* into two sections: sect. *Saerorrhinum* and sect. *Antirrhinum*. Sect. *Saerorrhinum* comprised North American plants that are currently included in the genus *Sairocarpus* and other related genera, while sect. *Antirrhinum* comprised all the species currently included in the genus *Antirrhinum*. Rothmaler (1956) also split sect. *Antirrhinum* into three subsections: subsect. *Antirrhinum*, subsect. *Kickxiella* and subsect. *Streptosepalum*. These three subsections represent three major morphotypes/ecotypes: (1) species in subsect. *Antirrhinum* grow in sandy soils and have an upright tall habit, long thin leaves with no hairs and magenta or yellow flowers; (2) species in subsect. *Kickxiella* inhabit rocks and are small, prostrate, xerophytic woody plants; and (3) species in subsect. *Streptosepalum* occur on rocky substrates and have long thin leaves, glandular hairs only on the inflorescence and large yellow flowers (Figures 1, 2; Wilson and Hudson, 2011). The Iberian Peninsula appears to be the center of diversification of snapdragons (Vargas et al., 2014), although the distribution range of *Antirrhinum* encompasses most of Mediterranean Europe, northern Africa and the Middle East (Sutton, 1988).

### Specimen Sampling

The sample was designed to obtain the phylogenetic structure of *Antirrhinum* based on the majority of the taxa validly described at the species level. A total of 32 of the 34 species included in the taxonomic treatments of Rothmaler (1956) and Sutton (1988) were sampled. Three more species recognized by specialists after Sutton’s (1988) account, were sampled: *A. onubensis* (Fernández-Casas, 1987), *A. subbaeticum* (Güemes et al., 1993), and *A. rothmaleri* (García-Barriuso et al., 2011) (see Güemes, 2009; Table 1 and Supplementary Table 1, in Supporting Information). The only two species in Rothmaler (1956) and Sutton (1988) of which no material was found were *A. ambiguum* Lange and *A. martenii* (Font Quer) Rothm. In the *locus classicus* of the former species (El Escorial, Madrid) we collected some snapdragons, but they had morphological characters more similar to those of *A. graniticum* than to the description (protologue) of *A. ambiguum*. Unfortunately, *A. martenii* from Morocco has not been found since description (1956), including unsuccessful collecting campaigns in recent times (E. Carrió pers. com.).

**TABLE 1:** Species considered for this study, including species names from the two worldwide accounts of *Antirrhinum* (Rothmaler, 1956; Sutton, 1988) and new species described after Sutton (1988).

Species considered in this study	Species considered in Rothmaler (1956)	Species considered in Sutton (1988)	Valid names after Sutton (1988)	*Locus classicus*	Sampled Material from *locus classicus*: Voucher number	Distance in km from sampled material to *Locus classicus*)	Geographic distribution (geographic range size: I, II or III)	Total number of specimens sampled	Main phylogenetic clade (yes/no species monophyly)	Ecology
*A. ambiguum* Lange	*A. ambiguum* Lange (2)	–	–	Castella Nova supra Escorial, Cerro Las Machotas, Madrid (Spain)	Not available	Not available	Sierra de Guadarrama, Serra da Estrela (I)	Not available	Not available	Rocky substrates
*A. australe* Rothm.	*A. australe* Rothm. (3)	*A. australe* Rothm.	–	Benacoaz, Cádiz (Spain)	3IML12(1) (2)	*	SE Spain (II)	4	III (yes)	Sandy soils
*A. barrelieri* Boreau	*A. majus* subsp. *litigiosum* (Pau) Rothm. (3)	*A. majus* subsp. *litigiosum* (Pau) Rothm.	–	Calatayud, Zaragoza (Spain)	141PV08(4)	Nuévalos (20.37 km)	NE Spain (II)	5	III (yes)	Sandy soils
*A. boissieri* Rothm.	*A. boissieri* Rothm. (3)	*A. hispanicum* Chav.	–	Granada, Silla del Moro (Spain)	6IML13(1) (2)	*	SE Spain (I)	3	III (yes)	Sandy soils
*A. braun-blanquetii* Rothm.	*A. braun-blanquetii* Rothm. (2)	*A. braun-blanquetii* Rothm.	–	La Guiana, Dolomitklippen de Apóstoles, León (Spain)	MA777373/MA345884	*	N Iberian Peninsula (II)	6	II (yes)	Rocky substrates
*A. caroli-paui* Rothm.	*A. caroli-paui* Rothm. (1)	*A. molle* var. m*arianum* Pau	–	Doña María de Ocaña, Almería (Spain)	53PV07	Abrucena (10 Km)	SE Spain (I)	2	III (–)	Rocky substrates
*A. charidemi* Lange	*A. charidemi* Lange (1)	*A. charidemi* Lange	–	Promontorio Charidemi, Cabo de Gata, Almería (Spain)	PV05	*	SE Spain (I)	2	III (–)	Rocky substrates
*A. cirrhigerum* (Welv. ex Ficalho) Rothm.	*A. linkianum* var. *ramosissimum* Willk	*A. majus* subsp. *cirrhigerum* (Welv. ex Ficalho) Franco	–	Int. Sines et Milfontes (Portugal)	3AO19(7)	*	SW Iberian Peninsula, NW Norocco (II)	3	III (–)	Sandy soils
*A. controversum* Pau	*A. barrelieri* Boreau (3)	–	–	Sierra de Cártama, Málaga (Spain)	MA855349	Pizarra (9 Km)	S Spain (I)	6	III (yes)	Sandy soils
*A. dielsanum* Rothm.	*A. dielsanum* Rothm. (3)	–	–	Siracusa, Sicilly (Italy)	MA646409	*	Sicilly (Italy) (I)	1	III (–)	Sandy soils
*A. graniticum* Rothm. subsp. *graniticum*	*A. graniticum* Rothm. (3)	*A. graniticum* Rothm.	–	Castelo Branco, Lardosa prope. Soalheira (Portugal)	1AO19(2)/ 2AO19(2)	*	E Portugal to C Spain (III)	6	III (yes)	Sandy soils
*A. graniticum* Rothm. subsp. *brachycalyx* D. A. Sutton	–	*A. graniticum* Rothm. subsp. *brachycalyx* D. A. Sutton	–	Madrid, near Valdemoro (Spain)	104PV19(14)	*	Madrid, Toledo (Spain) (I)	2	III (–)	Sandy soils
*A. grosii* Font Quer	*A. grosii* Font Quer (1)	*A. grosii* Font Quer	–	Sierra de Gredos, Riscos del Morezón, Ávila (Spain)	133PV10(1)/ 138PV10(7)	*	Sierra de Gredos, Ávila (Spain) (I)	2	II (yes)	Rocky substrates
*A. hispanicum* Chav.	*A. hispanicum* Chav. (1)	*A. hispanicum* Chav.	–	Hispania Tournefort s.n (holo. P-Tournefort, fragment JE!	19IML12(2)/ 7IML13(3)	*	SE Spain (II)	2	III (no)	Rocky substrates
*A. latifolium* Mill.	*A. majus subsp. latifolium* (Mill.) Rouy (3)	*A. latifolium* Mill.	–	In ins Archipielagi Toscana (Italy)	PI009621	*	Slovenia and C Italy (III)	4	III (yes)	Sandy soils
*A. linkianum* Boiss. & Reut.	*A. majus* subsp. *linkianum* (Boiss. & Reut.) Rothm. (3)	*A. majus* subsp. *linkianum* (Boiss. & Reut.) Rothm.	–	Ad sepes Olyssoponi (Sintra, Lisbon, Portugal)	3MF13(2) (5)	*	NW and CW Portugal (II)	3	III (yes)	Sandy soils
*A. lopesianum* Rothm.	*A. lopesianum* Rothm. (1)	*A. lopesianum* Rothm.	–	Vimioso prope Argoselo (Portugal)	MA824747	*	NE Portugal, NW Spain (I)	4	II (yes)	Rocky substrates
*A. majus* L.	*A. majus* subsp. *majus* (3)	*A. majus* L.	–	Eur. Austr. (Linné).	46PV12(1)	*	SW Europe and Mediterranean region (widely planted as ornamental) (II)	5	III (no)	Sandy soils
*A. martenii* (Font Quer) Rothm.	*A. martenii* (Font Quer) Rothm.	*A. martenii* (Font Quer) Rothm.	–	Rupibus Bar-er-Ruida, (Morocco)	Not available	Not available	N Morocco (I)	Not available	Not available	Rocky substrates
*A. meonanthum* Hoffmanns. & Link	*A. meonanthum* Hoffmanns. & Link (2)	*A. meonanthum* Hoffmanns. & Link	–	Lusitania, ad ripas Durii (Douro River near Oporto)	PO60051	Cinfães (35 Km)	N Portugal, W and C Spain (II)	5	II (no)	Rocky substrates
*A. microphyllum* Rothm.	*A. microphyllum* Rothm. (1)	*A. microphyllum* Rothm.	–	Sacedón, Guadalajara (Spain)	39PV08(2)/ 40PV08(2)	*	NE Spain (I)	2	I (yes)	Rocky substrates
*A. molle* L.	*A. molle* L. (1)	*A. molle* L.	–	Pyrenees (Spain, France)	MA895768/MA756423	*	NE Spain, Pyrenees and adjoining mountains (II)	3	III (yes)	Rocky substrates
*A. mollissimum* (Pau) Rothm.	*A. mollissimum* (Pau) Rothm. (1)	*A. mollissimum* (Pau) Rothm.	–	Barranco del Caballar, Almería (Spain)	73PV06	*	SE Spain (I)	3	III (yes)	Rocky substrates
*A. onubensis* (Fern. Casas) Fern. Casas	–	–	*A. onubensis* (Fern. Casas) Fern. Casas (Fernández-Casas, 1987)	Sierra Aracena, Huelva (Spain)	104PV09/146PJM13(3)	*	Sierra Aracena, Huelva (Spain) (I)	2	III (yes)	Rocky substrates
*A. pertegasii* Pau ex Rothm.	*A. pertegasii* Pau ex Rothm. (1)	*A. pertegasii* Pau ex Rothm.	–	Mont Caro, Montes de Tortosa, Tarragona (Spain)	36PV11/38PV11(12)	La Senia (18.81 Km)/ La Pobla de Benifassa (22.86 km)	E Spain (I)	2	I (yes)	Rocky substrates
*A. pulverulentum* Lázaro Ibiza	*A. pulverulentum* Lázaro Ibiza (1)	*A. pulverulentum* Lázaro Ibiza	–	Monasterio de Piedra, Zaragoza (Spain)	15IML12(4)/ (5)	*	E Spain (II)	3	I (no)	Rocky substrates
*A. rothmalieri* (Pinto da Silva) Amich, Bernardos & García-Barriuso	–	–	*A. rothmalieri* (Pinto da Silva) Amich, Bernardos & García-Barriuso (García-Barriuso et al., 2011)	Macedos de Cavaleiros (Portugal)	12IML12(4) (2)	*	NW Iberian Peninsula (I)	2	II (–)	Sandy soils
*A. rupestre* Boiss. & Reut	*A. rupestre* Boiss. & Reut (1)	*A. hispanicum* Chav.	*–*	Sierra Nevada, Granada (Spain)	9IML13(3) (5)	*	SE Iberian Peninsula (I)	2	III (–)	Rocky substrates
*A. sempervirens* Lapeyr.	*A. sempervirens* Lapeyr. (1)	*A. sempervirens* Lapeyr.	–	Hautes Pyreneés, Gèdre (France)	104PV10(7)	Panticosa (Spain) (25.77 Km)	Pyrenees (Spain, France) (II)	4	I (–)	Rocky substrates
*A. siculum* Mill.	*A. siculum* Mill. (3)	*A. siculum* Mill.	–	Palermo, Sicily (Italy)	MA705546/33bisPV2015(1)	*	S Italy and Sicily (III)	4	III (yes)	Sandy soils
*A. striatum* Lam.	*A. majus* subsp. *striatum* (DC) Rothm. (3)	*A. latifolium* subsp. *intermedium* (Debeaux) Nyman	*A. latifolium* var. striatum DC (Güemes, 2009)	Perpignan (France)	55PV07(1)	Limoes (France) (66.54 km)	SE France and NE Spain (I)	2	III (–)	Sandy soils
*A. subbaeticum* Güemes, Mateu & Sánchez Gómez	–	–	*A. subbaeticum* Güemes, Mateu & Sánchez Gómez (Güemes et al., 1993)	Bogarra, Albacete (Spain)	MA705104/MA593205	*	SE Spain (I)	2	I (yes)	Rocky substrates
*A. tortuosum* Bosc ex Lam.	*A. majus* subsp. *tortuosum* (Bosc ex Vent.) Rouy (3)	*A. majus* subsp. *tortuosum* (Bosc ex Vent.) Rouy	–	Described from material cultivated at Paris of Italian origin	MA589750/PI010588	*	Mediterranean Region (III)	7	III (yes)	Sandy soils
*A. valentinum* Font Quer	*A. valentinum* Font Quer (1)	*A. valentinum* Font Quer	–	Mt Mondúber, supra Gandía, Valencia (Spain)	27PV11(6)/ 32PV11(3)	*	SE Spain (I)	2	I (yes)	Rocky substrates

*The locus classicus for each species is transcribed from each original protologue. Numbers in brackets in the column for Rothmaler (1956) names indicate species subsections: (1) subsect. Kickxiella; (2) subsect. Streptosepalum; and (3) subsect. Antirrhinum. Voucher numbers and indication of locus classicus (asterisk) for each species or distance (in km) where samples were taken from the locus classicus are indicated in two more columns. Species distribution ranges categorized in three types are shown (I: restricted; II: moderate; III: wide), which help to define sampling intensity for each species (see Species sampling). Information about phylogenetic clade to which each species belongs and whether monophyly is retrieved [or dash line (–) when monophyly cannot be assessed] according to our phylogenetic results is also indicated. Plant collectors (AO, Ana Otero; IML, Isabel María Liberal; MF, Mario Fernández-Mazuecos; PV, Pablo Vargas; PJM, Pedro Jiménez Mejías) and herbaria (MA, Royal Botanical Garden of Madrid; PI, Pisa; PO, Porto Herbarium) that provided plant material. For details about Antirrhinum see www.rjb.csic.es/snapdragons/.*

The type species of the genus (*A. majus*) was described from cultivated specimens (Linnaeus, 1753), and thus it is not possible to localize its original source with certainty. Sutton (1988) indicates that the most characteristic morphological features of *A. majus* are found in wild populations of southern France and north-eastern Spain, where we sampled three specimens (see Supplementary Table 1). For the remaining species, a minimum of two topotypic specimens per species were sampled. When we failed to sample at the *locus classicus* itself, plants from nearby locations were collected. To test monophyly of each species, additional populations (from two to five depending on species range size) were sampled from distant locations. Based on distribution ranges reviewed by Vargas et al. (2009), three range size categories were considered: (I) widely distributed species (four to six samples); (II) moderately distributed species (three to five samples); and (III) narrowly distributed species (two to three samples) (see Table 1). As a result, 108 samples of *Antirrhinum* were initially included as the ingroup. Ten samples of genera belonging to the sister clade to *Antirrhinum* (Fernández-Mazuecos et al., 2019) were sampled as the outgroup (Supplementary Table 1). Most materials were collected in multiple field campaigns (2005–2019) or obtained from herbarium specimens (JACA, LEB, MA, MGC, PI, PO, and SALA).

### DNA Extraction and GBS Library Preparation

DNA was extracted from leaf tissue (c. 20 mg) using a modified CTAB protocol (Doyle and Doyle, 1987; Cullings, 1992) and extractions were quantified at the Next Generation Sequencing Lab (Real Jardín Botánico, CSIC, Madrid, Spain) using a Qubit Fluorometer (Thermo Fisher, Waltham, MA, United States). The 118 genomic DNA samples (ingroup plus outgroup samples, 500 ng of DNA per sample when available) were sorted into two 96-well plates, and four samples per plate were replicated in order to evaluate possible biases derived from the lab technique, thus raising the total number of samples to 126. These were used to prepare two separate GBS libraries using the *Pst*I-HF restriction enzyme and following the previously published procedures of Fernández-Mazuecos et al. (2018), which were adapted from Elshire et al. (2011), Escudero et al. (2014), and Grabowski et al. (2014) with some modifications. The 500 ng of genomic DNA per sample were combined with 0.6 pmol of a sample specific barcode adapter and 0.6 pmol of common adapter. Four units of *Pst*I-HF (NEB, MA, United States) were used for the digestion step at 37°C overnight. The ligation step was done using 400 units of T4 DNA Ligase (NEB, MA, United States) at room temperature for 4 h. Then, 50 ng of each sample were pooled and purified with Agencourt AMPure XP beads (Beckman Coulter, CA, United States). DNA pools of each library were amplified for 19 PCR cycles using NEB 2x Taq MasterMix (NEB, MA, United States). The PCR products were purified using different ratios of AMPure beads. Concentration and fragment sizes were assessed in a 2100 Bioanalyzer (Agilent Technologies, CA, United States) at the genomic facilities of Real Jardín Botánico (CSIC, Madrid, Spain). The optimal purification ratio of AMPure beads (1:1) yielded a concentration of 2.13 and 2.63 ng/μl, with an averaged fragment size of 498.5 and 561 bp for each library, respectively. Both libraries were submitted to Macrogen Inc. (Seoul, South Korea) for 150 bp HiSeqX Illumina paired-end sequencing. Quality control of sequencing results was conducted in FastQC 0.11.7 (Andrews, 2010).

### Data Assembly

The FASTQ sequence files from the two libraries were processed in ipyrad v.0.9.4 (Eaton and Overcast, 2020). Ipyrad is an assembly pipeline implementing seven sequential steps that have been specifically developed for the processing of high-throughput sequencing results derived from restriction digest-based methods such as GBS. We first demultiplexed and filtered reads from both libraries separately (steps one and two) using the default threshold of 33 quality score for filtering of reads, and then combined all samples from both libraries for subsequent assembly steps. Data were assembled using the reference mapping method, incorporating BWA and Bedtools algorithms (Li and Durbin, 2009; Quinlan and Hall, 2010), in which GBS reads are mapped to a reference genome to determine homology and all sequences that do not match the reference genome are discarded. As reference, we used the genome of the cultivar line JI7 of *A. majus* published by Li et al. (2019). Samples with low-quality results (<100 sequenced loci in preliminary assemblies) were discarded, leading to a total of 87 samples in the final datasets (trimmed dataset). Additional datasets were produced including topotypic specimens only (topotypic dataset, 38 samples; see Table 2). To assess the sensitivity of the results to data matrix completeness and number of loci, we tested four values of minimum taxon coverage, i.e., the minimum number of sequenced samples for a locus to be considered in the final matrix (m4, m6, m18, and m36). Thus, a total of eight assembled datasets were produced (see Table 2).

**TABLE 2:** Parameter combination of nucleotides assembled GBS datasets generated in ipyrad and used in the phylogenetic analyses.

Data set	Minimum taxon coverage	Number of loci	Concatenated length (bp)	Missing data (%)	Number of parsimony-informative sites
**Trimmed dataset: 87 taxa**					
refm4	4	6524	1696692	78.8	52991
refm6	6	4663	1276470	73	51140
refm18	18	2707	807146	61.7	42203
refm36	36	1989	626043	56.8	35381
**Topotypic dataset: 38 taxa**					
ref_clas_m4	4	5240	1262819	64	39093
ref_clas_m6	6	4016	1008597	57.3	37713
ref_clas_m18	18	2344	632938	43.8	30014
ref_clas_m36	36	237	78284	32.6	3969

### Phylogenomic Analyses

Maximum likelihood (hereafter ML) analyses of the eight matrices of concatenated loci were performed through RAxML BlackBox (Stamatakis et al., 2008) by using the GTRCAT model of nucleotide substitution and automatic bootstrap stopping as recommended in CIPRES Portal (Miller et al., 2010). Among the four topologies obtained for each dataset (trimmed and topotypic dataset), we chose the one with the highest average bootstrap support (BS) for downstream phylogeny-based analyses. The matrices producing the best RAxML trees were also analyzed using Bayesian inference in ExaBayes 1.5 (Aberer et al., 2014), implemented in the CIPRES. We specified two runs, four coupled chains, one million generations with a sampling frequency of 500 and a burn-in proportion of 0.1. Convergence and effective sample size (ESS) values for all parameters were assessed using Tracer v.1.6 (Rambaut et al., 2014).

We additionally implemented the coalescent-based method SVDquartets (Chifman and Kubatko, 2014) in PAUP (Swofford, 2001). Individuals were grouped according to current species circumscriptions. All possible quartets were evaluated with 100 bootstrap replicates.

### Estimates of Divergence Times

We used penalized likelihood as implemented in TreePL (Smith and O’Meara, 2012) to estimate a time-calibrated tree for the topotypic dataset using the best RAxML tree. TreePL is suitable for divergence time estimation when dealing with large amounts of data, such as those yielded by GBS (Zheng and Wiens, 2015). The tree was pruned to include a single topotypic individual per species of *Antirrhinum* by choosing the individual with the highest number of loci retrieved from the assembly. Two calibration points were used: (i) root age (minimum age = 11.0583; maximum age = 21.58182) and (ii) crown node of *Antirrhinum* (ingroup) (minimum age = 2.1168; maximum age = 5.6736) based on the 95% Highest Posterior Density (HPD) intervals inferred in Fernández-Mazuecos et al. (2019) and Gorospe et al. (2020). We first conducted an analysis under the “prime” option to select the optimal set of parameter values. Then we set the gradient-based (opt) optimizer to one, and autodifferentiation-based (optad) and autodifferentiation crossvalidation-based optimizers (optcvad) to two. Then, we ran a second analysis using random subsample and replicate crossvalidation (RSRCV) to identify the best value for the smoothing parameter. We ran the final analysis by setting the best chi-square value for the smoothing parameter (smoothing = 1e-129). For all runs, we used the thorough analysis option and set 200,000 iterations for penalized likelihood and 5,000 iterations for cross-validation. Based on estimated crown age, net diversification rate was calculated following the whole-clade method of Magallón and Sanderson (2001), implemented in the R package Geiger (Harmon et al., 2007).

### Biogeographic Reconstruction

For biogeographic analyses we used the ultrametric tree resulting from TreePL after pruning the outgroup to avoid anomalous inferences of ancestral areas that may have resulted from the difference in sampling depth between outgroup and ingroup lineages. This approach also circumvents the potential effect of extinction between the outgroup and the ingroup expected after millions of years since the Oligocene (Gorospe et al., 2020). We additionally excluded four taxa originally described as different species but with limited phylogenetic and morphological distinctiveness that may have produced biogeographic bias: *A. rothmalieri*, *A. dielsianum, A. caroli-paui*, and *A. boissieri*. Areas for biogeographic reconstruction were based on the set of areas of endemicity for *Antirrhinum* proposed by Vargas et al. (2009). As a result, we considered six areas: (1) northwest of the Iberian Peninsula; (2) northeast of the Iberian Peninsula; (3) southwest of the Iberian Peninsula; (4) southeast of the Iberian Peninsula; (5) northern Africa; and (6) remaining circun-Mediterranean areas (non-Iberian Europe and SW Asia). We tested dispersal-extinction-cladogenesis (DEC, Ree and Smith, 2008) and dispersal-vicariance (DIVA, Ronquist, 1997) models using the “BioGeoBEARS” package (Matzke, 2013) in R (R Core Team, 2013) with no constraints in dispersal rates and adjacency between areas. In particular, the “BioGeoBEARS” package implements a likelihood interpretation of the parsimony-based DIVA method (DIVALIKE), i.e., processes are modeled as in DEC modeling, but including only the parameters considered in DIVA (widespread vicariance but not subset sympatry; Ronquist and Sanmartín, 2011). AIC was calculated for the two models although, given the inadequacy of likelihood comparison between different biogeographic models, DEC and DIVA results were discussed based on biological criteria (see Ree and Sanmartín, 2018).

### Ancestral State Reconstruction

To evaluate the evolution of taxonomic characters traditionally used for *Antirrhinum* species delimitation, we selected four traits that best represent vegetative and reproductive variation used in dichotomous keys of the two main *Antirrhinum* monographs (Rothmaler, 1956; Sutton, 1988). Based on the consistent use of three reproductive characters in the two keys to *Antirrhinum* species, discrete traits were selected and codified as: (1) color of the corolla (yellow or pink/purple/white); (2) corolla size (large: >30 mm; or small: <30 mm); (3) capsule size (using the midpoint of the range provided by the keys, large: >10 mm; or small: <10 mm). In addition, we reconstructed the evolution of three major morphotypes as a discrete character with three states (*Antirrhinum*, *Kickxiella*, and *Streptosepalum*; see Figure 1) based on the subsectional classification of Rothmaler (1956). Ancestral state reconstructions were conducted using a stochastic character mapping approach (SIMMAP) implemented in the R package “PHYTOOLS” (Revell, 2012; R Core Team, 2013) and using the ultrametric tree resulting from TreePL as the input (after pruning the outgroup and the four conflicting taxa as previously done for biogeographic analyses, see above). For these analyses we tested up to three models of trait evolution differing in the transition rates among states: (i) equal rates (ER; rates are equal for all transitions among states); (ii) symmetric rates (SYM; transition rates vary, but backward and forward rates for each transition are equal); and (iii) different rates (ARD; all rates are different, including different backward and forward rates for each transition). We fit Mk models using the function “fitMk” of PHYTOOLS. The three models of trait evolution were tested for the three major morphotypes. For those traits with only two states, i.e., corolla color, and corolla and capsule size, model SYM was not applicable, and therefore only ER and ARD were tested. The best-fit model for each trait was chosen according to the Akaike Information Criterion (AIC). Two hundred stochastic reconstructions were simulated using the function “make.simmap.” As a result, a summary tree of each of the 200 simulations was obtained from each character reconstruction.

### Introgression Tests

**FIGURE 3:**
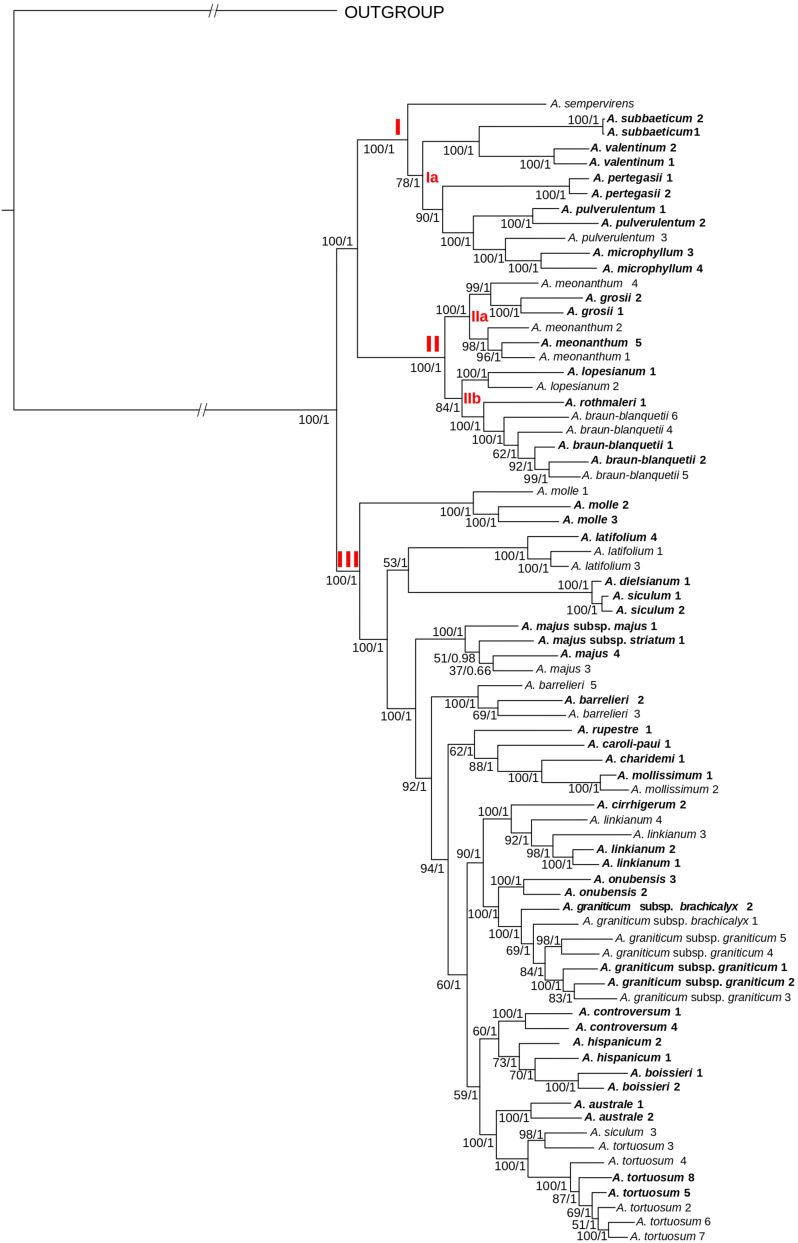
Phylogenetic reconstruction of *Antirrhinum* based on concatenated DNA sequences of 4,663 genotyping-by-sequencing loci (c90m6 dataset) obtained from a wide sample of specimens representing the geographic distribution of species. The best scoring of the maximum likelihood tree from RAxML analysis is shown. Three major clades (I–III) and subclades (Ia, IIa, and IIb) are indicated in red letters. Values at branches are bootstrap support values from the maximum likelihood (RaxML) analysis followed by posterior probabilities from the Bayesian (ExaBayes) analysis. In bold, populations collected from topotypic localities, i.e., those where the type material was collected. Further voucher information for each specimen (indicated with name and numbers) is detailed in Supplementary Table 1 of the Supplementary Files.

**FIGURE 4:**
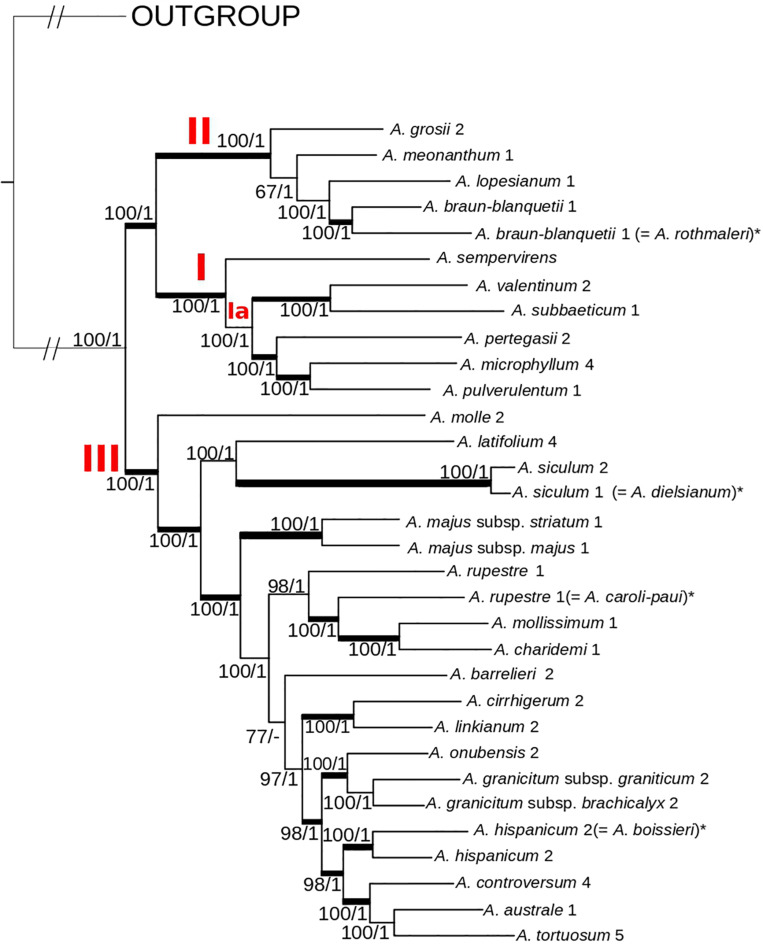
Phylogenetic relationships of the 26 *Antirrhinum* species herein recognized based on concatenated DNA sequences of 4,016 genotyping-by-sequencing loci (c90m6 dataset) obtained from topotypic specimens. The best scoring maximum likelihood tree from RAxML analysis is shown. The three major clades (I–III) and a subclade (Ia) are indicated in red letters. Values at branches are bootstrap support values from the maximum likelihood (RAxML) analysis followed by posterior probabilities from the Bayesian (ExaBayes) analysis. Thicker branches are also supported by coalescent-based analyses in SVDquartets. An asterisk (*) at some taxon names (in brackets) indicates current synonyms for particular species. Further voucher information for each specimen is detailed in Supplementary Table 1 of the Supplementary Files.

We evaluated the potential introgression between early-diverging lineages in clade III and species in clades I + II (see Figure 3 for major clades) by conducting four-taxon *D*-statistic tests (Durand et al., 2011) in PyRAD v. 3.0.6. D-statistic tests compare the occurrence of “ABBA” versus “BABA” patterns based on a four taxon pectinate topology: {[(P1, P2), P3], O} (Martin et al., 2015). Introgression either between P1 and P3 (BABA) or between P2 and P3 (ABBA) is suggested when one of those patterns is significantly more frequent than the other (Eaton et al., 2015). In particular, we tested introgression for *A. molle, A. latifolium* and *A. siculum* as suggested by previous results (Wilson and Hudson, 2011). This three species are involved in the early divergence in clade III (see Figures 3, 4). Therefore, we focused on three specific introgression hypotheses: (i) introgression between *A. molle* and clade I + II; (ii) introgression between *A. latifolium* and clade I + II; and (iii) introgression between *A. siculum* and clade I + II. To test these hypotheses, our pectinated four-taxon tree comprised: (1) the outgroup (O), which included the nine outgroup taxa of phylogenomic analyses; (2) P3, corresponding to a selection of two individuals per species from clade I + II, including at least one topotypic individual (see Supplementary Table 2); (3) P2, including all individuals from the three early-diverging lineages of clade III (*A. molle, A. latifolium*, and *A. siculum*); and (4) P1, comprising a selection of individuals from the remaining taxa of clade III following the same criteria as for P3 (see Supplementary Table 2). Combination of different individuals yielded a total of 46,980 ABBA-BABA tests. As input, we used the dataset that had produced the tree with the highest bootstrap average. Two hundred bootstrap replicates per test were run. We calculated percentages of tests with significant results (based on adjusted *p*-values calculated with the Holm–Bonferroni method; α = 0.05) for the three set of tests corresponding to the three introgression hypotheses.

## Results

### Data Assembly

Illumina sequencing provided c. 700 millions of reads for each of the two libraries, with a GC content between 42–44% and around 96% of bases with quality >Q20. FastQC analysis showed high quality of reads with low signal of adapters or other contaminants. Reference mapping assembly yielded between 237 and 6,524 loci, concatenated lengths between 78,284 bp and 1.69 Mbp, and percentages of missing data varying from 32.6 to 78.8% depending on the set of localities included (all localities or topotypic localities only) and minimum taxon coverage (see Table 2).

### Phylogenetic Analysis and Divergence Time Estimates

Topologies resulting from ML analyses were mostly congruent among the eight matrices, with differences in bootstrap support (BS) values at some nodes (Supplementary Figures 1, 2). The highest BS values were obtained for the m6 matrices (minimum coverage of six samples per locus). Consequently, these matrices were used for further phylogenetic analyses (Figures 3, 4). In general, bootstrap support values were high (the majority at 100) except for some medium-terminal nodes (BS < 70). Monophyly of multiple populations in species recognized by Sutton (1988) was reconstructed in many cases. In addition, phylogenetic distinctiveness and morphological characters led us to recognize one more species (*A. rupestre*). For the sake of brevity, we show results of 26 species herein recognized for the genus *Antirrhinum*, which includes all the species of Sutton (1988) and those recognized after this publication (see Güemes, 2009), plus *A. rupestre* (Figure 3). Additional taxonomic reassessment includes the transference of yellow-flowered plants from Morocco previously identified as *A. siculum* (Fiz et al., 2000) to the polymorphic *A. tortuosum* based on morphological characters other than flower color.

Regarding the topology of the tree including all samples, three well-supported main clades were obtained: (1) clade I, including *A. sempervirens* as the earliest-diverging lineage, sister to a subclade (subclade Ia) formed by *A. subbaeticum*, *A. valentinum*, *A. pertegasii*, *A. pulverulentum*, and *A. microphyllum* (the latter nested within *A. pulverulentum*); (2) clade II, sister to clade I, and in turn formed by the two sister subclades IIa (*A. meonanthum–A. grosii*) and IIb (*A. lopesianum–A. rothmalerii*, *A. braun-blanquetii*); and (3) clade III, containing all remaining species. The latter clade showed a repeated pattern of nested lineage differentiation in which *A. molle* was inferred to be the earliest-diverging lineage (see Figure 3).

The same three main clades were reconstructed in the tree of topotypic specimens (Figure 4). Topology was congruent except for the position of *A. barrelieri* and phylogenetic relationships among some subclades, although with moderate bootstrap support (*A. meonanthum*–*A. grosii; A. linkianum-cirrhigerum, A. graniticum-onubensis*, and *A. hispanicum*–*A. controversum–A. australe–A. tortuosum*) (Figures 3, 4). For the tree including all localities, *A. barrelieri* was inferred as an isolated early-diverging lineage, sister to *Antirrhinum* species from S and SW Iberian Peninsula plus *A. tortuosum*, whereas for tree of topotypic specimens, *A. barrelieri* was inferred as nested within the S-SW clade, although with marginal bootstrap support value (BS = 77) (Figure 4). Bayesian inference in ExaBayes yielded the same topologies as ML, and most nodes showed maximum Bayesian posterior probabilities (BPP = 1) (Figure 4). Topologies from coalescent-based analyses using the SVDquartets method were congruent with concatenation-based phylogenies (Figure 4). The three major clades were also inferred with high BS, although lower BS values were obtained for internal subclades within clade III (Supplementary Figure 3).

Estimates of divergence times obtained from TreePL are shown in Supplementary Figure 4. A stem age of 16.85 myr and a crown age of 4.77 myr were inferred for *Antirrhinum*, leading to a diversification rate estimate of 0.54 spp. Myr^–1^ assuming an extant diversity of 26 species. A stem age of 4.32 myr was inferred for diversification of clades I and II, while estimated crown ages were 2.43 and 3.49, respectively. The estimated crown age for clade III was 4.36 myr. Divergence times from the Pleistocene onward (<2 myr) were inferred for most *Antirrhinum* species, with some exceptions in the Pliocene such as *A. sempervirens*, *A. molle*, *A. siculum*, *A. latifolium*, and *A. majus* (Supplementary Figure 4).

### Biogeographic Analyses

**FIGURE 5:**
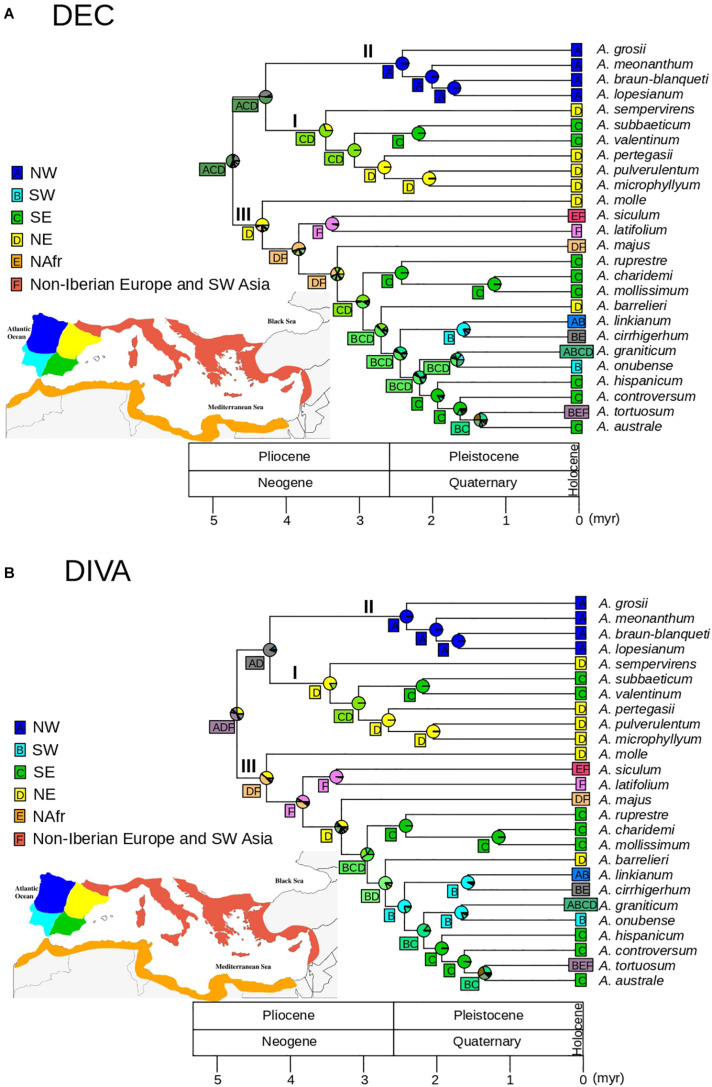
Biogeographic patterns for *Antirrhinum* estimated from the GBS phylogeny of topotypic specimens (see Figure 4) and inferred using a set of six areas previously defined: NW Iberia (A), SW Iberia (B), SE Iberia (C), NE Iberia (D), N Africa (E), and non-Iberian Europe and SW Asia (F) (see Vargas et al., 2009). Pie charts represent probabilities of alternative ancestral ranges at nodes based on the DEC **(A)** and DIVA **(B)** models. The most likely range areas at each node are indicated in a square.

The DEC model showed lower AIC values than the DIVA-like model (132.6 and 138.6, respectively), with ΔAIC > 2 (Anderson and Burnham, 2002). Overall, DEC and DIVA models resulted in biologically congruent reconstructions, although DEC resulted in lower uncertainty at some deep nodes (Figure 5). The DEC model inferred a widespread ancestral range for the genus *Antirrhinum*, most likely including the NW, NE, and SE of the Iberian Peninsula (pACD = 0.41; Figure 5A), whereas the DIVA model showed a widespread ancestral range including northern Iberia and non-Iberian Europe but with higher ambiguity (pADF = 0.33; Figure 5B). Ancestral range for the common ancestor of clades I and II was inferred to be most likely in northern and SE Iberia (pACD = 0.52) for the DEC model, and only in northern Iberia for the DIVA model (pAD = 0.84; Figure 5). Meanwhile, a NW Iberian ancestral range was congruently inferred for clade II by DEC and DIVA models (DEC: *p* = 0.99; DIVA: *p* = 0.99), while an eastern (DEC: *p* = 0.69) or NE (DIVA: pD = 0.82) Iberian ancestral range was obtained for clade I. A geographic split was inferred for the eastern widespread ancestor of the subclade Ia, which led to lineage differentiation in NE (*A. pertegasii*–*A. pulverulentum*–*A. microphyllum*) and SE (*A. subbaeticum*–*A. valentinum*) Iberia, with maximum probabilities for both DEC and DIVA models. Ancestral range for clade III was inferred as most likely in NE Iberia with (*p* = 0.44 in DIVA) or without (*p* = 0.50 in DEC) other areas. For both DEC and DIVA, lineage colonization within clade III was inferred from NE Iberia to SE Iberia, accompanied by speciation in the latter area (*A. rupestre*, *A. charidemi*, and *A. mollissimum*). Later on, the subclade formed by *A. barrelieri* and the remaining species had a widespread eastern ancestor that also colonized SW Iberia. Within this subclade, lineage colonization led to differentiation of SW lineages on one side (*A. linkianum*, *A. cirrhigerum*, and *A. onubensis*) and SE lineages on the other side (*A. hispanicum*, *A. boissieri*, *A. controversum*, and *A. australe*), while *A. graniticum* expanded to a larger area. Three independent events of colonization of northern Africa were inferred in clade III: (1) from non-Iberian Europe by *A. siculum*; (2) from SW Iberia by *A. cirrhigerum*; and (3) from SE Iberia or non-Iberian Europe leading to differentiation of the widely distributed *A. tortuosum* (Figure 5). Lineage expansion was inferred for *A. linkianum* because it has an ancestral origin in SW Iberia, with subsequent colonization of NW Iberia.

### Ancestral State Reconstruction

**FIGURE 6:**
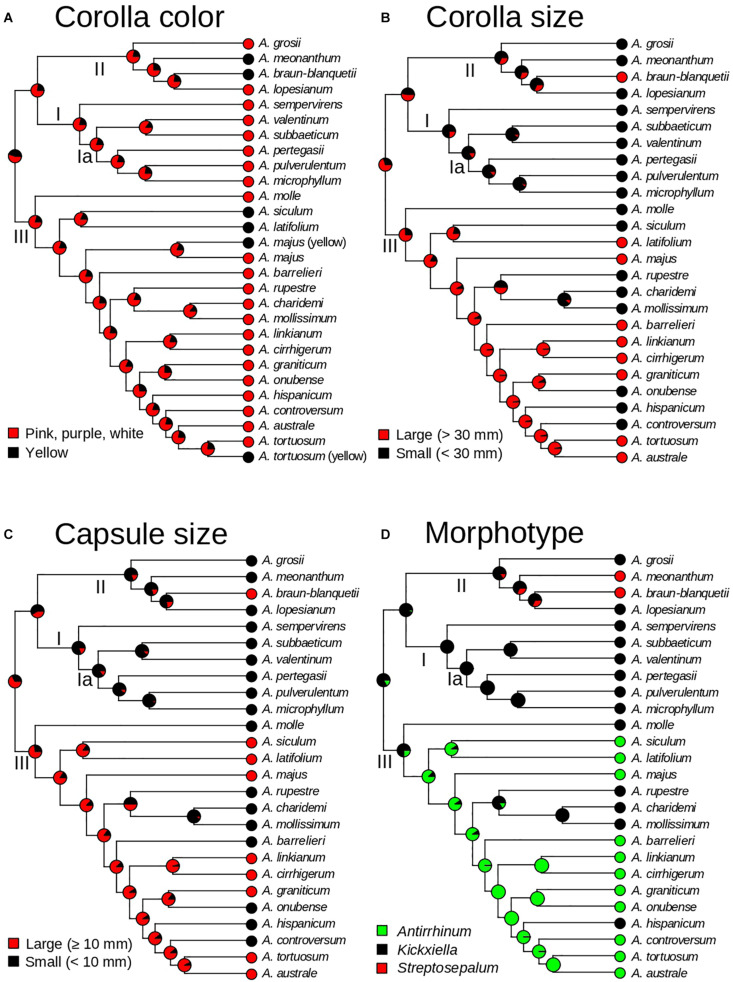
Evolution of morphological traits of *Antirrhinum* based on a stochastic character mapping approach (SIMMAP) and the GBS phylogeny of topotypic specimens (see Figure 4). Pie charts represent probabilities of alternative ancestral states at nodes after analyses of four traits: corolla color **(A)**, corolla size **(B)**, capsule size **(C)**, and morphotype **(D)**. Morphotypes are defined according to the three taxonomic subsections of Rothmaler (1956).

Ancestral state reconstruction estimated pink/purple/white corollas as the most likely state for all ancestors, with yellow corollas evolving six times independently (in *A. meonanthum*, *A. braun-blanquetii*, *A. siculum–A. latifolium*, and yellow-flowered populations of *A. tortuosum* and *A. majus*; Figure 6A). Large corollas were inferred as the most likely ancestral state for *Antirrhinum*, with multiple changes to smaller ones during the evolutionary history of the genus (Figure 6B). Acquisition of small corollas took place in the ancestors of clades I and II, but not clade III. This state was maintained to the present in all extant species of clades I and II except for *A. braun-blanquetii*, which showed a recent reversion to large corollas (Figure 6B). Within clade III at least six changes to small corollas were inferred (Figure 6B). Shifts in capsule size were inferred from the ancestral state of large capsules to small sizes for Clades I and II including a reversion to large size for *A. braun-blanquetii* (Figure 6C). Clade III maintained the ancestral state of large capsule with at least six shifts to small capsules toward the tips (*A. molle*, *A. rupestre–A. charidemi–A. mollissimum*; *A. barrelieri*; *A. onubense*; *A. hispanicum*; and *A. controversum*; Figure 6C). The *Kickxiella* morphotype (small prostate and xerophytic woody plants) was inferred as the most likely ancestral habit not only for the genus but also for the three main clades. The *Antirrhinum* morphotype (upright tall habit, long thin leaves with no hairs and magenta or yellow flowers growing in sandy soils) was acquired soon after divergence of clade III, and reverted twice to the *Kickxiella* morphotype in the evolution of four species. The *Streptosepalum* morphotype appeared to have evolved twice, giving rise to two species of clade II (Figure 6D).

### Introgression Tests

*D*-statistic analyses showed no significant ancestral introgression between any of the three early-diverging lineages of clade III (*A. molle, A. latifolium*, and *A. siculum*) and the clades I + II (see Table 3). However, ABBA-BABA tests suggested a certain level of introgression between remaining species of clade III and certain lineages of clade II. In particular, *A. lopesianum* was involved in 276 of 406 tests that yielded significant results for introgression of clade II and lineages from clade III (see Supplementary Table 2). None of the individuals of clade I was suggested to have experienced significant introgression with clade III.

**TABLE 3:** *D*-statistic summary for the introgression test given a four-taxon tree {[(P1,P2),P3],O}.

		Proportion of significative tests for introgression with clade I + II (P3)
**Early-diverging lineages of clade III (P2): BABA pattern**	Hypothesis I: *A. molle*	0
	Hypothesis II: *A. latifolium*	0
	Hypothesis III: *A. siculum*	0
**Remaining lineages of clade III (P1): ABBA pattern**	Hypothesis I: *A. molle*	105/15660
	Hypothesis II: *A. latifolium*	227/15660
	Hypothesis III: *A. siculum*	74/15660

*Proportion of significant tests between individuals of clade I and II (P3) and either early-diverging individuals from clade III (P2; BABA pattern) or remaining individuals from clade III (P1; ABBA pattern).*

## Discussion

A phylogenetic structure of *Antirrhinum* has been elusive for a long time. Possible causes for lack of phylogenetic resolution included rapid speciation and hybridization (Vargas et al., 2004, 2009). In particular, *Antirrhinum* phenotypes seemed to be constrained by selection, which might reflect contrasting adaptations to life on bare rock faces or sandy soils (Wilson and Hudson, 2011). Indeed, recent speciation and hybridization appear to be the main causes of plant diversity in Mediterranean-type ecosystems (Rundel et al., 2016), and they may also be responsible for complex taxonomy of angiosperms (Vargas et al., 2018). Nevertheless, none of the previous phylogenetic studies of *Antirrhinum* included a considerable number of type or topotypic materials to contrast hypotheses (but see Vargas et al., 2009). This has also prevented the accurate naming of some *Antirrhinum* populations. In contrast, our study offers a new phylogenetic hypothesis that helps to elucidate systematics and evolution thanks to a high number of phylogenetically informative nucleotide characters (>50,000 bp) taken from genome-wide (GBS) sequences, together with DNA sequences that are anchored to original *Antirrhinum* names (Bell et al., 2020).

Our approach leads to strongly encourage researchers in plant systematics to not only use a high number of loci (as provided by the GBS technique) but also include topotypic material in phylogenetic analyses to ensure a correct naming of populations and stable evolutionary inferences.

### Contribution to the Systematics of *Antirrhinum*

The family of restriction digest-based techniques (including GBS, RAD-Seq and similar techniques) is highly recommended for studies in systematics of diverse plant groups inasmuch as the combination of cost-effectiveness, high resolution and strong statistical support enables a robust overview of phylogenetic relationships among lineages. However, the nature of reduced representation sequencing based on restriction sites can result in high rates of missing data in concatenated matrices and lower repeatability of targeted genomic regions (Harvey et al., 2016). In our case, the percentages of missing data are similar to those analyzed in other studies that also used GBS data (e.g., Fernández-Mazuecos et al., 2018; Martín-Hernanz et al., 2019) and found robust phylogenetic support after discarding some low quality samples (see section “Results”).

The two worldwide taxonomic treatments of *Antirrhinum* (Rothmaler, 1956; Sutton, 1988) included species circumscriptions that mostly agree with our phylogenetic results. However, our results suggest a number of taxonomic re-arrangements, particularly at the species level. On the one hand, some species recognized by Rothmaler (1956) were not considered by Webb (1971) and Sutton (1988) or further authors, but should be re-visited (Table 1). On the other hand, new narrow endemic species found in the field were described after Sutton (1988), recognized in more recent floras (e.g., Güemes, 2009) and supported by our phylogenetic analyses. For the sake of brevity, we next discuss major phylogenetic results that disagree with those four treatments.

#### Monophyly and Species Recognition

Numerous (17) monophyletic groups of populations were congruently associated with current species (Vargas et al., 2004; Carrió et al., 2010; Wilson and Hudson, 2011). Some other species (4) fall in a pattern of paraphyly (*A. microphyllum* embedded in *A. pulverulentum* lineages; *A. grosii* embedded in *A. meonanthum* lineages) following Sutton’s (1988) circumscription of species (Figure 3). This suggests a pattern of budding speciation typically found in Iberia (Otero et al., 2019). The hypothesis of a general pattern of budding speciation in *Antirrhinum* needs to be tested with a higher number of populations, particularly from widespread species.

#### Infrageneric Taxa

The hypothesis of three main groups (subsections) of *Antirrhinum* proposed by Rothmaler (1956), which was not adopted by either Webb (1971) or Sutton (1988), is not supported by our phylogenetic analyses. The phylogenetic structure of *Antirrhinum* based on GBS data revealed two main clades (I + II and III, see Figures 3, 4), both of them with species of subsect. *Kickxiella* (Figure 3). In particular, the two species of subsect. *Streptosepalum* (*A. braun-blanquetii* and *A. meonanthum*) grouped together with most species of subsect. *Kickxiella*, whereas all the species of subsect. *Antirrhinum* grouped together with a few species of subsect. *Kickxiella*. This is partially congruent with Wilson and Hudson (2011) and prevents us from accepting any subsections based solely on classical morphological characters.

#### *Antirrhinum majus* Group

The type species of the genus is *A. majus* (Sutton, 1988). This species is an important model for plant genetics, development and evolution (see Schwarz-Sommer et al., 2003; Li et al., 2019). Despite the importance of this model plant, taxonomy of the “*A. majus* group” has neither met consensus yet nor been revisited using molecular phylogenetics. During most of the 20th century, researchers primarily considered six subspecies (*barrelieri*, *cirrhigerum, linkianum*, *majus*, *striatum*, and *tortuosum*) circumscribed in the *A. majus* group (Rothmaler, 1956; Webb, 1971; Sutton, 1988). The use of topotypic samples reveals that all these taxa do not form a natural group because they are placed in different subclades of clade III (Figure 4). This result agrees with a taxonomic treatment at the species level following the most recent treatment of *Antirrhinum* for the Iberian Peninsula (Güemes, 2009). The question remains as to whether laboratories using *Antirrhinum* as a model plant are employing plants correctly assigned to the true *A. majus* or any other species of the former *A. majus* group.

#### Further Taxonomic Research

Our phylogenetic results help taxonomic decision making at the supraspecific level, but also suggest further investigation in certain subclades that contain poorly studied species: (i) *A. rupestre* and *A. caroli-paui* are basal-most lineages of the subclade of *A. mollissimum*–*A. charidemi*; (ii) *A. striatum* is certainly unrelated to *A. latifolium* (see Liberal et al., 2014) and now considered within *A. majus* at the subspecies level (Khimoun et al., 2013); and (iii) four taxa described as independent species (*A. bolosii*, *A. fernandezcasasii*, *A. saccharatum*, and *A. ternatum*) need to be phylogenetically analyzed for systematic purposes given that there is no consensus among authors. High uncertainty about these species is illustrated by the fact that the same author who first described *A. bolosii* (Fernández-Casas, 1972) did not consider it shortly after (Fernández-Casas, 1974). In addition, as these species have never been included in a key to species of *Antirrhinum* or in a comparative table, a taxonomic study using their type specimens and topotypic populations is needed.

### Radiation and Geographical Speciation of *Antirrhinum*

The hypothesis of an evolutionary radiation of *Antirrhinum* based on ITS and plastid sequences (Vargas et al., 2009) is supported by our phylogenetic analysis based on GBS data, which found a high rate of diversification (0.54 spp. Myr^–1^) into at least 26 species (Valente et al., 2010; Bell et al., 2012) since the Pliocene (Vargas et al., 2009). The combination of terrain complexity and eco-climatic novelty seems to explain why the Mediterranean basin contains numerous examples of explosive radiations in the angiosperms (Valente et al., 2010).

The hypothesis of a primarily geographic pattern of snapdragon speciation in Iberia has been put forward based on: (i) endemicity for the majority of *Antirrhinum* species (Vargas et al., 2009); (ii) numerous narrow, endangered endemics in small mountain ranges (Carrió et al., 2010); (iii) a high number of unique haplotypes and haplotype clades restricted to small geographic areas (Vargas et al., 2009). In particular, previous phylogeographic results are supported by our biogeographic analysis (Figure 5), in which a likely primary center of diversification in northern Iberia (or even out of the Iberian Peninsula) was followed by a secondary center of diversification in SE Iberia (Vargas et al., 2009). Six speciation events occurred unequivocally in SE Iberia during the Quaternary based on our biogeographic reconstruction and time-calibrated phylogeny (Figure 5). The mountains of eastern Andalusia (SE Iberia) contain one of the richest areas of Europe in terms of number of species and endemics (Cueto et al., 2018) and form indeed a main center of recent angiosperm diversification (Buira et al., 2020). One more argument for the strong pattern of recent geographic speciation in SE Iberia is shown by the considerable number of sister species pairs (*A. subbaeticum*–*A. valentinum*; *A. mollissimum*–*A. charidemi*; *A. australe*–*A. tortuosum*) in nearby areas (Figure 3). In addition, two more clades of NW and NE Iberian species support a strong pattern of geographic speciation for *Antirrhinum* (Vargas et al., 2009), which fits into a general pattern for Mediterranean plants. A pattern of geographic differentiation in the Mediterranean appears to be the rule rather than the exception because of unique opportunities for spatial isolation given the numerous islands, peninsulas, and high mountains of southern Europe. In particular, the complex geography and orography of Iberia provides a suitable spatial framework for speciation (Rundel et al., 2016; Vargas et al., 2018). Spatial differentiation has also been documented for early stages of speciation between *A. majus* subsp. *majus* and subsp. *striatum* separated by mountains (Pujol et al., 2017), albeit ecological conditions may have secondarily contributed (Khimoun et al., 2013). One more line of evidence that supports predominant speciation by geographic isolation reinforced by ecological factors is given by a strong geographic pattern of pollinator types associated with the bee-specialized flowers of *Antirrhinum* (Vargas et al., 2010). In particular, two pollinator systems (Mediterranean vs. temperate areas of Europe) were proposed based on two consistent pollinator niches that include 11 bee species and most *Antirrhinum* species (Vargas et al., 2017).

### Hybridization vs. Convergent Evolution of Key Morphological Characters

None of the states of the three morphological characters (corolla color, corolla size, and fruit size) used in all taxonomic keys to *Antirrhinum* species turned out to be synapomorphic (Figure 6). This result was already observed in previous analyses, but poor resolution prevented from a detailed description of character homoplasy (Vargas et al., 2004; Wilson and Hudson, 2011). Our ancestral state reconstruction analyses revealed a strong pattern of convergent evolution for corolla color, corolla size, and capsule size (Figure 6). One of the fastest and most frequent mechanisms of phenotypic convergence is hybridization (Stern, 2013). Hybridization in *Antirrhinum* has been extensively documented based on the observation of plants with intermediate morphological characters between co-occurring species (see Güemes, 2009), production of viable offspring from crosses between multiple species (Günther and Rudolph, 1970) and molecular results (Vargas et al., 2009). In particular, plastid haplotype sharing among species (Vargas et al., 2009; Wilson and Hudson, 2011) and the occurrence of numerous nucleotide additivities in nuclear ribosomal ITS sequences (Vargas et al., 2009; Carrió et al., 2010) have been interpreted as evidence of recent hybridization in *Antirrhinum*. Indeed, both recent and ancient hybridization events have been proposed based on shallow and profound incongruence between morphology, plastid, and nuclear markers, which produce large phylogenetic polytomies (Vargas et al., 2004, 2009; Carrió et al., 2010; Wilson and Hudson, 2011). Our analyses to test a hybridization signal between main clades potentially resulting in the early-diverging position of three species of clade III (*A. molle, A. latifolium*, and *A. siculum*) failed to find a significant result. More recent hybridization may be easier to reconstruct based on the genetic makeup of current populations. At this level, no unequivocal support for hybridization was, however, found for some species of *Antirrhinum* based on nuclear fingerprints (RAPD) and morphology (Jiménez et al., 2005). Along the same lines, unique allozyme profiles were interpreted as long-term isolation between three species with similar morphotypes (Mateu-Andrés, 1999), while *A. charidemi* also shows unique plastid sequences and SSR profiles (Forrest et al., 2017). Sporadic hybrids in the field, together with rare cases of hybrid swarms indicate that hybridization between main clades is not currently playing an important role in *Antirrhinum* (Sutton, 1988; Mateu-Andrés, 1999). Indeed, reproductive experiments revealed that post-zygotic barriers are at play at least for two co-occurring species of *Antirrhinum* (*A. controversum*, *A. valentinum*) that are distant in our phylogenetic reconstruction (Carrió and Güemes, 2014).

In sum, our study provides a clear cladogenetic pattern for *Antirrhinum* thanks to a high number of loci obtained with the GBS technique. Besides, stability of the phylogeny is guaranteed by the use of topotypic material anchored to original *Antirrhinum* names. Lack of resolution using less variable DNA sequences appear to have primarily been the result of evolutionary radiation in the Pleistocene rather than hybridization between plants of different major lineages. A strong signal of monophyly and phylogenetic distinctiveness for currently recognized species was obtained, but the analysis of a higher number of populations is still needed for a more solid proposal of the systematics of snapdragons. Although some plants may have a hybrid origin, intermediate key morphological characters (corolla color and size, fruit size) in numerous species are better explained by parallel evolution due to gene reutilization, a hypothesis that has been put forward for some model genera including *Antirrhinum* (Preston et al., 2011). All sources of data strongly suggest a general pattern of divergence promoted by isolation in mountains, which accounts for a high number of endemics to the Iberian Peninsula. Geographical isolation is, therefore, the most plausible driver of speciation in *Antirrhinum*, followed by ecological factors such as pollination systems, soil preference and climate conditions.

## Data Availability Statement

The data that support the findings of this study are openly available in Short Read Archive (SRA), under the reference number PRJNA690200. SRA accessions for each sample are indicated in Supplementary Material at Supplementary Table 1.

## Author Contributions

PV conceived the idea and drafted the manuscript. AO led lab work. AO conducted the data analysis and summarized results with contributions of MF-M. PV, AO, and MF-M interpreted and discussed the results, and wrote the manuscript. All authors contributed to the article and approved the submitted version.

## Conflict of Interest

The authors declare that the research was conducted in the absence of any commercial or financial relationships that could be construed as a potential conflict of interest.

## Footnotes

1 https://www.iapt-taxon.org/nomen/main.php

## Supplementary Material

Supplementary Figure 1 The best scoring maximum likelihood tree from RAxML analysis of all *Antirrhinum* taxa set under three different numbers of minimum taxa for a locus: m4, m18, and m36. Bootstrap support values are indicated at the nodes.

Supplementary Figure 2 The best scoring maximum likelihood tree from RAxML analysis of *Antirrhinum* topotypic specimens under three different numbers of minimum taxa for a locus: m4, m18, and m36. Bootstrap support values are indicated at the nodes.

Supplementary Figure 3 Consensus coalescent-based tree obtained from SVDquartets. Individuals were grouped according to current species circumscriptions. Bootstrap values are indicated at the nodes.

Supplementary Figure 4 Time-calibrated tree of topotypic specimens of *Antirrhinum* obtained from TreePL analysis. Inferred ages are indicated at nodes.

Supplementary Table 1 Data information and NCBI SRA accessions of all individuals sampled for GBS.

Supplementary Table 2 Significant *D*-statistic tests given a four-taxon tree {[(P1,P2),P3],O}. Abbreviation for each individual is described at Supplementary Table 1. Tests are differentiated according to the three introgression hypotheses tested (MOLLE: hybrid origin for *A. molle*; LAT: hybrid origin for *A. latifolium*; and *SIC*: hybrid origin for *A. siculum*). *D*-statistic value (D), standard deviation [(std. (D)], *z*-score (Z), proportion of BABA and ABBA, number of loci involved for each test (nloci), number of bootstrap replicates (nboot), significant pattern (pattern), *p*-value and adjusted *p*-value through Bonferroni–Holm method are shown.

Supplementary Data Sheet 1 Locations of *Antirrhinum* photographs included in Figure 2 of the main text.
