# Supplementary material for: Evolution in the Model Genus Antirrhinum Based on Phylogenomics of Topotypic Material
Source: Front Plant Sci. 2021 Feb 12;12:631178. doi: 10.3389/fpls.2021.631178 (PMC7907437; doi:10.3389/fpls.2021.631178)
Supplement: Supplementary Figure 1 — The best scoring maximum likelihood tree from RAxML analysis of all Antirrhinum taxa set under three different numbers of minimum taxa for a locus: m4, m18, and m36. Bootstrap support values are indicated at the nodes. [file Data_Sheet_1.docx]

Supplementary Material

# Supplementary Figures and Tables

##

## Figure S1. The best scoring maximum likelihood tree from RAxML analysis of all *Antirrhinum* taxa set under three different numbers of minimum taxa for a locus: m4, m18 and m36. Bootstrap support values are indicated at the nodes.

**Figure S2.** The best scoring maximum likelihood tree from RAxML analysis of *Antirrhinum* topotypic specimens under three different numbers of minimum taxa for a locus: m4, m18 and m36. Bootstrap support values are indicated at the nodes.

**Figure S3.** Consensus coalescent-based tree obtained from SVDquartets. Individuals were grouped according to current species circumscriptions. Bootstrap values are indicated at the nodes.

**Figure S4.** Time-calibrated tree of topotypic specimens of *Antirrhinum* obtained from TreePL analysis using m6 DNA matrix. Inferred ages are indicated at nodes.

**Table S1.** Data information and NCBI SRA accessions of all individuals sampled for GBS. Letter “C” at specimen code mark those topotypic specimens. Asterisk (*) indicate those samples excluded from the final sampling because of poor DNA yielded from sequencing procedure.

| **Taxa** | **Voucher** | **Specimen number** | **Location (Abrev.)** | **Specimen code** | **Genbank accession** |
| --- | --- | --- | --- | --- | --- |
| *Acanthorrhinum ramosissimum* | VAL41469 | 1 | Morocco | Aca_ram_1* | - |
| *Antirrhinum ambiguum* | 105PV19(1) | 1 | Cerro Las Machotas, supra Escorial, Madrid, Spain (MAD) | Ant_amb_1C* | - |
| *Antirrhinum ambiguum* | 105PV19(2) | 2 | Cerro Las Machotas, supra Escorial, Madrid, Spain (MAD) | Ant_amb_2C | SAMN17245960 |
| *Antirrhinum australe* | 3IML12(1) | 1 | Benacoaz, Cádiz, Spain (CAD) | Ant_aus_1C | SAMN17245961 |
| *Antirrhinum australe* | 3IML12(2) | 2 | Benacoaz, Cádiz, Spain (CAD) | Ant_aus_2C | SAMN17245962 |
| *Antirrhinum australe* | 44PV12(2) | 3 | Santiago de la espada, Jaén, Spain (JAE) | Ant_aus_3* | - |
| *Antirrhinum australe* | 38IML11(2) | 4 | Montizón, Jaén, Spain (JA) | Ant_aus_4* | - |
| *Antirrhinum barrelieri* | MA558603 | 1 | Sestrica, Zaragoza, Spain (ZA) | Ant_lit_1C* | - |
| *Antirrhinum barrelieri* | 141PV08(4) | 2 | Nuévalos, Zaragoza, Spain (ZA) | Ant_lit_2C | SAMN17245993 |
| *Antirrhinum barrelieri* | 39PV11(12) | 3 | La Senia, Tarragona, Spain (TA) | Ant_lit_3 | SAMN17245994 |
| *Antirrhinum barrelieri* | 9PV17(5) | 4 | Navarra, Spain (NA) | Ant_lit_4* | - |
| *Antirrhinum barrelieri* | 2IML13(3) | 5 | Vallbona, Valencia, Spain (VAL) | Ant_lit_5 | SAMN17245995 |
| *Antirrhinum boissieri* | 6IML13(1) | 1 | La Alhambra, Granada, Spain (GR) | Ant_boi_1C | SAMN17245963 |
| *Antirrhinum boissieri* | 6IML13(2) | 2 | La Alhambra, Granada, Spain (GR) | Ant_boi_2C | SAMN17245964 |
| *Antirrhinum boissieri* | 180PV06 | 3 | Mancha Real, Jaén, Spain (JA) | Ant_boi_3* | - |
| *Antirrhinum braun-blanquetii* | MA777373 | 1 | La Guiana, Los Apóstoles, Ponferrada, León, Spain (LE) | Ant_bra_1C | SAMN17245965 |
| *Antirrhinum braun-blanquetii* | MA345884 | 2 | La Guiana, Los Apóstoles, Ponferrada, León, Spain (LE) | Ant_bra_2C | SAMN17245966 |
| *Antirrhinum braun-blanquetii* | 20PV17(3) | 3 | Palencia, Spain (PAL) | Ant_bra_3* | - |
| *Antirrhinum braun-blanquetii* | 47IML11(1) | 4 | Bielva, Cantabria, Spain (CA) | Ant_bra_4 | SAMN17245967 |
| *Antirrhinum braun-blanquetii* | LEB100719 | 5 | León, Palacios de Compludo, León , Spain (LE) | Ant_bra_5 | SAMN17245968 |
| *Antirrhinum braun-blanquetii* | LEB100387 | 6 | León, Benuza, Portillo de Ferradillo, León, Spain (LE) | Ant_bra_6 | SAMN17245969 |
| *Antirrhinum caro-pauli* | 53PV07 | 1 | Abrucena, Almería, Spain (ALM) | Ant_car_1C | SAMN17245970 |
| *Antirrhinum caro-pauli* | 116JB10(5) | 2 | Abrucena, Almería, Spain (ALM) | Ant_car_2C* | - |
| *Antirrhinum charidemi* | OAPV05 | 1 | Vela Blanca, Almería, Spain (ALM) | Ant_cha_1C | SAMN17245971 |
| *Antirrhinum charidemi* | 8PV05 (10) | 2 | Barranco del Sabinal, Almería, Spain (ALM) | Ant_cha_2C* | - |
| *Antirrhinum cirrhigerum* | 3AO19(4) | 1 | Vilanova Milfontes, Portugal (PO) | Ant_cir_1C* | - |
| *Antirrhinum cirrhigerum* | 3AO19(7) | 2 | Vilanova Milfontes, Portugal (PO) | Ant_cir_2C | SAMN17245972 |
| *Antirrhinum cirrhigerum* | 1IML12(6) | 3 | Caños de Meca, Cádiz, Spain (CAD) | Ant_cir_3* | - |
| *Antirrhinum controversum* | 32PV08 | 1 | Cómpeta, Málaga, Spain (MA) | Ant_con_1C | SAMN17245973 |
| *Antirrhinum controversum* | 1IML11(8) | 2 | Mojácar-Carboneras, Almería, Spain (ALM) | Ant_con_2* | - |
| *Antirrhinum controversum* | 24PV12(1) | 3 | Caravaca de la Cruz, Murcia | Ant_con_3* | - |
| *Antirrhinum controversum* | MA855349 | 4 | Pizarra, Málaga, Spain (MA) | Ant_con_4C | SAMN17245974 |
| *Antirrhinum controversum* | MGC67537 | 5 | Álora de Hacho, Málaga, Spain (MA) | Ant_con_5* | - |
| *Antirrhinum controversum* | MGC67510 | 6 | Álora de Hacho, Málaga, Spain (MA) | Ant_con_6* | - |
| *Antirrhinum dielsiannum* | MA646409 | 1 | Sicilia, Siracusa, Italy (ITA) | Ant_die_1C | SAMN17245975 |
| *Antirrhinum graniticum* | 1AO19(2) | 1 | Soalheira, Portugal (PO) | Ant_gra_1C | SAMN17245976 |
| *Antirrhinum graniticum* | 2AO19(2) | 2 | Muralla castillo Castelo Branco, Portugal (PO) | Ant_gra_2C | SAMN17245977 |
| *Antirrhinum graniticum* | 6AO19(1) | 3 | Río Corgo, Povoaçao, Vila Real, Portugal (PO) | Ant_gra_3 | SAMN17245978 |
| *Antirrhinum graniticum* | 109PV19(2) | 4 | Colmenar, Madrid, Spain (MAD) | Ant_gra_4 | SAMN17245979 |
| *Antirrhinum graniticum* | 29PV07 | 5 | Guadix, Granada, Spain (GR) | Ant_gra_5* | - |
| *Antirrhinum graniticum* | 71PV10 | 6 | Alhama de Aragón, Zaragoza, Spain (ZA) | Ant_gra_6* | - |
| *Antirrhinum graniticum subsp. brachycalyx* | 102PV19(1) | 1 | Laguna de San Juan, Madrid, Spain (MAD) | Ant_gra_bra_1 | SAMN17245980 |
| *Antirrhinum graniticum subsp. brachycalyx* | 104PV19(14) | 2 | Valdemoro-Ciempozuelos, Madrid, Spain (MAD) | Ant_gra_bra_2C | SAMN17245981 |
| *Antirrhinum grosii* | 133PV10(1) | 1 | Riscos del Morezón, Ávila, Spain (AV) | Ant_gro_1C | SAMN17245982 |
| *Antirrhinum grosii* | 138PV10(7) | 2 | Riscos del Morezón, Ávila, Spain (AV) | Ant_gro_2C | SAMN17245983 |
| *Antirrhinum hispanicum* | 19IML12(2) | 1 | Barranco de las Víboras, Granada, Spain (GR) | Ant_his_1C | SAMN17245984 |
| *Antirrhinum hispanicum* | 7IML13(3) | 2 | Güéjar-Sierra, Granada, Spain (GR) | Ant_his_2C | SAMN17245985 |
| *Antirrhinum latifolium* | G1PV16 | 1 | Grenoble, France (FRA) | Ant_lat_1 | SAMN17245986 |
| *Antirrhinum latifolium* | 108PJM12(2) | 2 | Cuneo, Santa Vittoria d'Alba, Italy (ITA) | Ant_lat_2* | - |
| *Antirrhinum latifolium* | 108PJM12(4) | 3 | Cuneo, Santa Vittoria d'Alba, Italy (ITA) | Ant_lat_3 | SAMN17245987 |
| *Antirrhinum latifolium* | PI 009621 | 4 | Cave Monte Rambolo, Campliglia Maritima, Italy (ITA) | Ant_lat_4C | SAMN17245988 |
| *Antirrhinum linkianum* | 3MF13(2) | 1 | Sintra, Portugal (PO) | Ant_lin_1C | SAMN17245989 |
| *Antirrhinum linkianum* | 3MF13(5) | 2 | Sintra, Portugal (PO) | Ant_lin_2C | SAMN17245990 |
| *Antirrhinum linkianum* | 2MF13(2) | 3 | Tafaria, Portugal (PO) | Ant_lin_3 | SAMN17245991 |
| *Antirrhinum linkianum* | 32PV15(1) | 4 | Coimbra, Portugal (PO) | Ant_lin_4 | SAMN17245992 |
| *Antirrhinum lopesianum* | MA824747 | 1 | Vimioso, Portugal (PO) | Ant_lop_1C | SAMN17245996 |
| *Antirrhinum lopesianum* | 9IML11(5) | 2 | Aldeávila re la Ribera-Corporatorio, Salamanca, Spain (SA) | Ant_lop_2 | SAMN17245997 |
| *Antirrhinum lopesianum* | SALA88695 | 3 | Vimioso,Argoselo, Portugal | Ant_lop_3C* | - |
| *Antirrhinum lopesianum* | SALA88688 | 4 | Vimioso,Argoselo, Portugal | Ant_lop_4C* | - |
| *Antirrhinum majus* subsp*.* | 46PV12(1) | 1 | Osona, Barcelona, Spain (B) | Ant_maj_1C | SAMN17245998 |
| *Antirrhinum majus* subsp*.* | 25PV09 | 2 | Cotefablo, Huesca, Spain (HU) | Ant_maj_2C* | - |
| *Antirrhinum majus* subsp*. majus* | 126PV10(1) | 3 | Urzainqui, Navarra, Spain (NA) | Ant_maj_3 | SAMN17245999 |
| *Antirrhinum majus* subsp*. majus* | 230PV06 | 4 | Saint. Chinian, France (FRA) | Ant_maj_4C | SAMN17246000 |
| *Antirrhinum majus* subsp*. majus* | 165E_18_egea(2) | 5 | Cultivated: line 165 E Marcos Egea | Ant_maj_5* | - |
| *Antirrhinum majus* subsp*. striatum* | 55PV07(1) | 1 | Limoes, France (FRA) | Ant_str_1C | SAMN17246025 |
| *Antirrhinum majus* subsp*. striatum* | MA588941 | 2 | Le Caunil, France (FRA) | Ant_str_2C* | - |
| *Antirrhinum meonanthum* | 4AO19(1) | 1 | Río Corgo, Povoaçao, Vila Real, Portugal (PO) | Ant_meo_1 | SAMN17246001 |
| *Antirrhinum meonanthum* | 5AO19(1) | 2 | Río Sordo, cerca Ribeirões, Portugal (PO) | Ant_meo_2 | SAMN17246002 |
| *Antirrhinum meonanthum* | 685RSV18 | 3 | Candelario/Navacarros Salamanca, Spain (SA) | Ant_meo_3* | - |
| *Antirrhinum meonanthum* | 309PV13(1) | 4 | Aldeávila de la Ribera, Salamanca, Spain (SA) | Ant_meo_4 | SAMN17246003 |
| *Antirrhinum meonanthum* | PO60051 | 5 | Portugal, Cinfães, Portugal (PO) | Ant_meo_5C | SAMN17246004 |
| *Antirrhinum microphyllum* | 39PV08(2) | 3 | Buendía, Cuenca, Spain (CU) | Ant_mic_3C | SAMN17246005 |
| *Antirrhinum microphyllum* | 40PV08(2) | 4 | Bolarque, Cuenca, Spain (CU) | Ant_mic_4C | SAMN17246006 |
| *Antirrhinum molle* | 75PV10(15) | 1 | Gabasa, Huesca, Spain (HU) | Ant_mol_1 | SAMN17246007 |
| *Antirrhinum molle* | MA895768 | 2 | Sierra del Cadí, Lleida, Spain (LL) | Ant_mol_2C | SAMN17246008 |
| *Antirrhinum molle* | MA756423 | 3 | Andorra | Ant_mol_3C | SAMN17246009 |
| *Antirrhinum mollissimum* | 73PV06 | 1 | Barranco del Caballar, Almería, Spain (ALM) | Ant_molli_1C | SAMN17246010 |
| *Antirrhinum mollissimum* | 277PV13 (2) | 2 | Enix, Almería, Spain (ALM) | Ant_molli_2 | SAMN17246011 |
| *Antirrhinum mollissimum* | MA427678(2) | 4 | Muros Ciudad, Almería, Spain (ALM) | Ant_molli_4C* | - |
| *Antirrhinum onubensis* | 104PV09 | 3 | Aracena, Huelva, Spain (HUE) | Ant_onu_3C | SAMN17246012 |
| *Antirrhinum onubensis* | 146PJM13(3) | 2 | Aracena, Huelva, Spain (HUE) | Ant_onu_2C | SAMN17246013 |
| *Antirrhinum pertegasii* | 36PV11 | 1 | La senia, Tarragona, Spain (TA) | Ant_per_1C | SAMN17246014 |
| *Antirrhinum pertegasii* | 38PV11(12) | 2 | La pobla de Benifassa, Catellón, Spain (CAS) | Ant_per_2C | SAMN17246015 |
| *Antirrhinum pulverulentum* | 15IML12(4) | 1 | Nuevalos, Zaragoza, Spain (ZA) | Ant_pul_1C | SAMN17246016 |
| *Antirrhinum pulverulentum* | 15IML12(5) | 2 | Nuevalos, Zaragoza, Spain (ZA) | Ant_pul_2C | SAMN17246017 |
| *Antirrhinum pulverulentum* | 17IML12(1) | 3 | Sigüenza, Guadalajara, Spain (GU) | Ant_pul_3 | SAMN17246018 |
| *Antirrhinum rothmalerii* | 12IML12(4) | 1 | Macedo de Cavaleiros, Portugal (PO) | Ant_rot_1C | SAMN17246019 |
| *Antirrhinum rothmalerii* | 12IML12(2) | 2 | Macedo de Cavaleiros, Portugal (PO) | Ant_rot_2C* | - |
| *Antirrhinum rupestre* | 9IML13(3) | 1 | Trévelez, Sierra Nevada, Granada, Spain (GR) | Ant_rup_1C | SAMN17246020 |
| *Antirrhinum rupestre* | 9IML13(5) | 2 | Trévelez, Sierra Nevada, Granada, Spain (GR) | Ant_rup_2C* | - |
| *Antirrhinum sempervirens* | 104PV10(7) | 1 | Panticosa, Huesca, Spain (HU) | Ant_sem_1 | SAMN17246021 |
| *Antirrhinum sempervirens* | R289994JACA | 2 | Aragnouet, France (FRA) | Ant_sem_2C* | - |
| *Antirrhinum sempervirens* | 375980JACA | 3 | Cirque de Moundelhs, France (FRA) | Ant_sem_3C* | - |
| *Antirrhinum sempervirens* | 353397JACA | 4 | Valle de Remuñe, Huesca, Spain (HU) | Ant_sem_4* | - |
| *Antirrhinum siculum* | MA705546 | 1 | Sicilia, Palermo, Italy (ITA) | Ant_sic_1C | SAMN17246022 |
| *Antirrhinum siculum* | 33BISPV2015(1) | 2 | Sicilia, Palermo, Italy (ITA) | Ant_sic_2C | SAMN17246023 |
| *Antirrhinum siculum* | VAL117810 | 3 | 192PV00 Marruecos VAL117810 (MO) | Ant_sic_3 | SAMN17246024 |
| *Antirrhinum siculum* | 18PJM10 | 4 | Nápoles, Italy (ITA) | Ant_sic_4* | - |
| *Antirrhinum subbaeticum* | MA705104 | 1 | Bogarra, Albacete, Spain (AB) | Ant_sub_1C | SAMN17246026 |
| *Antirrhinum subbaeticum* | MA593205 | 2 | Bogarra, Albacete, Spain (AB) | Ant_sub_2C | SAMN17246027 |
| *Antirrhinum tortuosum* | 164PV06 | 2 | Turkey | Ant_tor_2 | SAMN17246028 |
| *Antirrhinum tortuosum* | 201PV06 | 3 | Marruecos, P.N. Tazzeka, Morrocco (MO) | Ant_tor_3 | SAMN17246029 |
| *Antirrhinum tortuosum* | MA908877 | 4 | Túnez | Ant_tor_4 | SAMN17246030 |
| *Antirrhinum tortuosum* | MA589750 | 5 | Norma, Italy (ITA) | Ant_tor_5C | SAMN17246031 |
| *Antirrhinum tortuosum* | MA938611 | 6 | Dubrovnik, Croatia (CRO) | Ant_tor_6 | SAMN17246032 |
| *Antirrhinum tortuosum* | MA938610 | 7 | Corfu, Greece (GRE) | Ant_tor_7 | SAMN17246033 |
| *Antirrhinum tortuosum* | PI010588 | 8 | Monte Tifata, Capua, Caserta, Italy (ITA) | Ant_tor_8C | SAMN17246034 |
| *Antirrhinum valentinum* | 27PV11(6) | 1 | Barx-Pla de Corrals, umbría del Biuxcarró, Valencia, Spain (VAL) | Ant_val_1C | SAMN17246035 |
| *Antirrhinum valentinum* | 32PV11(3) | 2 | Xeresa, Valencia, Spain (VAL) | Ant_val_2C | SAMN17246036 |
| *Gambelia speciosa* | 100PV99 | 1 | cultivated at Uppsala Botanical Garden | Gam_spe | SAMN17246037 |
| *Howelliella ovata* | 257574GH | 1 | USA | How_ova_1 | SAMN17246038 |
| *Misopates microcarpum* | MA 729114 | 1 | Spain: Albacete, Balsa de Ves | Mis_mic_1 | SAMN17246039 |
| *Misopates orontium* | VAL145155 | 1 | Spain | Mis_oro_1 | SAMN17246040 |
| *Neogaerrhinum filipes* | Thompson 254 | 1 | USA: Nevada, Clark Co., near Goodspring | Neo_fil_1 | SAMN17246041 |
| *Neogaerrhinum strictum* | A257572 | 1 | E26 OYAMA | Neo_str_1 | SAMN17246042 |
| *Pseudomisopates rivas-martinezii* | E Amat | 1 | Spain, Conventos_1 | Pse_riv_1 | SAMN17246043 |
| *Sairocarpus nuttallianus* | A269966 | 1 | USA: California, San Diego Co., Palomar Mt | Sai_nut_1 | SAMN17246044 |
| *Sairocarpus subcordatus* | A269969 | 1 | USA | Sai_sub_1 | SAMN17246045 |

**Table S2.** Significant D-statistic tests given a four-taxon tree (((P1,P2),P3),O). Abbreviation for each individual is described at Table S1. Tests are differentiated according to the three introgression hypotheses tested (MOLLE: hybrid origin for *A. molle*; LAT: hybrid origin for *A. latifolium*; and SIC: hybrid origin for *A. siculum*). D-statistic value (D), standard deviation (std (D)), z-score (Z), proportion of BABA and ABBA, number of loci involved for each test (nloci), number of bootstrap replicates (nboot), significant pattern (pattern), p-value and adjusted p-value through Bonferroni-Holm method are shown.

| Hypothesis | P1 | P2 | P3 | O | D | std(D) | Z | BABA | ABBA | nloci | nboot | pattern | p-value | adjusted p-value |
| --- | --- | --- | --- | --- | --- | --- | --- | --- | --- | --- | --- | --- | --- | --- |
| MOLLE | [Ant_boi_2C] | [Ant_mol_2C] | [Ant_bra_1C] | [How_ova_1] | -0.363 | 0.07 | 5.19 | 207.62 | 97.12 | 778 | 200 | BABA | 0.000000 | 0.003282690598277 |
| MOLLE | [Ant_rup_1C] | [Ant_mol_3C] | [Ant_gro_1C] | [Sai_nut_1] | -0.756 | 0.112 | 6.77 | 27 | 3.75 | 122 | 200 | BABA | 0.000000 | 2.01647475230776E-07 |
| MOLLE | [Ant_con_4C] | [Ant_mol_3C] | [Ant_gro_1C] | [Sai_nut_1] | -0.638 | 0.104 | 6.11 | 35.62 | 7.88 | 167 | 200 | BABA | 0.000000 | 1.55942660460455E-05 |
| MOLLE | [Ant_cha_1C] | [Ant_mol_1] | [Ant_gro_1C] | [Sai_nut_1] | -0.567 | 0.115 | 4.93 | 47 | 13 | 216 | 200 | BABA | 0.000001 | 0.012821241846589 |
| MOLLE | [Ant_rup_1C] | [Ant_mol_3C] | [Ant_gro_1C] | [How_ova_1] | -0.588 | 0.12 | 4.91 | 26 | 6.75 | 118 | 200 | BABA | 0.000001 | 0.014196077371945 |
| MOLLE | [Ant_con_4C] | [Ant_mol_1] | [Ant_gro_1C] | [Sai_nut_1] | -0.483 | 0.1 | 4.85 | 56 | 19.5 | 216 | 200 | BABA | 0.000001 | 0.019241470785527 |
| MOLLE | [Ant_cha_1C] | [Ant_mol_1] | [Ant_gro_1C] | [How_ova_1] | -0.507 | 0.105 | 4.84 | 51.25 | 16.75 | 204 | 200 | BABA | 0.000001 | 0.020234129796702 |
| MOLLE | [Ant_cha_1C] | [Ant_mol_3C] | [Ant_gro_1C] | [How_ova_1] | -0.556 | 0.117 | 4.73 | 40.25 | 11.5 | 158 | 200 | BABA | 0.000002 | 0.034953248237741 |
| MOLLE | [Ant_lin_2C] | [Ant_mol_1] | [Ant_gro_1C] | [Sai_nut_1] | -0.492 | 0.105 | 4.67 | 49.25 | 16.75 | 220 | 200 | BABA | 0.000003 | 0.046860654852082 |
| MOLLE | [Ant_aus_2C] | [Ant_mol_2C] | [Ant_gro_2C] | [Sai_nut_1] | -0.463 | 0.098 | 4.72 | 58.5 | 21.5 | 281 | 200 | BABA | 0.000002 | 0.036713935623022 |
| MOLLE | [Ant_gra_1C] | [Ant_mol_2C] | [Ant_lop_1C] | [How_ova_1] | -0.561 | 0.079 | 7.15 | 127.62 | 35.88 | 415 | 200 | BABA | 0.000000 | 1.35894192251217E-08 |
| MOLLE | [Ant_gra_bra2C] | [Ant_mol_2C] | [Ant_lop_1C] | [How_ova_1] | -0.495 | 0.07 | 7.06 | 158.25 | 53.5 | 558 | 200 | BABA | 0.000000 | 2.60726428001425E-08 |
| MOLLE | [Ant_gra_bra_1] | [Ant_mol_3C] | [Ant_lop_1C] | [Sai_nut_1] | -0.504 | 0.084 | 6.02 | 120.5 | 39.75 | 356 | 200 | BABA | 0.000000 | 2.72980157506882E-05 |
| MOLLE | [Ant_lin_4] | [Ant_mol_2C] | [Ant_lop_1C] | [Sai_nut_1] | -0.447 | 0.079 | 5.66 | 158.25 | 60.5 | 582 | 200 | BABA | 0.000000 | 0.000236747364242 |
| MOLLE | [Ant_gra_4] | [Ant_mol_2C] | [Ant_lop_1C] | [How_ova_1] | -0.495 | 0.088 | 5.65 | 141.62 | 47.88 | 486 | 200 | BABA | 0.000000 | 0.000250924372305 |
| MOLLE | [Ant_cir_2C] | [Ant_mol_2C] | [Ant_lop_1C] | [How_ova_1] | -0.445 | 0.079 | 5.63 | 149 | 57.25 | 555 | 200 | BABA | 0.000000 | 0.000281775764356 |
| MOLLE | [Ant_lin_4] | [Ant_mol_2C] | [Ant_lop_1C] | [How_ova_1] | -0.461 | 0.083 | 5.58 | 153.75 | 56.75 | 552 | 200 | BABA | 0.000000 | 0.000376026755168 |
| MOLLE | [Ant_cir_2C] | [Ant_mol_2C] | [Ant_lop_1C] | [Sai_nut_1] | -0.426 | 0.079 | 5.42 | 170.75 | 68.75 | 582 | 200 | BABA | 0.000000 | 0.000931234928454 |
| MOLLE | [Ant_gra_bra_1] | [Ant_mol_2C] | [Ant_lop_1C] | [Sai_nut_1] | -0.467 | 0.087 | 5.4 | 172.75 | 62.75 | 537 | 200 | BABA | 0.000000 | 0.001041197374273 |
| MOLLE | [Ant_maj_1C] | [Ant_mol_3C] | [Ant_lop_1C] | [Sai_nut_1] | -0.481 | 0.09 | 5.35 | 94.62 | 33.12 | 366 | 200 | BABA | 0.000000 | 0.001373933056199 |
| MOLLE | [Ant_gra_4] | [Ant_mol_2C] | [Ant_lop_1C] | [Sai_nut_1] | -0.443 | 0.084 | 5.29 | 152.75 | 59 | 511 | 200 | BABA | 0.000000 | 0.001910459225975 |
| MOLLE | [Ant_tor_5C] | [Ant_mol_2C] | [Ant_lop_1C] | [Sai_nut_1] | -0.466 | 0.088 | 5.29 | 152.25 | 55.5 | 573 | 200 | BABA | 0.000000 | 0.001910336909615 |
| MOLLE | [Ant_gra_4] | [Ant_mol_1] | [Ant_lop_1C] | [How_ova_1] | -0.486 | 0.094 | 5.19 | 133.75 | 46.25 | 439 | 200 | BABA | 0.000000 | 0.003282900892357 |
| MOLLE | [Ant_gra_4] | [Ant_mol_1] | [Ant_lop_1C] | [Sai_nut_1] | -0.463 | 0.09 | 5.17 | 150.5 | 55.25 | 470 | 200 | BABA | 0.000000 | 0.003653270881995 |
| MOLLE | [Ant_gra_1C] | [Ant_mol_2C] | [Ant_lop_1C] | [Sai_nut_1] | -0.481 | 0.094 | 5.11 | 132 | 46.25 | 441 | 200 | BABA | 0.000000 | 0.005026643947447 |
| MOLLE | [Ant_aus_2C] | [Ant_mol_3C] | [Ant_lop_1C] | [Sai_nut_1] | -0.603 | 0.118 | 5.09 | 63.5 | 15.75 | 204 | 200 | BABA | 0.000000 | 0.005586506953224 |
| MOLLE | [Ant_gra_bra2C] | [Ant_mol_2C] | [Ant_lop_1C] | [Sai_nut_1] | -0.455 | 0.091 | 5 | 172.75 | 64.75 | 583 | 200 | BABA | 0.000001 | 0.0089418091332 |
| MOLLE | [Ant_gra_1C] | [Ant_mol_1] | [Ant_lop_1C] | [How_ova_1] | -0.512 | 0.103 | 4.97 | 104.5 | 33.75 | 392 | 200 | BABA | 0.000001 | 0.010441305013054 |
| MOLLE | [Ant_gra_bra_1] | [Ant_mol_3C] | [Ant_lop_1C] | [Neo_fil_1] | -0.415 | 0.084 | 4.96 | 95.5 | 39.5 | 352 | 200 | BABA | 0.000001 | 0.010992706432326 |
| MOLLE | [Ant_gra_bra2C] | [Ant_mol_2C] | [Ant_lop_1C] | [Gam_spe] | -0.381 | 0.077 | 4.94 | 183 | 82 | 642 | 200 | BABA | 0.000001 | 0.012181652474771 |
| MOLLE | [Ant_aus_2C] | [Ant_mol_2C] | [Ant_lop_1C] | [Sai_nut_1] | -0.624 | 0.127 | 4.92 | 83.25 | 19.25 | 258 | 200 | BABA | 0.000001 | 0.013493108150972 |
| MOLLE | [Ant_cir_2C] | [Ant_mol_3C] | [Ant_lop_1C] | [Sai_nut_1] | -0.461 | 0.094 | 4.92 | 109.75 | 40.5 | 395 | 200 | BABA | 0.000001 | 0.013492242708848 |
| MOLLE | [Ant_lin_4] | [Ant_mol_2C] | [Ant_lop_1C] | [Gam_spe] | -0.399 | 0.081 | 4.92 | 181 | 77.75 | 641 | 200 | BABA | 0.000001 | 0.013490511824601 |
| MOLLE | [Ant_aus_1C] | [Ant_mol_2C] | [Ant_lop_1C] | [Sai_nut_1] | -0.462 | 0.096 | 4.79 | 140.5 | 51.75 | 480 | 200 | BABA | 0.000002 | 0.025981192967894 |
| MOLLE | [Ant_boi_2C] | [Ant_mol_2C] | [Ant_lop_1C] | [How_ova_1] | -0.379 | 0.08 | 4.77 | 157.75 | 71 | 564 | 200 | BABA | 0.000002 | 0.028693186920607 |
| MOLLE | [Ant_aus_2C] | [Ant_mol_1] | [Ant_lop_1C] | [Sai_nut_1] | -0.628 | 0.132 | 4.75 | 76.5 | 17.5 | 246 | 200 | BABA | 0.000002 | 0.031674006340075 |
| MOLLE | [Ant_tor_5C] | [Ant_mol_2C] | [Ant_lop_1C] | [How_ova_1] | -0.421 | 0.089 | 4.74 | 140 | 57 | 550 | 200 | BABA | 0.000002 | 0.033275926684161 |
| MOLLE | [Ant_boi_2C] | [Ant_mol_2C] | [Ant_lop_1C] | [Sai_nut_1] | -0.394 | 0.083 | 4.74 | 173 | 75.25 | 590 | 200 | BABA | 0.000002 | 0.033273789501971 |
| MOLLE | [Ant_lin_4] | [Ant_mol_3C] | [Ant_lop_1C] | [Sai_nut_1] | -0.452 | 0.096 | 4.72 | 114.5 | 43.25 | 402 | 200 | BABA | 0.000002 | 0.036711577176589 |
| MOLLE | [Ant_aus_1C] | [Ant_mol_2C] | [Ant_lop_1C] | [How_ova_1] | -0.424 | 0.09 | 4.71 | 128.5 | 52 | 458 | 200 | BABA | 0.000002 | 0.038554641424468 |
| MOLLE | [Ant_gra_bra_1] | [Ant_mol_2C] | [Ant_lop_1C] | [How_ova_1] | -0.448 | 0.096 | 4.67 | 136.25 | 52 | 513 | 200 | BABA | 0.000003 | 0.046863666849437 |
| MOLLE | [Ant_aus_2C] | [Ant_mol_2C] | [Ant_lop_2] | [Neo_str] | -0.612 | 0.095 | 6.43 | 80 | 19.25 | 218 | 200 | BABA | 0.000000 | 1.99789497204582E-06 |
| MOLLE | [Ant_gra_4] | [Ant_mol_1] | [Ant_lop_2] | [How_ova_1] | -0.49 | 0.077 | 6.4 | 154.62 | 52.88 | 565 | 200 | BABA | 0.000000 | 2.43258155478498E-06 |
| MOLLE | [Ant_boi_2C] | [Ant_mol_1] | [Ant_lop_2] | [Sai_nut_1] | -0.445 | 0.069 | 6.4 | 203.75 | 78.25 | 703 | 200 | BABA | 0.000000 | 2.43242617783335E-06 |
| MOLLE | [Ant_gra_bra_1] | [Ant_mol_1] | [Ant_lop_2] | [Sai_nut_1] | -0.452 | 0.072 | 6.28 | 191.62 | 72.38 | 650 | 200 | BABA | 0.000000 | 5.29968362739201E-06 |
| MOLLE | [Ant_boi_2C] | [Ant_mol_2C] | [Ant_lop_2] | [How_ova_1] | -0.411 | 0.069 | 5.94 | 231 | 96.5 | 776 | 200 | BABA | 0.000000 | 4.46059550065726E-05 |
| MOLLE | [Ant_gra_1C] | [Ant_mol_1] | [Ant_lop_2] | [How_ova_1] | -0.495 | 0.084 | 5.89 | 143 | 48.25 | 503 | 200 | BABA | 0.000000 | 6.04357523918122E-05 |
| MOLLE | [Ant_gra_bra_1] | [Ant_mol_2C] | [Ant_lop_2] | [Sai_nut_1] | -0.376 | 0.064 | 5.86 | 206.25 | 93.5 | 753 | 200 | BABA | 0.000000 | 7.24294548422989E-05 |
| MOLLE | [Ant_boi_2C] | [Ant_mol_1] | [Ant_lop_2] | [How_ova_1] | -0.431 | 0.074 | 5.8 | 196.75 | 78.25 | 664 | 200 | BABA | 0.000000 | 0.000103749691662 |
| MOLLE | [Ant_gra_bra_1] | [Ant_mol_1] | [Ant_lop_2] | [Neo_fil_1] | -0.435 | 0.076 | 5.73 | 188.75 | 74.25 | 656 | 200 | BABA | 0.000000 | 0.000157113684609 |
| MOLLE | [Ant_gra_4] | [Ant_mol_1] | [Ant_lop_2] | [Sai_nut_1] | -0.431 | 0.076 | 5.67 | 180.88 | 71.88 | 601 | 200 | BABA | 0.000000 | 0.000223349600382 |
| MOLLE | [Ant_gra_1C] | [Ant_mol_2C] | [Ant_lop_2] | [How_ova_1] | -0.471 | 0.084 | 5.6 | 180.38 | 64.88 | 536 | 200 | BABA | 0.000000 | 0.000335139047377 |
| MOLLE | [Ant_lin_2C] | [Ant_mol_1] | [Ant_lop_2] | [How_ova_1] | -0.417 | 0.075 | 5.56 | 170 | 70 | 652 | 200 | BABA | 0.000000 | 0.000421738710823 |
| MOLLE | [Ant_lin_2C] | [Ant_mol_2C] | [Ant_lop_2] | [Sai_nut_1] | -0.385 | 0.07 | 5.54 | 229.12 | 101.62 | 793 | 200 | BABA | 0.000000 | 0.000472823678145 |
| MOLLE | [Ant_aus_1C] | [Ant_mol_1] | [Ant_lop_2] | [Sai_nut_1] | -0.489 | 0.088 | 5.53 | 158.75 | 54.5 | 559 | 200 | BABA | 0.000000 | 0.000500552744351 |
| MOLLE | [Ant_lin_2C] | [Ant_mol_2C] | [Ant_lop_2] | [How_ova_1] | -0.404 | 0.073 | 5.52 | 210 | 89.25 | 750 | 200 | BABA | 0.000000 | 0.000529856472849 |
| MOLLE | [Ant_gra_bra_1] | [Ant_mol_1] | [Ant_lop_2] | [Gam_spe] | -0.391 | 0.071 | 5.5 | 216.5 | 94.75 | 720 | 200 | BABA | 0.000000 | 0.000593575743559 |
| MOLLE | [Ant_his_1C] | [Ant_mol_1] | [Ant_lop_2] | [How_ova_1] | -0.398 | 0.072 | 5.48 | 171.38 | 73.88 | 642 | 200 | BABA | 0.000000 | 0.000664699216437 |
| MOLLE | [Ant_lin_2C] | [Ant_mol_1] | [Ant_lop_2] | [Neo_fil_1] | -0.366 | 0.067 | 5.47 | 182.5 | 84.75 | 692 | 200 | BABA | 0.000000 | 0.000703270599249 |
| MOLLE | [Ant_gra_bra2C] | [Ant_mol_1] | [Ant_lop_2] | [How_ova_1] | -0.424 | 0.079 | 5.38 | 177.88 | 71.88 | 659 | 200 | BABA | 0.000000 | 0.001163692258287 |
| MOLLE | [Ant_lin_2C] | [Ant_mol_1] | [Ant_lop_2] | [Sai_nut_1] | -0.408 | 0.076 | 5.35 | 184.12 | 77.38 | 688 | 200 | BABA | 0.000000 | 0.001373845101967 |
| MOLLE | [Ant_his_1C] | [Ant_mol_1] | [Ant_lop_2] | [Sai_nut_1] | -0.395 | 0.075 | 5.28 | 194.62 | 84.38 | 682 | 200 | BABA | 0.000000 | 0.002017464755777 |
| MOLLE | [Ant_gra_bra_1] | [Ant_mol_1] | [Ant_lop_2] | [Mis_oro_1] | -0.391 | 0.074 | 5.27 | 180.25 | 79 | 553 | 200 | BABA | 0.000000 | 0.002130393417791 |
| MOLLE | [Ant_boi_2C] | [Ant_mol_1] | [Ant_lop_2] | [Pse_riv_1] | -0.399 | 0.076 | 5.23 | 209.5 | 90 | 607 | 200 | BABA | 0.000000 | 0.002646729760089 |
| MOLLE | [Ant_gra_4] | [Ant_mol_2C] | [Ant_lop_2] | [How_ova_1] | -0.436 | 0.084 | 5.2 | 188.25 | 74 | 638 | 200 | BABA | 0.000000 | 0.003111491761726 |
| MOLLE | [Ant_lin_2C] | [Ant_mol_2C] | [Ant_lop_2] | [Neo_str] | -0.428 | 0.083 | 5.19 | 150.5 | 60.25 | 479 | 200 | BABA | 0.000000 | 0.003282480304196 |
| MOLLE | [Ant_boi_2C] | [Ant_mol_2C] | [Ant_lop_2] | [Sai_nut_1] | -0.375 | 0.072 | 5.18 | 232.75 | 105.75 | 815 | 200 | BABA | 0.000000 | 0.003463195131144 |
| MOLLE | [Ant_maj_3] | [Ant_mol_1] | [Ant_lop_2] | [How_ova_1] | -0.418 | 0.082 | 5.08 | 165 | 67.75 | 581 | 200 | BABA | 0.000000 | 0.0058883613806 |
| MOLLE | [Ant_gra_bra_1] | [Ant_mol_1] | [Ant_lop_2] | [Sai_sub_1] | -0.385 | 0.076 | 5.05 | 162.38 | 72.12 | 571 | 200 | BABA | 0.000000 | 0.00689223700681 |
| MOLLE | [Ant_aus_1C] | [Ant_mol_1] | [Ant_lop_2] | [Neo_fil_1] | -0.426 | 0.085 | 5.01 | 154.88 | 62.38 | 564 | 200 | BABA | 0.000001 | 0.008489996930601 |
| MOLLE | [Ant_lin_4] | [Ant_mol_2C] | [Ant_lop_2] | [Neo_fil_1] | -0.316 | 0.064 | 4.92 | 215.62 | 112.12 | 791 | 200 | BABA | 0.000001 | 0.013491377266724 |
| MOLLE | [Ant_his_1C] | [Ant_mol_1] | [Ant_lop_2] | [Neo_fil_1] | -0.389 | 0.08 | 4.87 | 200 | 88 | 679 | 200 | BABA | 0.000001 | 0.017393703033335 |
| MOLLE | [Ant_gra_bra_1] | [Ant_mol_2C] | [Ant_lop_2] | [How_ova_1] | -0.358 | 0.074 | 4.84 | 182.62 | 86.38 | 717 | 200 | BABA | 0.000001 | 0.020232831405416 |
| MOLLE | [Ant_onu_3C] | [Ant_mol_1] | [Ant_lop_2] | [How_ova_1] | -0.572 | 0.118 | 4.83 | 86.25 | 23.5 | 306 | 200 | BABA | 0.000001 | 0.021273214600327 |
| MOLLE | [Ant_gra_1C] | [Ant_mol_2C] | [Ant_lop_2] | [Neo_str] | -0.42 | 0.088 | 4.79 | 117 | 47.75 | 366 | 200 | BABA | 0.000002 | 0.025979525154763 |
| MOLLE | [Ant_gra_4] | [Ant_mol_2C] | [Ant_lop_2] | [Pse_riv_1] | -0.342 | 0.071 | 4.78 | 191.5 | 94 | 594 | 200 | BABA | 0.000002 | 0.027303979508691 |
| MOLLE | [Ant_lin_2C] | [Ant_mol_2C] | [Ant_lop_2] | [Neo_fil_1] | -0.35 | 0.073 | 4.77 | 220.38 | 106.12 | 795 | 200 | BABA | 0.000002 | 0.028691344661414 |
| MOLLE | [Ant_gra_4] | [Ant_mol_1] | [Ant_lop_2] | [Gam_spe] | -0.348 | 0.074 | 4.71 | 192.75 | 93.25 | 658 | 200 | BABA | 0.000002 | 0.038552164256553 |
| MOLLE | [Ant_gra_1C] | [Ant_mol_1] | [Ant_lop_2] | [Sai_nut_1] | -0.412 | 0.088 | 4.67 | 149 | 62 | 534 | 200 | BABA | 0.000003 | 0.046857642854727 |
| MOLLE | [Ant_aus_2C] | [Ant_mol_1] | [Ant_lop_2] | [Neo_fil_1] | -0.547 | 0.117 | 4.67 | 79.5 | 23.25 | 288 | 200 | BABA | 0.000003 | 0.046854630857372 |
| MOLLE | [Ant_boi_2C] | [Ant_mol_2C] | [Ant_meo_2C] | [How_ova_1] | -0.347 | 0.067 | 5.14 | 196.12 | 95.12 | 786 | 200 | BABA | 0.000000 | 0.004287293804308 |
| MOLLE | [Ant_lin_2C] | [Ant_mol_2C] | [Ant_meo_2C] | [How_ova_1] | -0.334 | 0.071 | 4.69 | 180.88 | 90.38 | 745 | 200 | BABA | 0.000003 | 0.042510705612143 |
| MOLLE | [Ant_boi_2C] | [Ant_mol_2C] | [Ant_meo_5C] | [Neo_fil_1] | -0.423 | 0.067 | 6.37 | 209.25 | 84.75 | 738 | 200 | BABA | 0.000000 | 2.95904744702503E-06 |
| MOLLE | [Ant_boi_2C] | [Ant_mol_2C] | [Ant_meo_5C] | [Sai_nut_1] | -0.415 | 0.074 | 5.64 | 202.5 | 83.75 | 738 | 200 | BABA | 0.000000 | 0.000265924446947 |
| MOLLE | [Ant_boi_2C] | [Ant_mol_2C] | [Ant_meo_5C] | [How_ova_1] | -0.39 | 0.075 | 5.19 | 195.25 | 85.75 | 710 | 200 | BABA | 0.000000 | 0.003283111186438 |
| MOLLE | [Ant_boi_2C] | [Ant_mol_1] | [Ant_meo_5C] | [Sai_nut_1] | -0.431 | 0.084 | 5.14 | 170.88 | 67.88 | 650 | 200 | BABA | 0.000000 | 0.00428701906584 |
| MOLLE | [Ant_lin_2C] | [Ant_mol_1] | [Ant_meo_5C] | [Gam_spe] | -0.347 | 0.072 | 4.84 | 168.38 | 81.62 | 712 | 200 | BABA | 0.000001 | 0.020231533014131 |
| MOLLE | [Ant_lin_2C] | [Ant_mol_1] | [Ant_meo_5C] | [Sai_nut_1] | -0.409 | 0.086 | 4.76 | 149.38 | 62.62 | 633 | 200 | BABA | 0.000002 | 0.030148231537236 |
| MOLLE | [Ant_boi_2C] | [Ant_mol_2C] | [Ant_meo_5C] | [Gam_spe] | -0.344 | 0.072 | 4.76 | 232.12 | 113.38 | 826 | 200 | BABA | 0.000002 | 0.030146295607644 |
| MOLLE | [Ant_lin_2C] | [Ant_mol_2C] | [Ant_meo_5C] | [Neo_fil_1] | -0.394 | 0.084 | 4.72 | 182.62 | 79.38 | 712 | 200 | BABA | 0.000002 | 0.036709218730156 |
| MOLLE | [Ant_aus_1C] | [Ant_mol_1] | [Ant_meo_5C] | [Sai_nut_1] | -0.457 | 0.097 | 4.7 | 130.38 | 48.62 | 529 | 200 | BABA | 0.000003 | 0.040486331195722 |
| MOLLE | [Ant_onu_3C] | [Ant_mol_1] | [Ant_rot_1C] | [How_ova_1] | -0.689 | 0.118 | 5.85 | 72 | 13.25 | 231 | 200 | BABA | 0.000000 | 7.69164292442211E-05 |
| MOLLE | [Ant_cir_2C] | [Ant_mol_1] | [Ant_rot_1C] | [How_ova_1] | -0.542 | 0.093 | 5.82 | 138 | 41 | 421 | 200 | BABA | 0.000000 | 9.20729978627207E-05 |
| MOLLE | [Ant_lin_2C] | [Ant_mol_1] | [Ant_rot_1C] | [How_ova_1] | -0.503 | 0.088 | 5.71 | 119.5 | 39.5 | 413 | 200 | BABA | 0.000000 | 0.000176728628224 |
| MOLLE | [Ant_gra_1C] | [Ant_mol_1] | [Ant_rot_1C] | [Mis_oro_1] | -0.524 | 0.092 | 5.71 | 109 | 34 | 330 | 200 | BABA | 0.000000 | 0.000176717330607 |
| MOLLE | [Ant_gra_bra_1] | [Ant_mol_1] | [Ant_rot_1C] | [Mis_oro_1] | -0.524 | 0.093 | 5.64 | 125.5 | 39.25 | 360 | 200 | BABA | 0.000000 | 0.000265907441931 |
| MOLLE | [Ant_gra_1C] | [Ant_mol_1] | [Ant_rot_1C] | [How_ova_1] | -0.563 | 0.103 | 5.45 | 93 | 26 | 337 | 200 | BABA | 0.000000 | 0.000787078809022 |
| MOLLE | [Ant_cir_2C] | [Ant_mol_2C] | [Ant_rot_1C] | [How_ova_1] | -0.489 | 0.091 | 5.36 | 132 | 45.25 | 456 | 200 | BABA | 0.000000 | 0.001300093332576 |
| MOLLE | [Ant_lin_2C] | [Ant_mol_2C] | [Ant_rot_1C] | [How_ova_1] | -0.48 | 0.091 | 5.25 | 136.38 | 47.88 | 451 | 200 | BABA | 0.000000 | 0.002375029169299 |
| MOLLE | [Ant_tor_5C] | [Ant_mol_1] | [Ant_rot_1C] | [How_ova_1] | -0.523 | 0.101 | 5.17 | 122.25 | 38.25 | 413 | 200 | BABA | 0.000000 | 0.003653504975989 |
| MOLLE | [Ant_gra_bra2C] | [Ant_mol_1] | [Ant_rot_1C] | [How_ova_1] | -0.495 | 0.099 | 5.02 | 124.5 | 42 | 421 | 200 | BABA | 0.000001 | 0.008060234091572 |
| MOLLE | [Ant_lit_2C] | [Ant_mol_1] | [Ant_rot_1C] | [Sai_nut_1] | -0.512 | 0.102 | 5 | 107 | 34.5 | 385 | 200 | BABA | 0.000001 | 0.008941235830056 |
| MOLLE | [Ant_gra_bra_1] | [Ant_mol_1] | [Ant_rot_1C] | [Sai_sub_1] | -0.502 | 0.104 | 4.81 | 115.25 | 38.25 | 369 | 200 | BABA | 0.000002 | 0.023514940090201 |
| MOLLE | [Ant_gra_1C] | [Ant_mol_2C] | [Ant_rot_1C] | [Mis_oro_1] | -0.486 | 0.101 | 4.81 | 115 | 39.75 | 343 | 200 | BABA | 0.000002 | 0.023513430787243 |
| MOLLE | [Ant_his_1C] | [Ant_mol_1] | [Ant_rot_1C] | [Neo_fil_1] | -0.48 | 0.102 | 4.7 | 131 | 46 | 438 | 200 | BABA | 0.000003 | 0.040483729580814 |
| LAT | [Ant_gra_bra_1] | [Ant_lat_4C] | [Ant_bra_1C] | [Sai_sub_1] | -0.403 | 0.08 | 5.06 | 163.75 | 69.75 | 650 | 200 | BABA | 0.000000 | 0.006518599720515 |
| LAT | [Ant_gra_bra_1] | [Ant_lat_3] | [Ant_bra_1C] | [Sai_nut_1] | -0.349 | 0.075 | 4.67 | 206.12 | 99.38 | 730 | 200 | BABA | 0.000003 | 0.04651126315888 |
| LAT | [Ant_gra_bra_1] | [Ant_lat_1] | [Ant_bra_4] | [Sai_sub_1] | -0.456 | 0.079 | 5.75 | 185.12 | 69.12 | 631 | 200 | BABA | 0.000000 | 0.000139442889184 |
| LAT | [Ant_gra_bra_1] | [Ant_lat_3] | [Ant_bra_4] | [Sai_sub_1] | -0.43 | 0.077 | 5.59 | 174.25 | 69.5 | 653 | 200 | BABA | 0.000000 | 0.000354569199732 |
| LAT | [Ant_gra_bra_1] | [Ant_lat_1] | [Ant_bra_4] | [Gam_spe] | -0.392 | 0.074 | 5.29 | 242.75 | 106 | 795 | 200 | BABA | 0.000000 | 0.001905933520657 |
| LAT | [Ant_gra_bra_1] | [Ant_lat_4C] | [Ant_bra_4] | [Gam_spe] | -0.337 | 0.066 | 5.12 | 235.25 | 116.75 | 818 | 200 | BABA | 0.000000 | 0.004754440352184 |
| LAT | [Ant_gra_bra_1] | [Ant_lat_3] | [Ant_bra_4] | [Gam_spe] | -0.34 | 0.067 | 5.07 | 231.5 | 114 | 821 | 200 | BABA | 0.000000 | 0.006185636353623 |
| LAT | [Ant_gra_bra_1] | [Ant_lat_1] | [Ant_bra_4] | [How_ova_1] | -0.399 | 0.085 | 4.67 | 189.25 | 81.25 | 669 | 200 | BABA | 0.000003 | 0.046526323145656 |
| LAT | [Ant_cir_2C] | [Ant_lat_4C] | [Ant_gro_2C] | [Pse_riv_1] | -0.354 | 0.069 | 5.14 | 149.5 | 71.25 | 566 | 200 | BABA | 0.000000 | 0.004276029527091 |
| LAT | [Ant_cir_2C] | [Ant_lat_1] | [Ant_gro_2C] | [Pse_riv_1] | -0.389 | 0.079 | 4.93 | 157.62 | 69.38 | 554 | 200 | BABA | 0.000001 | 0.012759569634013 |
| LAT | [Ant_gra_bra2C] | [Ant_lat_3] | [Ant_lop_1C] | [Sai_nut_1] | -0.54 | 0.07 | 7.67 | 180 | 53.75 | 560 | 200 | BABA | 0.000000 | 2.69346283799601E-10 |
| LAT | [Ant_aus_2C] | [Ant_lat_1] | [Ant_lop_1C] | [Sai_nut_1] | -0.671 | 0.095 | 7.03 | 90 | 17.75 | 244 | 200 | BABA | 0.000000 | 3.23410867559541E-08 |
| LAT | [Ant_rup_1C] | [Ant_lat_3] | [Ant_lop_1C] | [Sai_nut_1] | -0.542 | 0.078 | 6.97 | 86.75 | 25.75 | 363 | 200 | BABA | 0.000000 | 4.96266195169829E-08 |
| LAT | [Ant_gra_bra_1] | [Ant_lat_3] | [Ant_lop_1C] | [Sai_nut_1] | -0.544 | 0.079 | 6.9 | 176 | 52 | 509 | 200 | BABA | 0.000000 | 8.14203758019936E-08 |
| LAT | [Ant_aus_2C] | [Ant_lat_3] | [Ant_lop_1C] | [Sai_nut_1] | -0.669 | 0.1 | 6.71 | 94.5 | 18.75 | 251 | 200 | BABA | 0.000000 | 3.04703982709539E-07 |
| LAT | [Ant_aus_2C] | [Ant_lat_4C] | [Ant_lop_1C] | [Sai_nut_1] | -0.699 | 0.105 | 6.65 | 86 | 15.25 | 247 | 200 | BABA | 0.000000 | 4.58837122099352E-07 |
| LAT | [Ant_lin_2C] | [Ant_lat_3] | [Ant_lop_1C] | [Sai_nut_1] | -0.499 | 0.075 | 6.63 | 171.5 | 57.25 | 556 | 200 | BABA | 0.000000 | 5.25484226690704E-07 |
| LAT | [Ant_gra_1C] | [Ant_lat_3] | [Ant_lop_1C] | [Sai_nut_1] | -0.537 | 0.081 | 6.63 | 146.25 | 44 | 429 | 200 | BABA | 0.000000 | 5.25450658003679E-07 |
| LAT | [Ant_aus_2C] | [Ant_lat_1] | [Ant_lop_1C] | [Sai_sub_1] | -0.679 | 0.103 | 6.59 | 74.5 | 14.25 | 217 | 200 | BABA | 0.000000 | 6.88416556900529E-07 |
| LAT | [Ant_aus_2C] | [Ant_lat_4C] | [Ant_lop_1C] | [Sai_sub_1] | -0.685 | 0.107 | 6.41 | 70.75 | 13.25 | 219 | 200 | BABA | 0.000000 | 2.27752783010724E-06 |
| LAT | [Ant_gra_bra_1] | [Ant_lat_4C] | [Ant_lop_1C] | [Pse_riv_1] | -0.505 | 0.079 | 6.4 | 157.25 | 51.75 | 440 | 200 | BABA | 0.000000 | 2.43164929307518E-06 |
| LAT | [Ant_tor_5C] | [Ant_lat_3] | [Ant_lop_1C] | [Sai_nut_1] | -0.514 | 0.082 | 6.3 | 166.12 | 53.38 | 548 | 200 | BABA | 0.000000 | 4.65755904282736E-06 |
| LAT | [Ant_lin_4] | [Ant_lat_3] | [Ant_lop_1C] | [Sai_nut_1] | -0.508 | 0.081 | 6.29 | 170.38 | 55.62 | 557 | 200 | BABA | 0.000000 | 4.96739005064626E-06 |
| LAT | [Ant_tor_7] | [Ant_lat_3] | [Ant_lop_1C] | [Sai_nut_1] | -0.502 | 0.08 | 6.24 | 156.38 | 51.88 | 559 | 200 | BABA | 0.000000 | 6.84579663046287E-06 |
| LAT | [Ant_gra_bra2C] | [Ant_lat_4C] | [Ant_lop_1C] | [Sai_nut_1] | -0.508 | 0.083 | 6.15 | 167.25 | 54.5 | 562 | 200 | BABA | 0.000000 | 1.21214322182788E-05 |
| LAT | [Ant_his_1C] | [Ant_lat_3] | [Ant_lop_1C] | [Sai_nut_1] | -0.48 | 0.078 | 6.12 | 168.12 | 59.12 | 548 | 200 | BABA | 0.000000 | 1.46379931711646E-05 |
| LAT | [Ant_aus_2C] | [Ant_lat_3] | [Ant_lop_1C] | [Sai_sub_1] | -0.658 | 0.108 | 6.11 | 72.75 | 15 | 222 | 200 | BABA | 0.000000 | 1.55843029320371E-05 |
| LAT | [Ant_gra_1C] | [Ant_lat_1] | [Ant_lop_1C] | [Sai_nut_1] | -0.521 | 0.087 | 5.99 | 138.25 | 43.5 | 422 | 200 | BABA | 0.000000 | 3.28212366857968E-05 |
| LAT | [Ant_gra_bra2C] | [Ant_lat_4C] | [Ant_lop_1C] | [Pse_riv_1] | -0.512 | 0.085 | 5.99 | 159.5 | 51.5 | 489 | 200 | BABA | 0.000000 | 3.28191382754212E-05 |
| LAT | [Ant_con_4C] | [Ant_lat_3] | [Ant_lop_1C] | [Sai_nut_1] | -0.472 | 0.079 | 5.98 | 168.88 | 60.62 | 560 | 200 | BABA | 0.000000 | 3.48964924361262E-05 |
| LAT | [Ant_gra_bra2C] | [Ant_lat_3] | [Ant_lop_1C] | [Gam_spe] | -0.445 | 0.075 | 5.91 | 179 | 68.75 | 619 | 200 | BABA | 0.000000 | 5.34953859142027E-05 |
| LAT | [Ant_gra_4] | [Ant_lat_3] | [Ant_lop_1C] | [Sai_nut_1] | -0.509 | 0.086 | 5.9 | 158.25 | 51.5 | 490 | 200 | BABA | 0.000000 | 5.68371058948456E-05 |
| LAT | [Ant_gra_bra_1] | [Ant_lat_3] | [Ant_lop_1C] | [Pse_riv_1] | -0.476 | 0.081 | 5.89 | 155.75 | 55.25 | 436 | 200 | BABA | 0.000000 | 6.03778230489866E-05 |
| LAT | [Ant_gra_4] | [Ant_lat_4C] | [Ant_lop_1C] | [Pse_riv_1] | -0.473 | 0.081 | 5.86 | 143.25 | 51.25 | 440 | 200 | BABA | 0.000000 | 7.23553960950164E-05 |
| LAT | [Ant_aus_2C] | [Ant_lat_4C] | [Ant_lop_1C] | [Pse_riv_1] | -0.682 | 0.118 | 5.8 | 75.25 | 14.25 | 225 | 200 | BABA | 0.000000 | 0.000103643587791 |
| LAT | [Ant_gra_bra_1] | [Ant_lat_4C] | [Ant_lop_1C] | [Sai_sub_1] | -0.485 | 0.084 | 5.78 | 123.25 | 42.75 | 444 | 200 | BABA | 0.000000 | 0.000116742137383 |
| LAT | [Ant_gra_4] | [Ant_lat_3] | [Ant_lop_1C] | [How_ova_1] | -0.485 | 0.084 | 5.77 | 141.62 | 49.12 | 464 | 200 | BABA | 0.000000 | 0.000123877623888 |
| LAT | [Ant_lin_2C] | [Ant_lat_1] | [Ant_lop_1C] | [Sai_nut_1] | -0.486 | 0.085 | 5.73 | 162.5 | 56.25 | 549 | 200 | BABA | 0.000000 | 0.00015691282334 |
| LAT | [Ant_lin_4] | [Ant_lat_3] | [Ant_lop_1C] | [Gam_spe] | -0.427 | 0.075 | 5.68 | 192.12 | 77.12 | 619 | 200 | BABA | 0.000000 | 0.000210420126001 |
| LAT | [Ant_gra_bra2C] | [Ant_lat_3] | [Ant_lop_1C] | [How_ova_1] | -0.484 | 0.085 | 5.67 | 156.75 | 54.5 | 535 | 200 | BABA | 0.000000 | 0.000223064005343 |
| LAT | [Ant_aus_1C] | [Ant_lat_3] | [Ant_lop_1C] | [Sai_nut_1] | -0.481 | 0.085 | 5.65 | 143.5 | 50.25 | 465 | 200 | BABA | 0.000000 | 0.000250603476631 |
| LAT | [Ant_cir_2C] | [Ant_lat_3] | [Ant_lop_1C] | [Sai_nut_1] | -0.493 | 0.088 | 5.61 | 162.75 | 55.25 | 561 | 200 | BABA | 0.000000 | 0.000315973474819 |
| LAT | [Ant_maj_3] | [Ant_lat_3] | [Ant_lop_1C] | [Sai_nut_1] | -0.478 | 0.086 | 5.57 | 141.75 | 50 | 500 | 200 | BABA | 0.000000 | 0.000397699050467 |
| LAT | [Ant_gra_bra_1] | [Ant_lat_4C] | [Ant_lop_1C] | [Sai_nut_1] | -0.473 | 0.085 | 5.56 | 157.25 | 56.25 | 510 | 200 | BABA | 0.000000 | 0.000421145206592 |
| LAT | [Ant_con_1C] | [Ant_lat_3] | [Ant_lop_1C] | [Sai_nut_1] | -0.46 | 0.083 | 5.55 | 172.5 | 63.75 | 559 | 200 | BABA | 0.000000 | 0.000445930242288 |
| LAT | [Ant_maj_3] | [Ant_lat_3] | [Ant_lop_1C] | [Gam_spe] | -0.455 | 0.082 | 5.53 | 146.25 | 54.75 | 545 | 200 | BABA | 0.000000 | 0.00049978419046 |
| LAT | [Ant_aus_2C] | [Ant_lat_1] | [Ant_lop_1C] | [Pse_riv_1] | -0.646 | 0.117 | 5.53 | 74.5 | 16 | 222 | 200 | BABA | 0.000000 | 0.000499752167381 |
| LAT | [Ant_gra_4] | [Ant_lat_3] | [Ant_lop_1C] | [Pse_riv_1] | -0.461 | 0.083 | 5.53 | 147.38 | 54.38 | 434 | 200 | BABA | 0.000000 | 0.000499720144302 |
| LAT | [Ant_gra_bra2C] | [Ant_lat_3] | [Ant_lop_1C] | [Pse_riv_1] | -0.461 | 0.084 | 5.48 | 158.75 | 58.5 | 482 | 200 | BABA | 0.000000 | 0.000663423238929 |
| LAT | [Ant_gra_bra_1] | [Ant_lat_3] | [Ant_lop_1C] | [How_ova_1] | -0.453 | 0.083 | 5.44 | 140.75 | 53 | 486 | 200 | BABA | 0.000000 | 0.000831017069317 |
| LAT | [Ant_lin_4] | [Ant_lat_4C] | [Ant_lop_1C] | [Sai_nut_1] | -0.469 | 0.086 | 5.44 | 164.75 | 59.5 | 559 | 200 | BABA | 0.000000 | 0.000830963788746 |
| LAT | [Ant_lin_4] | [Ant_lat_3] | [Ant_lop_1C] | [How_ova_1] | -0.451 | 0.083 | 5.42 | 155.62 | 58.88 | 530 | 200 | BABA | 0.000000 | 0.000929446957392 |
| LAT | [Ant_gra_1C] | [Ant_lat_3] | [Ant_lop_1C] | [How_ova_1] | -0.512 | 0.096 | 5.34 | 125.12 | 40.38 | 400 | 200 | BABA | 0.000000 | 0.001449037205692 |
| LAT | [Ant_gra_4] | [Ant_lat_1] | [Ant_lop_1C] | [Sai_nut_1] | -0.481 | 0.091 | 5.29 | 149 | 52.25 | 484 | 200 | BABA | 0.000000 | 0.001906055837017 |
| LAT | [Ant_cir_2C] | [Ant_lat_4C] | [Ant_lop_1C] | [Pse_riv_1] | -0.444 | 0.085 | 5.24 | 145.5 | 56 | 493 | 200 | BABA | 0.000000 | 0.002501622808245 |
| LAT | [Ant_tor_7] | [Ant_lat_4C] | [Ant_lop_1C] | [Sai_nut_1] | -0.463 | 0.089 | 5.23 | 139.88 | 51.38 | 560 | 200 | BABA | 0.000000 | 0.002640627398659 |
| LAT | [Ant_lit_2C] | [Ant_lat_3] | [Ant_lop_1C] | [Gam_spe] | -0.43 | 0.084 | 5.15 | 155 | 61.75 | 489 | 200 | BABA | 0.000000 | 0.004055774167664 |
| LAT | [Ant_aus_1C] | [Ant_lat_4C] | [Ant_lop_1C] | [Pse_riv_1] | -0.465 | 0.09 | 5.15 | 135.38 | 49.38 | 422 | 200 | BABA | 0.000000 | 0.004055253194746 |
| LAT | [Ant_lin_2C] | [Ant_lat_4C] | [Ant_lop_1C] | [Sai_nut_1] | -0.446 | 0.087 | 5.14 | 161 | 61.75 | 560 | 200 | BABA | 0.000000 | 0.004276579004028 |
| LAT | [Ant_boi_2C] | [Ant_lat_3] | [Ant_lop_1C] | [Sai_nut_1] | -0.435 | 0.085 | 5.12 | 171 | 67.25 | 563 | 200 | BABA | 0.000000 | 0.00475474588784 |
| LAT | [Ant_cir_2C] | [Ant_lat_3] | [Ant_lop_1C] | [How_ova_1] | -0.46 | 0.09 | 5.11 | 137.25 | 50.75 | 534 | 200 | BABA | 0.000000 | 0.005012791118521 |
| LAT | [Ant_cir_2C] | [Ant_lat_3] | [Ant_lop_1C] | [Gam_spe] | -0.424 | 0.083 | 5.11 | 172 | 69.5 | 620 | 200 | BABA | 0.000000 | 0.00501118032446 |
| LAT | [Ant_gra_1C] | [Ant_lat_1] | [Ant_lop_1C] | [Pse_riv_1] | -0.452 | 0.088 | 5.11 | 131 | 49.5 | 370 | 200 | BABA | 0.000000 | 0.005010858165647 |
| LAT | [Ant_lin_2C] | [Ant_lat_3] | [Ant_lop_1C] | [Gam_spe] | -0.397 | 0.078 | 5.1 | 172.75 | 74.5 | 622 | 200 | BABA | 0.000000 | 0.005282630596673 |
| LAT | [Ant_tor_5C] | [Ant_lat_3] | [Ant_lop_1C] | [Gam_spe] | -0.415 | 0.082 | 5.07 | 175.12 | 72.38 | 605 | 200 | BABA | 0.000000 | 0.006186431985028 |
| LAT | [Ant_aus_2C] | [Ant_lat_4C] | [Ant_lop_1C] | [Gam_spe] | -0.556 | 0.11 | 5.07 | 77 | 22 | 279 | 200 | BABA | 0.000000 | 0.006186034169325 |
| LAT | [Ant_his_2C] | [Ant_lat_3] | [Ant_lop_1C] | [Sai_nut_1] | -0.404 | 0.08 | 5.04 | 147.38 | 62.62 | 562 | 200 | BABA | 0.000000 | 0.007236692547581 |
| LAT | [Ant_gra_bra_1] | [Ant_lat_3] | [Ant_lop_1C] | [Gam_spe] | -0.421 | 0.084 | 5.03 | 170.5 | 69.5 | 566 | 200 | BABA | 0.000000 | 0.007622547133308 |
| LAT | [Ant_gra_4] | [Ant_lat_1] | [Ant_lop_1C] | [Pse_riv_1] | -0.465 | 0.092 | 5.03 | 145.25 | 53 | 434 | 200 | BABA | 0.000000 | 0.007622056653472 |
| LAT | [Ant_gra_1C] | [Ant_lat_4C] | [Ant_lop_1C] | [Mis_oro_1] | -0.466 | 0.093 | 5.01 | 114 | 41.5 | 365 | 200 | BABA | 0.000001 | 0.008455706008263 |
| LAT | [Ant_con_1C] | [Ant_lat_4C] | [Ant_lop_1C] | [Sai_nut_1] | -0.41 | 0.082 | 5 | 155.25 | 65 | 559 | 200 | BABA | 0.000001 | 0.008905117731999 |
| LAT | [Ant_aus_1C] | [Ant_lat_4C] | [Ant_lop_1C] | [Sai_sub_1] | -0.447 | 0.09 | 4.99 | 127 | 48.5 | 409 | 200 | BABA | 0.000001 | 0.009377507918823 |
| LAT | [Ant_maj_1C] | [Ant_lat_3] | [Ant_lop_1C] | [Sai_nut_1] | -0.471 | 0.095 | 4.98 | 138.12 | 49.62 | 493 | 200 | BABA | 0.000001 | 0.009874001790982 |
| LAT | [Ant_gra_bra2C] | [Ant_lat_4C] | [Ant_lop_1C] | [How_ova_1] | -0.475 | 0.096 | 4.97 | 141.25 | 50.25 | 538 | 200 | BABA | 0.000001 | 0.010396446568946 |
| LAT | [Ant_lin_4] | [Ant_lat_4C] | [Ant_lop_1C] | [Sai_sub_1] | -0.406 | 0.082 | 4.96 | 140.75 | 59.5 | 494 | 200 | BABA | 0.000001 | 0.010944771070174 |
| LAT | [Ant_con_4C] | [Ant_lat_4C] | [Ant_lop_1C] | [Sai_sub_1] | -0.43 | 0.087 | 4.93 | 142.5 | 56.75 | 494 | 200 | BABA | 0.000001 | 0.012761214226348 |
| LAT | [Ant_gra_1C] | [Ant_lat_3] | [Ant_lop_1C] | [Mis_oro_1] | -0.468 | 0.095 | 4.93 | 118.75 | 43 | 363 | 200 | BABA | 0.000001 | 0.01276039193018 |
| LAT | [Ant_maj_3] | [Ant_lat_3] | [Ant_lop_1C] | [How_ova_1] | -0.484 | 0.098 | 4.92 | 132.25 | 46 | 472 | 200 | BABA | 0.000001 | 0.013428199991692 |
| LAT | [Ant_str_1C] | [Ant_lat_3] | [Ant_lop_1C] | [Sai_nut_1] | -0.453 | 0.092 | 4.92 | 136.38 | 51.38 | 521 | 200 | BABA | 0.000001 | 0.013425603665321 |
| LAT | [Ant_gra_bra2C] | [Ant_lat_1] | [Ant_lop_1C] | [Sai_nut_1] | -0.471 | 0.096 | 4.9 | 170.5 | 61.25 | 553 | 200 | BABA | 0.000001 | 0.014863306873279 |
| LAT | [Ant_tor_5C] | [Ant_lat_1] | [Ant_lop_1C] | [Sai_nut_1] | -0.47 | 0.096 | 4.9 | 160.75 | 58 | 540 | 200 | BABA | 0.000001 | 0.014862348506725 |
| LAT | [Ant_boi_2C] | [Ant_lat_4C] | [Ant_lop_1C] | [Pse_riv_1] | -0.406 | 0.083 | 4.9 | 161 | 68 | 496 | 200 | BABA | 0.000001 | 0.014860431773619 |
| LAT | [Ant_gra_1C] | [Ant_lat_4C] | [Ant_lop_1C] | [Sai_nut_1] | -0.465 | 0.095 | 4.88 | 129.5 | 47.25 | 429 | 200 | BABA | 0.000001 | 0.016442244433822 |
| LAT | [Ant_gra_4] | [Ant_lat_3] | [Ant_lop_1C] | [Gam_spe] | -0.416 | 0.085 | 4.88 | 163.38 | 67.38 | 539 | 200 | BABA | 0.000001 | 0.016441183575416 |
| LAT | [Ant_gra_1C] | [Ant_lat_3] | [Ant_lop_1C] | [Gam_spe] | -0.431 | 0.088 | 4.87 | 137.38 | 54.62 | 471 | 200 | BABA | 0.000001 | 0.017293264609558 |
| LAT | [Ant_gra_1C] | [Ant_lat_1] | [Ant_lop_1C] | [Neo_fil_1] | -0.43 | 0.09 | 4.78 | 125.5 | 50 | 419 | 200 | BABA | 0.000002 | 0.027135696121888 |
| LAT | [Ant_boi_2C] | [Ant_lat_4C] | [Ant_lop_1C] | [Mis_mic_1] | -0.375 | 0.079 | 4.78 | 153.5 | 69.75 | 511 | 200 | BABA | 0.000002 | 0.027132190217997 |
| LAT | [Ant_rup_1C] | [Ant_lat_4C] | [Ant_lop_1C] | [Sai_nut_1] | -0.465 | 0.097 | 4.77 | 82.75 | 30.25 | 362 | 200 | BABA | 0.000002 | 0.028510803260566 |
| LAT | [Ant_cir_2C] | [Ant_lat_3] | [Ant_lop_1C] | [Pse_riv_1] | -0.412 | 0.086 | 4.77 | 141 | 58.75 | 484 | 200 | BABA | 0.000002 | 0.028503434223796 |
| LAT | [Ant_gra_1C] | [Ant_lat_4C] | [Ant_lop_1C] | [Pse_riv_1] | -0.444 | 0.093 | 4.77 | 124 | 47.75 | 374 | 200 | BABA | 0.000002 | 0.028501591964604 |
| LAT | [Ant_gra_bra2C] | [Ant_lat_3] | [Ant_lop_1C] | [Neo_fil_1] | -0.42 | 0.088 | 4.76 | 154.25 | 63 | 553 | 200 | BABA | 0.000002 | 0.029948830789253 |
| LAT | [Ant_gra_bra2C] | [Ant_lat_4C] | [Ant_lop_1C] | [Mis_mic_1] | -0.444 | 0.093 | 4.76 | 136.5 | 52.5 | 502 | 200 | BABA | 0.000002 | 0.029946894859661 |
| LAT | [Ant_gra_4] | [Ant_lat_4C] | [Ant_lop_1C] | [Sai_nut_1] | -0.428 | 0.09 | 4.75 | 146.5 | 58.75 | 492 | 200 | BABA | 0.000002 | 0.03146245302562 |
| LAT | [Ant_tor_5C] | [Ant_lat_4C] | [Ant_lop_1C] | [Sai_nut_1] | -0.49 | 0.103 | 4.75 | 153.25 | 52.5 | 551 | 200 | BABA | 0.000002 | 0.031460418859135 |
| LAT | [Ant_lin_4] | [Ant_lat_4C] | [Ant_lop_1C] | [How_ova_1] | -0.424 | 0.089 | 4.74 | 151.25 | 61.25 | 532 | 200 | BABA | 0.000002 | 0.033049385372117 |
| LAT | [Ant_cir_2C] | [Ant_lat_4C] | [Ant_lop_1C] | [Sai_nut_1] | -0.448 | 0.095 | 4.73 | 153.5 | 58.5 | 563 | 200 | BABA | 0.000002 | 0.034710766813687 |
| LAT | [Ant_aus_2C] | [Ant_lat_1] | [Ant_lop_1C] | [Gam_spe] | -0.547 | 0.115 | 4.73 | 81 | 23.75 | 276 | 200 | BABA | 0.000002 | 0.034708521615316 |
| LAT | [Ant_lin_2C] | [Ant_lat_1] | [Ant_lop_1C] | [Gam_spe] | -0.38 | 0.08 | 4.72 | 171.75 | 77.25 | 615 | 200 | BABA | 0.000002 | 0.036454506515388 |
| LAT | [Ant_lit_2C] | [Ant_lat_3] | [Ant_lop_1C] | [Sai_nut_1] | -0.468 | 0.1 | 4.7 | 137.25 | 49.75 | 447 | 200 | BABA | 0.000003 | 0.040202755170768 |
| LAT | [Ant_aus_1C] | [Ant_lat_4C] | [Ant_lop_1C] | [Sai_nut_1] | -0.449 | 0.096 | 4.69 | 139.12 | 52.88 | 464 | 200 | BABA | 0.000003 | 0.042212912108819 |
| LAT | [Ant_gra_1C] | [Ant_lat_3] | [Ant_lop_1C] | [Sai_sub_1] | -0.472 | 0.101 | 4.67 | 121.62 | 43.62 | 371 | 200 | BABA | 0.000003 | 0.046523311148301 |
| LAT | [Ant_gra_bra2C] | [Ant_lat_3] | [Ant_lop_1C] | [Mis_mic_1] | -0.392 | 0.084 | 4.66 | 139.75 | 61 | 495 | 200 | BABA | 0.000003 | 0.04880691838862 |
| LAT | [Ant_aus_2C] | [Ant_lat_4C] | [Ant_lop_2] | [Neo_str] | -0.559 | 0.095 | 5.9 | 87.5 | 24.75 | 221 | 200 | BABA | 0.000000 | 5.68334708791193E-05 |
| LAT | [Ant_lin_2C] | [Ant_lat_4C] | [Ant_lop_2] | [Sai_nut_1] | -0.381 | 0.065 | 5.84 | 221 | 99 | 770 | 200 | BABA | 0.000000 | 8.15951052437484E-05 |
| LAT | [Ant_gra_bra_1] | [Ant_lat_3] | [Ant_lop_2] | [Sai_nut_1] | -0.41 | 0.07 | 5.83 | 210.25 | 88 | 723 | 200 | BABA | 0.000000 | 8.66329885033986E-05 |
| LAT | [Ant_aus_2C] | [Ant_lat_4C] | [Ant_lop_2] | [Gam_spe] | -0.523 | 0.093 | 5.6 | 115 | 36 | 334 | 200 | BABA | 0.000000 | 0.000334731778948 |
| LAT | [Ant_lin_2C] | [Ant_lat_3] | [Ant_lop_2] | [Sai_nut_1] | -0.395 | 0.071 | 5.58 | 224.5 | 97.25 | 763 | 200 | BABA | 0.000000 | 0.000375521666141 |
| LAT | [Ant_aus_2C] | [Ant_lat_3] | [Ant_lop_2] | [Sai_nut_1] | -0.499 | 0.091 | 5.48 | 110 | 36.75 | 304 | 200 | BABA | 0.000000 | 0.000663508304096 |
| LAT | [Ant_boi_2C] | [Ant_lat_4C] | [Ant_lop_2] | [Sai_nut_1] | -0.37 | 0.068 | 5.48 | 209 | 96 | 782 | 200 | BABA | 0.000000 | 0.000663465771513 |
| LAT | [Ant_boi_2C] | [Ant_lat_3] | [Ant_lop_2] | [Sai_nut_1] | -0.381 | 0.071 | 5.37 | 218.88 | 98.12 | 779 | 200 | BABA | 0.000000 | 0.001227740460913 |
| LAT | [Ant_aus_2C] | [Ant_lat_1] | [Ant_lop_2] | [Neo_str] | -0.538 | 0.1 | 5.36 | 89 | 26.75 | 219 | 200 | BABA | 0.000000 | 0.001297596674019 |
| LAT | [Ant_gra_1C] | [Ant_lat_3] | [Ant_lop_2] | [How_ova_1] | -0.45 | 0.084 | 5.35 | 153.12 | 58.12 | 515 | 200 | BABA | 0.000000 | 0.001371294429243 |
| LAT | [Ant_lin_2C] | [Ant_lat_4C] | [Ant_lop_2] | [Neo_str] | -0.444 | 0.083 | 5.33 | 150.75 | 58 | 475 | 200 | BABA | 0.000000 | 0.001530940606736 |
| LAT | [Ant_cir_2C] | [Ant_lat_3] | [Ant_lop_2] | [Sai_nut_1] | -0.413 | 0.078 | 5.32 | 213.12 | 88.62 | 774 | 200 | BABA | 0.000000 | 0.001617420157498 |
| LAT | [Ant_lin_2C] | [Ant_lat_4C] | [Ant_lop_2] | [Gam_spe] | -0.343 | 0.064 | 5.32 | 256.25 | 125.25 | 870 | 200 | BABA | 0.000000 | 0.001617316390246 |
| LAT | [Ant_boi_2C] | [Ant_lat_1] | [Ant_lop_2] | [Sai_nut_1] | -0.383 | 0.074 | 5.21 | 209.38 | 93.38 | 768 | 200 | BABA | 0.000000 | 0.002941381675447 |
| LAT | [Ant_aus_2C] | [Ant_lat_4C] | [Ant_lop_2] | [Sai_sub_1] | -0.511 | 0.099 | 5.17 | 94.25 | 30.5 | 263 | 200 | BABA | 0.000000 | 0.003645545780168 |
| LAT | [Ant_gra_1C] | [Ant_lat_1] | [Ant_lop_2] | [Sai_nut_1] | -0.421 | 0.082 | 5.16 | 171.75 | 70 | 545 | 200 | BABA | 0.000000 | 0.00384550294699 |
| LAT | [Ant_aus_2C] | [Ant_lat_1] | [Ant_lop_2] | [Sai_sub_1] | -0.523 | 0.102 | 5.14 | 95 | 29.75 | 261 | 200 | BABA | 0.000000 | 0.004276853742497 |
| LAT | [Ant_gra_bra_1] | [Ant_lat_1] | [Ant_lop_2] | [Sai_nut_1] | -0.386 | 0.075 | 5.14 | 207.25 | 91.75 | 720 | 200 | BABA | 0.000000 | 0.00427630426556 |
| LAT | [Ant_gra_bra_1] | [Ant_lat_1] | [Ant_lop_2] | [Sai_sub_1] | -0.417 | 0.082 | 5.11 | 180.25 | 74.25 | 642 | 200 | BABA | 0.000000 | 0.005012468959709 |
| LAT | [Ant_aus_1C] | [Ant_lat_4C] | [Ant_lop_2] | [Sai_nut_1] | -0.397 | 0.078 | 5.11 | 163.12 | 70.38 | 598 | 200 | BABA | 0.000000 | 0.005011502483272 |
| LAT | [Ant_gra_1C] | [Ant_lat_3] | [Ant_lop_2] | [Sai_nut_1] | -0.439 | 0.087 | 5.06 | 175.25 | 68.25 | 549 | 200 | BABA | 0.000000 | 0.006518180464037 |
| LAT | [Ant_lin_2C] | [Ant_lat_1] | [Ant_lop_2] | [Gam_spe] | -0.324 | 0.064 | 5.04 | 248.62 | 126.88 | 857 | 200 | BABA | 0.000000 | 0.007236227015735 |
| LAT | [Ant_lin_2C] | [Ant_lat_1] | [Ant_lop_2] | [Sai_nut_1] | -0.367 | 0.073 | 5.01 | 219.25 | 101.5 | 758 | 200 | BABA | 0.000001 | 0.008456250308618 |
| LAT | [Ant_gra_bra_1] | [Ant_lat_3] | [Ant_lop_2] | [Sai_sub_1] | -0.404 | 0.081 | 5 | 175.5 | 74.5 | 646 | 200 | BABA | 0.000001 | 0.008905691035143 |
| LAT | [Ant_gra_bra_1] | [Ant_lat_1] | [Ant_lop_2] | [Gam_spe] | -0.376 | 0.075 | 4.99 | 244.88 | 111.12 | 816 | 200 | BABA | 0.000001 | 0.009376904125898 |
| LAT | [Ant_aus_2C] | [Ant_lat_1] | [Ant_lop_2] | [Sai_nut_1] | -0.53 | 0.107 | 4.97 | 105 | 32.25 | 297 | 200 | BABA | 0.000001 | 0.010395777039929 |
| LAT | [Ant_his_1C] | [Ant_lat_1] | [Ant_lop_2] | [Sai_nut_1] | -0.363 | 0.073 | 4.96 | 208.38 | 97.38 | 743 | 200 | BABA | 0.000001 | 0.010944066138378 |
| LAT | [Ant_maj_3] | [Ant_lat_4C] | [Ant_lop_2] | [How_ova_1] | -0.414 | 0.084 | 4.94 | 151.88 | 62.88 | 632 | 200 | BABA | 0.000001 | 0.01212540422375 |
| LAT | [Ant_gra_bra_1] | [Ant_lat_4C] | [Ant_lop_2] | [Sai_sub_1] | -0.417 | 0.084 | 4.94 | 166.88 | 68.62 | 647 | 200 | BABA | 0.000001 | 0.012124622998041 |
| LAT | [Ant_lin_4] | [Ant_lat_1] | [Ant_lop_2] | [Gam_spe] | -0.336 | 0.068 | 4.92 | 260.88 | 129.62 | 853 | 200 | BABA | 0.000001 | 0.013424738223198 |
| LAT | [Ant_gra_bra_1] | [Ant_lat_4C] | [Ant_lop_2] | [Gam_spe] | -0.349 | 0.071 | 4.92 | 235.5 | 113.75 | 825 | 200 | BABA | 0.000001 | 0.013423872781074 |
| LAT | [Ant_gra_1C] | [Ant_lat_3] | [Ant_lop_2] | [Sai_sub_1] | -0.423 | 0.086 | 4.9 | 154 | 62.5 | 482 | 200 | BABA | 0.000001 | 0.014864265239832 |
| LAT | [Ant_lin_2C] | [Ant_lat_3] | [Ant_lop_2] | [Pse_riv_1] | -0.355 | 0.073 | 4.89 | 197.12 | 93.88 | 664 | 200 | BABA | 0.000001 | 0.015632601618414 |
| LAT | [Ant_aus_2C] | [Ant_lat_1] | [Ant_lop_2] | [Gam_spe] | -0.532 | 0.11 | 4.86 | 119.5 | 36.5 | 331 | 200 | BABA | 0.000001 | 0.018188922410821 |
| LAT | [Ant_gra_bra_1] | [Ant_lat_4C] | [Ant_lop_2] | [Sai_nut_1] | -0.376 | 0.078 | 4.84 | 195.62 | 88.62 | 729 | 200 | BABA | 0.000001 | 0.02011337940713 |
| LAT | [Ant_gra_bra_1] | [Ant_lat_1] | [Ant_lop_2] | [Neo_fil_1] | -0.38 | 0.079 | 4.84 | 214.12 | 96.12 | 733 | 200 | BABA | 0.000001 | 0.020112081015844 |
| LAT | [Ant_maj_3] | [Ant_lat_3] | [Ant_lop_2] | [How_ova_1] | -0.414 | 0.086 | 4.83 | 152.5 | 63.25 | 626 | 200 | BABA | 0.000001 | 0.02114623886335 |
| LAT | [Ant_lin_2C] | [Ant_lat_3] | [Ant_lop_2] | [Gam_spe] | -0.326 | 0.068 | 4.83 | 246.5 | 125.25 | 864 | 200 | BABA | 0.000001 | 0.021142142871835 |
| LAT | [Ant_his_1C] | [Ant_lat_3] | [Ant_lop_2] | [Sai_nut_1] | -0.338 | 0.07 | 4.81 | 209 | 103.5 | 747 | 200 | BABA | 0.000002 | 0.023367028400288 |
| LAT | [Ant_aus_2C] | [Ant_lat_3] | [Ant_lop_2] | [Gam_spe] | -0.503 | 0.105 | 4.78 | 110.5 | 36.5 | 338 | 200 | BABA | 0.000002 | 0.027133943169942 |
| LAT | [Ant_cir_2C] | [Ant_lat_1] | [Ant_lop_2] | [Sai_nut_1] | -0.378 | 0.079 | 4.77 | 205.12 | 92.62 | 768 | 200 | BABA | 0.000002 | 0.028508961001373 |
| LAT | [Ant_gra_1C] | [Ant_lat_4C] | [Ant_lop_2] | [Sai_nut_1] | -0.401 | 0.084 | 4.77 | 165.88 | 70.88 | 554 | 200 | BABA | 0.000002 | 0.028507118742181 |
| LAT | [Ant_gra_1C] | [Ant_lat_3] | [Ant_lop_2] | [Gam_spe] | -0.394 | 0.083 | 4.75 | 184.88 | 80.38 | 616 | 200 | BABA | 0.000002 | 0.03145838469265 |
| LAT | [Ant_aus_1C] | [Ant_lat_1] | [Ant_lop_2] | [Sai_nut_1] | -0.417 | 0.088 | 4.74 | 165.25 | 68 | 593 | 200 | BABA | 0.000002 | 0.033047248189928 |
| LAT | [Ant_gra_bra_1] | [Ant_lat_1] | [Ant_lop_2] | [Pse_riv_1] | -0.361 | 0.077 | 4.72 | 189.88 | 89.12 | 620 | 200 | BABA | 0.000002 | 0.036452148068955 |
| LAT | [Ant_gra_1C] | [Ant_lat_1] | [Ant_lop_2] | [How_ova_1] | -0.408 | 0.087 | 4.71 | 152.75 | 64.25 | 512 | 200 | BABA | 0.000002 | 0.038284630121765 |
| LAT | [Ant_gra_1C] | [Ant_lat_1] | [Ant_lop_2] | [Gam_spe] | -0.392 | 0.083 | 4.69 | 182.12 | 79.62 | 608 | 200 | BABA | 0.000003 | 0.04221018005833 |
| LAT | [Ant_lin_2C] | [Ant_lat_4C] | [Ant_lop_2] | [Sai_sub_1] | -0.364 | 0.078 | 4.67 | 181.88 | 84.88 | 685 | 200 | BABA | 0.000003 | 0.046520299150945 |
| LAT | [Ant_lin_4] | [Ant_lat_1] | [Ant_lop_2] | [Sai_nut_1] | -0.36 | 0.077 | 4.67 | 201.25 | 94.75 | 751 | 200 | BABA | 0.000003 | 0.04651728715359 |
| LAT | [Ant_tor_5C] | [Ant_lat_3] | [Ant_lop_2] | [Sai_nut_1] | -0.347 | 0.074 | 4.67 | 195.38 | 94.62 | 753 | 200 | BABA | 0.000003 | 0.046514275156235 |
| LAT | [Ant_lin_2C] | [Ant_lat_3] | [Ant_lop_2] | [Neo_str] | -0.411 | 0.088 | 4.67 | 147.62 | 61.62 | 471 | 200 | BABA | 0.000003 | 0.046508251161525 |
| LAT | [Ant_cir_2C] | [Ant_lat_3] | [Ant_lop_2] | [Neo_fil_1] | -0.356 | 0.076 | 4.67 | 211.88 | 100.62 | 786 | 200 | BABA | 0.000003 | 0.046505239164169 |
| LAT | [Ant_cir_2C] | [Ant_lat_1] | [Ant_lop_2] | [Neo_fil_1] | -0.35 | 0.075 | 4.66 | 214.25 | 103.25 | 778 | 200 | BABA | 0.000003 | 0.048813242576296 |
| LAT | [Ant_boi_2C] | [Ant_lat_1] | [Ant_lop_2] | [Pse_riv_1] | -0.364 | 0.078 | 4.66 | 209 | 97.5 | 675 | 200 | BABA | 0.000003 | 0.048803756294782 |
| LAT | [Ant_aus_2C] | [Ant_lat_4C] | [Ant_meo_2C] | [Sai_sub_1] | -0.54 | 0.098 | 5.5 | 70.25 | 21 | 266 | 200 | BABA | 0.000000 | 0.00059258828631 |
| LAT | [Ant_aus_2C] | [Ant_lat_1] | [Ant_meo_2C] | [Sai_sub_1] | -0.504 | 0.1 | 5.03 | 69 | 22.75 | 261 | 200 | BABA | 0.000000 | 0.00762352809298 |
| LAT | [Ant_aus_2C] | [Ant_lat_4C] | [Ant_meo_5C] | [Sai_sub_1] | -0.637 | 0.101 | 6.33 | 81.25 | 18 | 254 | 200 | BABA | 0.000000 | 3.83652760404716E-06 |
| LAT | [Ant_aus_2C] | [Ant_lat_1] | [Ant_meo_5C] | [Sai_sub_1] | -0.605 | 0.105 | 5.75 | 77.25 | 19 | 252 | 200 | BABA | 0.000000 | 0.000139451813529 |
| LAT | [Ant_gra_bra_1] | [Ant_lat_3] | [Ant_meo_5C] | [Sai_nut_1] | -0.421 | 0.074 | 5.67 | 174.38 | 71.12 | 644 | 200 | BABA | 0.000000 | 0.000223049725591 |
| LAT | [Ant_gra_4] | [Ant_lat_4C] | [Ant_meo_5C] | [Mis_mic_1] | -0.448 | 0.08 | 5.59 | 162 | 61.75 | 553 | 200 | BABA | 0.000000 | 0.000354546492771 |
| LAT | [Ant_aus_2C] | [Ant_lat_3] | [Ant_meo_5C] | [Sai_sub_1] | -0.59 | 0.107 | 5.49 | 74.75 | 19.25 | 256 | 200 | BABA | 0.000000 | 0.000627097025148 |
| LAT | [Ant_boi_2C] | [Ant_lat_3] | [Ant_meo_5C] | [Sai_nut_1] | -0.387 | 0.071 | 5.48 | 187.12 | 82.62 | 708 | 200 | BABA | 0.000000 | 0.00066355083668 |
| LAT | [Ant_onu_3C] | [Ant_lat_4C] | [Ant_meo_5C] | [Sai_sub_1] | -0.564 | 0.106 | 5.31 | 96 | 26.75 | 298 | 200 | BABA | 0.000000 | 0.001708509286881 |
| LAT | [Ant_onu_3C] | [Ant_lat_4C] | [Ant_meo_5C] | [Pse_riv_1] | -0.575 | 0.109 | 5.26 | 89 | 24 | 305 | 200 | BABA | 0.000000 | 0.002244527212631 |
| LAT | [Ant_boi_2C] | [Ant_lat_4C] | [Ant_meo_5C] | [Sai_nut_1] | -0.394 | 0.075 | 5.24 | 184.75 | 80.25 | 712 | 200 | BABA | 0.000000 | 0.002501783384842 |
| LAT | [Ant_gra_4] | [Ant_lat_3] | [Ant_meo_5C] | [Mis_mic_1] | -0.388 | 0.074 | 5.23 | 147.25 | 65 | 547 | 200 | BABA | 0.000000 | 0.00264045788862 |
| LAT | [Ant_boi_2C] | [Ant_lat_4C] | [Ant_meo_5C] | [Mis_mic_1] | -0.359 | 0.07 | 5.15 | 201.75 | 95.25 | 657 | 200 | BABA | 0.000000 | 0.004055513681205 |
| LAT | [Ant_lin_2C] | [Ant_lat_3] | [Ant_meo_5C] | [Sai_nut_1] | -0.415 | 0.081 | 5.11 | 177.88 | 73.62 | 690 | 200 | BABA | 0.000000 | 0.005011824642084 |
| LAT | [Ant_cir_2C] | [Ant_lat_4C] | [Ant_meo_5C] | [Pse_riv_1] | -0.385 | 0.076 | 5.06 | 168.5 | 74.75 | 630 | 200 | BABA | 0.000000 | 0.006517761207559 |
| LAT | [Ant_gra_4] | [Ant_lat_4C] | [Ant_meo_5C] | [Pse_riv_1] | -0.405 | 0.081 | 5.02 | 154.88 | 65.62 | 552 | 200 | BABA | 0.000001 | 0.008028197774265 |
| LAT | [Ant_cir_2C] | [Ant_lat_3] | [Ant_meo_5C] | [Sai_nut_1] | -0.367 | 0.075 | 4.92 | 173.12 | 80.12 | 698 | 200 | BABA | 0.000001 | 0.013426469107445 |
| LAT | [Ant_gra_bra2C] | [Ant_lat_4C] | [Ant_meo_5C] | [Pse_riv_1] | -0.371 | 0.076 | 4.9 | 178.75 | 82 | 632 | 200 | BABA | 0.000001 | 0.014861390140172 |
| LAT | [Ant_aus_1C] | [Ant_lat_4C] | [Ant_meo_5C] | [Sai_nut_1] | -0.447 | 0.092 | 4.88 | 150.5 | 57.5 | 560 | 200 | BABA | 0.000001 | 0.016443305292228 |
| LAT | [Ant_gra_1C] | [Ant_lat_3] | [Ant_meo_5C] | [Sai_nut_1] | -0.416 | 0.086 | 4.85 | 149.38 | 61.62 | 520 | 200 | BABA | 0.000001 | 0.019126651614333 |
| LAT | [Ant_lin_2C] | [Ant_lat_4C] | [Ant_meo_5C] | [Sai_nut_1] | -0.399 | 0.083 | 4.83 | 173 | 74.25 | 692 | 200 | BABA | 0.000001 | 0.021144873532845 |
| LAT | [Ant_lin_2C] | [Ant_lat_4C] | [Ant_meo_5C] | [Gam_spe] | -0.34 | 0.07 | 4.83 | 202.5 | 99.75 | 783 | 200 | BABA | 0.000001 | 0.02114350820234 |
| LAT | [Ant_aus_1C] | [Ant_lat_3] | [Ant_meo_5C] | [Sai_nut_1] | -0.431 | 0.09 | 4.77 | 149.38 | 59.38 | 562 | 200 | BABA | 0.000002 | 0.028512645519758 |
| LAT | [Ant_gra_1C] | [Ant_lat_4C] | [Ant_meo_5C] | [Mis_mic_1] | -0.436 | 0.092 | 4.74 | 139.25 | 54.75 | 473 | 200 | BABA | 0.000002 | 0.033045111007739 |
| LAT | [Ant_cir_2C] | [Ant_lat_4C] | [Ant_meo_5C] | [Sai_nut_1] | -0.379 | 0.08 | 4.71 | 170.25 | 76.75 | 703 | 200 | BABA | 0.000002 | 0.03828215295385 |
| LAT | [Ant_gra_bra_1] | [Ant_lat_4C] | [Ant_meo_5C] | [Mis_mic_1] | -0.351 | 0.075 | 4.7 | 175.75 | 84.5 | 597 | 200 | BABA | 0.000003 | 0.04020015355586 |
| LAT | [Ant_gra_bra2C] | [Ant_lat_4C] | [Ant_meo_5C] | [Mis_mic_1] | -0.361 | 0.077 | 4.68 | 178.25 | 83.75 | 646 | 200 | BABA | 0.000003 | 0.044319305909413 |
| LAT | [Ant_boi_2C] | [Ant_lat_4C] | [Ant_meo_5C] | [Pse_riv_1] | -0.371 | 0.079 | 4.68 | 191.88 | 88.12 | 640 | 200 | BABA | 0.000003 | 0.044316437160245 |
| LAT | [Ant_gra_4] | [Ant_lat_1] | [Ant_meo_5C] | [Mis_mic_1] | -0.369 | 0.079 | 4.66 | 142.75 | 65.75 | 546 | 200 | BABA | 0.000003 | 0.048810080482458 |
| LAT | [Ant_boi_2C] | [Ant_lat_4C] | [Ant_mic_4C] | [Sai_nut_1] | -0.421 | 0.087 | 4.85 | 181.5 | 74 | 649 | 200 | BABA | 0.000001 | 0.019127886229077 |
| LAT | [Ant_aus_2C] | [Ant_lat_3] | [Ant_pul_1C] | [Sai_nut_1] | -0.612 | 0.103 | 5.94 | 107 | 25.75 | 295 | 200 | BABA | 0.000000 | 4.45717523573662E-05 |
| LAT | [Ant_aus_2C] | [Ant_lat_3] | [Ant_pul_1C] | [Sai_sub_1] | -0.594 | 0.101 | 5.88 | 81.5 | 20.75 | 258 | 200 | BABA | 0.000000 | 6.41369591552164E-05 |
| LAT | [Ant_aus_2C] | [Ant_lat_4C] | [Ant_pul_1C] | [Sai_sub_1] | -0.583 | 0.102 | 5.7 | 84.5 | 22.25 | 256 | 200 | BABA | 0.000000 | 0.000187175144798 |
| LAT | [Ant_aus_2C] | [Ant_lat_3] | [Ant_pul_1C] | [Gam_spe] | -0.565 | 0.11 | 5.15 | 108.75 | 30.25 | 329 | 200 | BABA | 0.000000 | 0.004056034654123 |
| LAT | [Ant_lin_2C] | [Ant_lat_3] | [Ant_pul_1C] | [Sai_nut_1] | -0.371 | 0.074 | 5.03 | 193 | 88.5 | 718 | 200 | BABA | 0.000000 | 0.007623037613144 |
| LAT | [Ant_aus_2C] | [Ant_lat_4C] | [Ant_pul_1C] | [Gam_spe] | -0.528 | 0.106 | 5 | 112.5 | 34.75 | 326 | 200 | BABA | 0.000001 | 0.008904544428855 |
| LAT | [Ant_gra_1C] | [Ant_lat_1] | [Ant_pul_1C] | [Mis_oro_1] | -0.388 | 0.081 | 4.79 | 136 | 60 | 466 | 200 | BABA | 0.000002 | 0.025819415094105 |
| LAT | [Ant_lin_2C] | [Ant_lat_1] | [Ant_pul_1C] | [Sai_nut_1] | -0.387 | 0.081 | 4.75 | 196.75 | 87 | 711 | 200 | BABA | 0.000002 | 0.031464487192106 |
| LAT | [Ant_aus_2C] | [Ant_lat_3] | [Ant_pul_3] | [Sai_nut_1] | -0.602 | 0.109 | 5.54 | 108.75 | 27 | 302 | 200 | BABA | 0.000000 | 0.000472127993358 |
| LAT | [Ant_tor_7] | [Ant_lat_3] | [Ant_pul_3] | [Sai_nut_1] | -0.388 | 0.073 | 5.34 | 195 | 86 | 792 | 200 | BABA | 0.000000 | 0.001448944259111 |
| LAT | [Ant_lin_2C] | [Ant_lat_3] | [Ant_pul_3] | [Sai_nut_1] | -0.381 | 0.074 | 5.13 | 214.25 | 96 | 764 | 200 | BABA | 0.000000 | 0.00450925752563 |
| LAT | [Ant_aus_2C] | [Ant_lat_4C] | [Ant_pul_3] | [Gam_spe] | -0.526 | 0.105 | 5.02 | 111.75 | 34.75 | 334 | 200 | BABA | 0.000001 | 0.008029231203855 |
| LAT | [Ant_aus_2C] | [Ant_lat_3] | [Ant_pul_3] | [Sai_sub_1] | -0.562 | 0.115 | 4.88 | 74 | 20.75 | 267 | 200 | BABA | 0.000001 | 0.016444366150634 |
| LAT | [Ant_gra_4] | [Ant_lat_4C] | [Ant_pul_3] | [Mis_mic_1] | -0.359 | 0.075 | 4.77 | 161.75 | 76.25 | 602 | 200 | BABA | 0.000002 | 0.028505276482989 |
| LAT | [Ant_aus_2C] | [Ant_lat_4C] | [Ant_pul_3] | [Sai_nut_1] | -0.561 | 0.119 | 4.73 | 102.25 | 28.75 | 299 | 200 | BABA | 0.000002 | 0.034713012012057 |
| LAT | [Ant_gra_bra_1] | [Ant_lat_3] | [Ant_rot_1C] | [Sai_sub_1] | -0.554 | 0.088 | 6.28 | 116 | 33.25 | 382 | 200 | BABA | 0.000000 | 5.29731361618702E-06 |
| LAT | [Ant_gra_bra_1] | [Ant_lat_1] | [Ant_rot_1C] | [Sai_sub_1] | -0.553 | 0.098 | 5.61 | 127.5 | 36.75 | 379 | 200 | BABA | 0.000000 | 0.00031599370748 |
| LAT | [Ant_onu_3C] | [Ant_lat_1] | [Ant_rot_1C] | [Pse_riv_1] | -0.613 | 0.111 | 5.54 | 102.25 | 24.5 | 243 | 200 | BABA | 0.000000 | 0.000472097746193 |
| LAT | [Ant_onu_3C] | [Ant_lat_1] | [Ant_rot_1C] | [Sai_sub_1] | -0.615 | 0.112 | 5.51 | 99.75 | 23.75 | 233 | 200 | BABA | 0.000000 | 0.000559924103478 |
| LAT | [Ant_gra_bra_1] | [Ant_lat_4C] | [Ant_rot_1C] | [Sai_sub_1] | -0.524 | 0.097 | 5.39 | 117 | 36.5 | 381 | 200 | BABA | 0.000000 | 0.001098717119925 |
| LAT | [Ant_cir_2C] | [Ant_lat_3] | [Ant_rot_1C] | [Sai_sub_1] | -0.515 | 0.097 | 5.31 | 128.38 | 41.12 | 420 | 200 | BABA | 0.000000 | 0.001708399661646 |
| LAT | [Ant_lin_2C] | [Ant_lat_3] | [Ant_rot_1C] | [Sai_sub_1] | -0.507 | 0.098 | 5.2 | 117.38 | 38.38 | 417 | 200 | BABA | 0.000000 | 0.003103918797725 |
| LAT | [Ant_cir_2C] | [Ant_lat_1] | [Ant_rot_1C] | [Sai_sub_1] | -0.517 | 0.1 | 5.19 | 135 | 43 | 417 | 200 | BABA | 0.000000 | 0.003275120011375 |
| LAT | [Ant_gra_1C] | [Ant_lat_3] | [Ant_rot_1C] | [Sai_sub_1] | -0.524 | 0.103 | 5.11 | 108.75 | 34 | 341 | 200 | BABA | 0.000000 | 0.005012146800896 |
| LAT | [Ant_onu_3C] | [Ant_lat_1] | [Ant_rot_1C] | [Mis_oro_1] | -0.616 | 0.121 | 5.09 | 93.75 | 22.25 | 231 | 200 | BABA | 0.000000 | 0.005568603777499 |
| LAT | [Ant_gra_1C] | [Ant_lat_4C] | [Ant_rot_1C] | [Mis_oro_1] | -0.49 | 0.098 | 5.02 | 111 | 38 | 342 | 200 | BABA | 0.000001 | 0.00802871448906 |
| LAT | [Ant_onu_3C] | [Ant_lat_3] | [Ant_rot_1C] | [Mis_oro_1] | -0.592 | 0.119 | 4.96 | 84.38 | 21.62 | 231 | 200 | BABA | 0.000001 | 0.010943361206582 |
| LAT | [Ant_gra_1C] | [Ant_lat_3] | [Ant_rot_1C] | [Mis_oro_1] | -0.515 | 0.104 | 4.96 | 123.25 | 39.5 | 338 | 200 | BABA | 0.000001 | 0.010942656274785 |
| LAT | [Ant_cir_2C] | [Ant_lat_4C] | [Ant_rot_1C] | [Sai_sub_1] | -0.485 | 0.098 | 4.95 | 127.38 | 44.12 | 421 | 200 | BABA | 0.000001 | 0.011519416612814 |
| LAT | [Ant_his_1C] | [Ant_lat_1] | [Ant_rot_1C] | [Sai_sub_1] | -0.486 | 0.099 | 4.92 | 130.25 | 45 | 412 | 200 | BABA | 0.000001 | 0.013427334549569 |
| LAT | [Ant_onu_3C] | [Ant_lat_4C] | [Ant_rot_1C] | [Mis_oro_1] | -0.545 | 0.111 | 4.89 | 82.25 | 24.25 | 236 | 200 | BABA | 0.000001 | 0.015633609978191 |
| LAT | [Ant_cir_2C] | [Ant_lat_4C] | [Ant_rot_1C] | [How_ova_1] | -0.466 | 0.095 | 4.88 | 112.5 | 41 | 440 | 200 | BABA | 0.000001 | 0.01644542700904 |
| LAT | [Ant_cir_2C] | [Ant_lat_3] | [Ant_rot_1C] | [Mis_oro_1] | -0.411 | 0.084 | 4.88 | 140.75 | 58.75 | 432 | 200 | BABA | 0.000001 | 0.01644012271701 |
| LAT | [Ant_cir_2C] | [Ant_lat_1] | [Ant_rot_1C] | [Pse_riv_1] | -0.454 | 0.094 | 4.86 | 151.25 | 56.75 | 435 | 200 | BABA | 0.000001 | 0.018187748553292 |
| LAT | [Ant_gra_1C] | [Ant_lat_4C] | [Ant_rot_1C] | [Mis_mic_1] | -0.464 | 0.096 | 4.84 | 109.25 | 40 | 352 | 200 | BABA | 0.000001 | 0.020110782624559 |
| LAT | [Ant_cir_2C] | [Ant_lat_3] | [Ant_rot_1C] | [Pse_riv_1] | -0.443 | 0.092 | 4.82 | 148.25 | 57.25 | 438 | 200 | BABA | 0.000001 | 0.022228554999932 |
| LAT | [Ant_lin_2C] | [Ant_lat_1] | [Ant_rot_1C] | [Sai_sub_1] | -0.504 | 0.105 | 4.81 | 119.75 | 39.5 | 413 | 200 | BABA | 0.000002 | 0.023368537703247 |
| LAT | [Ant_cir_2C] | [Ant_lat_4C] | [Ant_rot_1C] | [Sai_nut_1] | -0.425 | 0.09 | 4.72 | 147.62 | 59.62 | 490 | 200 | BABA | 0.000002 | 0.036456864961821 |
| LAT | [Ant_onu_3C] | [Ant_lat_4C] | [Ant_rot_1C] | [Mis_mic_1] | -0.525 | 0.112 | 4.67 | 88.25 | 27.5 | 245 | 200 | BABA | 0.000003 | 0.046502227166814 |
| LAT | [Ant_onu_3C] | [Ant_lat_3] | [Ant_rot_1C] | [Sai_sub_1] | -0.563 | 0.121 | 4.66 | 80.88 | 22.62 | 233 | 200 | BABA | 0.000003 | 0.048816404670134 |
| LAT | [Ant_gra_4] | [Ant_lat_3] | [Ant_val_1C] | [Sai_sub_1] | -0.419 | 0.086 | 4.89 | 128.25 | 52.5 | 487 | 200 | BABA | 0.000001 | 0.015634618337967 |
| SIC | [Ant_gra_4] | [Ant_sic_1C] | [Ant_bra_1C] | [Mis_mic_1] | -0.38 | 0.074 | 5.11 | 162 | 72.75 | 562 | 200 | BABA | 0.000000 | 0.005034697917754 |
| SIC | [Ant_gra_4] | [Ant_die_1C] | [Ant_bra_1C] | [Mis_mic_1] | -0.337 | 0.071 | 4.75 | 161 | 79.75 | 578 | 200 | BABA | 0.000002 | 0.031726894668688 |
| SIC | [Ant_aus_1C] | [Ant_sic_1C] | [Ant_gro_1C] | [Pse_riv_1] | -0.594 | 0.108 | 5.5 | 55 | 14 | 185 | 200 | BABA | 0.000000 | 0.000593955534808 |
| SIC | [Ant_aus_1C] | [Ant_die_1C] | [Ant_gro_1C] | [Pse_riv_1] | -0.58 | 0.11 | 5.28 | 56.5 | 15 | 187 | 200 | BABA | 0.000000 | 0.002019531697962 |
| SIC | [Ant_aus_1C] | [Ant_sic_2C] | [Ant_gro_1C] | [Pse_riv_1] | -0.583 | 0.116 | 5.03 | 57 | 15 | 187 | 200 | BABA | 0.000000 | 0.007663747439543 |
| SIC | [Ant_con_1C] | [Ant_die_1C] | [Ant_gro_1C] | [Mis_mic_1] | -0.527 | 0.107 | 4.95 | 69.5 | 21.5 | 222 | 200 | BABA | 0.000001 | 0.011592145824775 |
| SIC | [Ant_con_1C] | [Ant_sic_1C] | [Ant_gro_1C] | [Mis_mic_1] | -0.525 | 0.11 | 4.77 | 67.5 | 21 | 218 | 200 | BABA | 0.000002 | 0.028739243400415 |
| SIC | [Ant_cir_2C] | [Ant_sic_1C] | [Ant_gro_2C] | [Pse_riv_1] | -0.377 | 0.075 | 5 | 177.25 | 80.25 | 537 | 200 | BABA | 0.000001 | 0.008956141711794 |
| SIC | [Ant_gra_4] | [Ant_die_1C] | [Ant_lop_1C] | [Pse_riv_1] | -0.493 | 0.072 | 6.86 | 126 | 42.75 | 428 | 200 | BABA | 0.000000 | 1.07835618144103E-07 |
| SIC | [Ant_gra_4] | [Ant_sic_2C] | [Ant_lop_1C] | [Pse_riv_1] | -0.499 | 0.073 | 6.85 | 127.75 | 42.75 | 430 | 200 | BABA | 0.000000 | 1.15641697062165E-07 |
| SIC | [Ant_gra_4] | [Ant_sic_1C] | [Ant_lop_1C] | [Pse_riv_1] | -0.503 | 0.078 | 6.45 | 124.75 | 41.25 | 424 | 200 | BABA | 0.000000 | 1.75134967248221E-06 |
| SIC | [Ant_gra_1C] | [Ant_die_1C] | [Ant_lop_1C] | [Mis_mic_1] | -0.484 | 0.079 | 6.14 | 104.25 | 36.25 | 368 | 200 | BABA | 0.000000 | 1.29187378267444E-05 |
| SIC | [Ant_gra_1C] | [Ant_sic_1C] | [Ant_lop_1C] | [Mis_mic_1] | -0.488 | 0.083 | 5.88 | 104 | 35.75 | 359 | 200 | BABA | 0.000000 | 6.42067044571827E-05 |
| SIC | [Ant_gra_1C] | [Ant_sic_2C] | [Ant_lop_1C] | [Mis_mic_1] | -0.478 | 0.084 | 5.68 | 104 | 36.75 | 366 | 200 | BABA | 0.000000 | 0.000210743393382 |
| SIC | [Ant_gra_1C] | [Ant_die_1C] | [Ant_lop_1C] | [Pse_riv_1] | -0.489 | 0.087 | 5.65 | 103.5 | 35.5 | 367 | 200 | BABA | 0.000000 | 0.000251020641007 |
| SIC | [Ant_gra_1C] | [Ant_sic_1C] | [Ant_lop_1C] | [Pse_riv_1] | -0.481 | 0.086 | 5.61 | 101.25 | 35.5 | 363 | 200 | BABA | 0.000000 | 0.000316479291356 |
| SIC | [Ant_gra_1C] | [Ant_sic_2C] | [Ant_lop_1C] | [Mis_oro_1] | -0.444 | 0.081 | 5.45 | 89 | 34.25 | 347 | 200 | BABA | 0.000000 | 0.000787683246863 |
| SIC | [Ant_gra_1C] | [Ant_sic_2C] | [Ant_lop_1C] | [Pse_riv_1] | -0.486 | 0.09 | 5.4 | 102.75 | 35.5 | 367 | 200 | BABA | 0.000000 | 0.001041997065036 |
| SIC | [Ant_gra_4] | [Ant_die_1C] | [Ant_lop_1C] | [Mis_mic_1] | -0.413 | 0.081 | 5.1 | 116.25 | 48.25 | 426 | 200 | BABA | 0.000000 | 0.005307764954299 |
| SIC | [Ant_gra_1C] | [Ant_die_1C] | [Ant_lop_1C] | [How_ova_1] | -0.458 | 0.091 | 5.02 | 85.5 | 31.75 | 391 | 200 | BABA | 0.000001 | 0.008072635246659 |
| SIC | [Ant_gra_1C] | [Ant_sic_2C] | [Ant_lop_1C] | [Gam_spe] | -0.395 | 0.08 | 4.97 | 96.25 | 41.75 | 456 | 200 | BABA | 0.000001 | 0.010458712767484 |
| SIC | [Ant_gra_4] | [Ant_sic_2C] | [Ant_lop_1C] | [Mis_mic_1] | -0.42 | 0.085 | 4.91 | 118 | 48.25 | 429 | 200 | BABA | 0.000001 | 0.014217935706257 |
| SIC | [Ant_gra_1C] | [Ant_die_1C] | [Ant_lop_1C] | [Mis_oro_1] | -0.453 | 0.094 | 4.85 | 89.75 | 33.75 | 349 | 200 | BABA | 0.000001 | 0.019268632309895 |
| SIC | [Ant_gra_4] | [Ant_sic_1C] | [Ant_lop_1C] | [Mis_mic_1] | -0.425 | 0.088 | 4.83 | 116 | 46.75 | 420 | 200 | BABA | 0.000001 | 0.021307347862955 |
| SIC | [Ant_gra_1C] | [Ant_sic_1C] | [Ant_lop_1C] | [How_ova_1] | -0.442 | 0.093 | 4.76 | 81.5 | 31.5 | 384 | 200 | BABA | 0.000002 | 0.030198565706629 |
| SIC | [Ant_gra_1C] | [Ant_die_1C] | [Ant_lop_1C] | [Sai_nut_1] | -0.416 | 0.088 | 4.7 | 97.5 | 40.25 | 415 | 200 | BABA | 0.000003 | 0.040561778028049 |
| SIC | [Ant_gra_4] | [Ant_sic_1C] | [Ant_lop_2] | [Pse_riv_1] | -0.432 | 0.068 | 6.35 | 169 | 67 | 553 | 200 | BABA | 0.000000 | 3.37118582303701E-06 |
| SIC | [Ant_gra_4] | [Ant_sic_2C] | [Ant_lop_2] | [Pse_riv_1] | -0.402 | 0.064 | 6.24 | 166.5 | 71 | 561 | 200 | BABA | 0.000000 | 6.85060991029253E-06 |
| SIC | [Ant_boi_2C] | [Ant_sic_1C] | [Ant_lop_2] | [Pse_riv_1] | -0.42 | 0.069 | 6.12 | 217.5 | 88.75 | 649 | 200 | BABA | 0.000000 | 1.46482864604878E-05 |
| SIC | [Ant_cir_2C] | [Ant_sic_1C] | [Ant_lop_2] | [Pse_riv_1] | -0.432 | 0.071 | 6.07 | 212.5 | 84.25 | 645 | 200 | BABA | 0.000000 | 2.00217914359331E-05 |
| SIC | [Ant_cir_2C] | [Ant_die_1C] | [Ant_lop_2] | [Pse_riv_1] | -0.402 | 0.066 | 6.06 | 211.75 | 90.25 | 666 | 200 | BABA | 0.000000 | 2.13057447853199E-05 |
| SIC | [Ant_boi_2C] | [Ant_die_1C] | [Ant_lop_2] | [Pse_riv_1] | -0.41 | 0.069 | 5.94 | 216.5 | 90.5 | 667 | 200 | BABA | 0.000000 | 4.46088052273397E-05 |
| SIC | [Ant_cir_2C] | [Ant_sic_2C] | [Ant_lop_2] | [Pse_riv_1] | -0.4 | 0.069 | 5.82 | 211 | 90.5 | 658 | 200 | BABA | 0.000000 | 9.2090652150947E-05 |
| SIC | [Ant_boi_2C] | [Ant_sic_2C] | [Ant_lop_2] | [Pse_riv_1] | -0.4 | 0.069 | 5.82 | 217.5 | 93.25 | 662 | 200 | BABA | 0.000000 | 9.20847673882049E-05 |
| SIC | [Ant_gra_4] | [Ant_die_1C] | [Ant_lop_2] | [Pse_riv_1] | -0.416 | 0.073 | 5.73 | 166.5 | 68.75 | 564 | 200 | BABA | 0.000000 | 0.0001571438138 |
| SIC | [Ant_gra_4] | [Ant_sic_1C] | [Ant_lop_2] | [Mis_mic_1] | -0.417 | 0.074 | 5.64 | 167 | 68.75 | 554 | 200 | BABA | 0.000000 | 0.000266026477046 |
| SIC | [Ant_gra_bra_1] | [Ant_die_1C] | [Ant_lop_2] | [Pse_riv_1] | -0.353 | 0.063 | 5.62 | 180 | 86 | 609 | 200 | BABA | 0.000000 | 0.000298714753042 |
| SIC | [Ant_gra_bra_1] | [Ant_sic_1C] | [Ant_lop_2] | [Pse_riv_1] | -0.376 | 0.067 | 5.57 | 181.25 | 82.25 | 588 | 200 | BABA | 0.000000 | 0.00039843779454 |
| SIC | [Ant_gra_4] | [Ant_die_1C] | [Ant_lop_2] | [Mis_mic_1] | -0.386 | 0.07 | 5.54 | 169.62 | 75.12 | 570 | 200 | BABA | 0.000000 | 0.000473065655463 |
| SIC | [Ant_lin_4] | [Ant_sic_2C] | [Ant_lop_2] | [Gam_spe] | -0.337 | 0.062 | 5.42 | 240.12 | 119.12 | 829 | 200 | BABA | 0.000000 | 0.000931950116879 |
| SIC | [Ant_cir_2C] | [Ant_sic_1C] | [Ant_lop_2] | [Sai_nut_1] | -0.374 | 0.07 | 5.37 | 183.62 | 83.62 | 732 | 200 | BABA | 0.000000 | 0.001231047399883 |
| SIC | [Ant_boi_2C] | [Ant_sic_1C] | [Ant_lop_2] | [Mis_mic_1] | -0.37 | 0.07 | 5.29 | 214.5 | 98.75 | 661 | 200 | BABA | 0.000000 | 0.001912293971374 |
| SIC | [Ant_lin_4] | [Ant_sic_2C] | [Ant_lop_2] | [Pse_riv_1] | -0.358 | 0.069 | 5.2 | 204 | 96.5 | 644 | 200 | BABA | 0.000000 | 0.003115278243726 |
| SIC | [Ant_lin_2C] | [Ant_sic_1C] | [Ant_lop_2] | [Pse_riv_1] | -0.343 | 0.067 | 5.16 | 206.5 | 101 | 641 | 200 | BABA | 0.000000 | 0.003860072987696 |
| SIC | [Ant_gra_bra_1] | [Ant_sic_2C] | [Ant_lop_2] | [Pse_riv_1] | -0.351 | 0.068 | 5.15 | 179 | 86 | 602 | 200 | BABA | 0.000000 | 0.004071403355208 |
| SIC | [Ant_maj_3] | [Ant_sic_1C] | [Ant_lop_2] | [Pse_riv_1] | -0.356 | 0.07 | 5.13 | 186 | 88.25 | 569 | 200 | BABA | 0.000000 | 0.004528380509418 |
| SIC | [Ant_cir_2C] | [Ant_die_1C] | [Ant_lop_2] | [Sai_nut_1] | -0.363 | 0.072 | 5.05 | 187.62 | 87.62 | 758 | 200 | BABA | 0.000000 | 0.006903724068488 |
| SIC | [Ant_lin_4] | [Ant_die_1C] | [Ant_lop_2] | [Pse_riv_1] | -0.361 | 0.072 | 5.03 | 202.5 | 95 | 649 | 200 | BABA | 0.000000 | 0.007663256959707 |
| SIC | [Ant_lin_4] | [Ant_sic_1C] | [Ant_lop_2] | [Pse_riv_1] | -0.365 | 0.074 | 4.95 | 200.5 | 93.25 | 633 | 200 | BABA | 0.000001 | 0.011591403689959 |
| SIC | [Ant_lin_4] | [Ant_sic_2C] | [Ant_lop_2] | [Mis_mic_1] | -0.342 | 0.069 | 4.94 | 214.75 | 105.25 | 669 | 200 | BABA | 0.000001 | 0.012200401891779 |
| SIC | [Ant_lin_2C] | [Ant_sic_1C] | [Ant_lop_2] | [Gam_spe] | -0.339 | 0.069 | 4.93 | 221.88 | 109.62 | 818 | 200 | BABA | 0.000001 | 0.012840976954614 |
| SIC | [Ant_cir_2C] | [Ant_sic_1C] | [Ant_lop_2] | [Mis_mic_1] | -0.35 | 0.071 | 4.93 | 204 | 98.25 | 662 | 200 | BABA | 0.000001 | 0.012840154658446 |
| SIC | [Ant_gra_bra2C] | [Ant_sic_1C] | [Ant_lop_2] | [Pse_riv_1] | -0.321 | 0.065 | 4.93 | 195.25 | 100.25 | 640 | 200 | BABA | 0.000001 | 0.012839332362278 |
| SIC | [Ant_gra_bra2C] | [Ant_sic_2C] | [Ant_lop_2] | [Pse_riv_1] | -0.304 | 0.062 | 4.92 | 196.25 | 104.75 | 654 | 200 | BABA | 0.000001 | 0.013512147877694 |
| SIC | [Ant_lin_2C] | [Ant_sic_2C] | [Ant_lop_2] | [Gam_spe] | -0.329 | 0.067 | 4.91 | 226.12 | 114.12 | 839 | 200 | BABA | 0.000001 | 0.014218846470187 |
| SIC | [Ant_gra_1C] | [Ant_sic_1C] | [Ant_lop_2] | [Mis_mic_1] | -0.378 | 0.077 | 4.89 | 145 | 65.5 | 462 | 200 | BABA | 0.000001 | 0.015739487754746 |
| SIC | [Ant_lin_4] | [Ant_sic_1C] | [Ant_lop_2] | [Mis_mic_1] | -0.338 | 0.07 | 4.83 | 209.75 | 103.75 | 650 | 200 | BABA | 0.000001 | 0.02130598253245 |
| SIC | [Ant_maj_1C] | [Ant_sic_1C] | [Ant_lop_2] | [Pse_riv_1] | -0.344 | 0.071 | 4.83 | 172.25 | 84 | 550 | 200 | BABA | 0.000001 | 0.021304617201945 |
| SIC | [Ant_cir_2C] | [Ant_sic_2C] | [Ant_lop_2] | [Sai_nut_1] | -0.345 | 0.072 | 4.8 | 185.5 | 90.25 | 752 | 200 | BABA | 0.000002 | 0.024756598310551 |
| SIC | [Ant_lin_4] | [Ant_die_1C] | [Ant_lop_2] | [Gam_spe] | -0.333 | 0.07 | 4.79 | 237 | 118.5 | 837 | 200 | BABA | 0.000002 | 0.026021220483059 |
| SIC | [Ant_lin_2C] | [Ant_die_1C] | [Ant_lop_2] | [Gam_spe] | -0.321 | 0.067 | 4.77 | 228.75 | 117.5 | 846 | 200 | BABA | 0.000002 | 0.028741085659607 |
| SIC | [Ant_lin_4] | [Ant_sic_2C] | [Ant_lop_2] | [Neo_fil_1] | -0.328 | 0.069 | 4.74 | 199.75 | 101 | 746 | 200 | BABA | 0.000002 | 0.033331493421077 |
| SIC | [Ant_cir_2C] | [Ant_sic_1C] | [Ant_lop_2] | [Neo_fil_1] | -0.36 | 0.076 | 4.72 | 185.75 | 87.5 | 738 | 200 | BABA | 0.000002 | 0.036779972123147 |
| SIC | [Ant_lin_4] | [Ant_sic_1C] | [Ant_lop_2] | [Gam_spe] | -0.324 | 0.069 | 4.71 | 229.12 | 116.88 | 807 | 200 | BABA | 0.000002 | 0.038628956461909 |
| SIC | [Ant_boi_2C] | [Ant_die_1C] | [Ant_lop_2] | [Mis_mic_1] | -0.343 | 0.073 | 4.71 | 216.12 | 105.62 | 685 | 200 | BABA | 0.000002 | 0.038626479293994 |
| SIC | [Ant_aus_2C] | [Ant_sic_1C] | [Ant_lop_2] | [Pse_riv_1] | -0.478 | 0.102 | 4.71 | 80.75 | 28.5 | 264 | 200 | BABA | 0.000002 | 0.03862400212608 |
| SIC | [Ant_lin_4] | [Ant_die_1C] | [Ant_lop_2] | [Neo_fil_1] | -0.327 | 0.07 | 4.7 | 197.62 | 100.12 | 752 | 200 | BABA | 0.000003 | 0.040559176413141 |
| SIC | [Ant_lin_2C] | [Ant_sic_2C] | [Ant_lop_2] | [Pse_riv_1] | -0.323 | 0.069 | 4.68 | 205 | 105 | 654 | 200 | BABA | 0.000003 | 0.044720930793051 |
| SIC | [Ant_gra_4] | [Ant_sic_1C] | [Ant_meo_5C] | [Mis_mic_1] | -0.393 | 0.081 | 4.89 | 152.75 | 66.5 | 523 | 200 | BABA | 0.000001 | 0.015740496114522 |
| SIC | [Ant_gra_1C] | [Ant_sic_1C] | [Ant_meo_5C] | [Mis_oro_1] | -0.387 | 0.08 | 4.87 | 120 | 53 | 421 | 200 | BABA | 0.000001 | 0.017418254648037 |
| SIC | [Ant_gra_4] | [Ant_sic_1C] | [Ant_meo_5C] | [Pse_riv_1] | -0.369 | 0.079 | 4.67 | 144.75 | 66.75 | 528 | 200 | BABA | 0.000003 | 0.046951014772738 |
| SIC | [Ant_gra_4] | [Ant_die_1C] | [Ant_pul_3] | [Mis_mic_1] | -0.386 | 0.078 | 4.94 | 154.5 | 68.5 | 578 | 200 | BABA | 0.000001 | 0.012201183117487 |
| SIC | [Ant_gra_4] | [Ant_sic_1C] | [Ant_pul_3] | [Mis_mic_1] | -0.383 | 0.082 | 4.66 | 152.5 | 68 | 561 | 200 | BABA | 0.000003 | 0.049287556651987 |
| SIC | [Ant_cir_2C] | [Ant_sic_1C] | [Ant_rot_1C] | [Pse_riv_1] | -0.421 | 0.088 | 4.76 | 137.5 | 56 | 429 | 200 | BABA | 0.000002 | 0.030196629777037 |

**Data S1.** Locations of *Antirrhinum* photographs included in Fig. 2 of the main text. The photographs are arranged by subsections: subsect. *Antirrhinum* (A-L); subsect *Kickxella* (M-X); and subsect. *Streptosepalum* (Y-Z). Photographs: A, *A. australe* (Jaen, Santiago de la Espada; B, *A. barrelieri* (Zaragoza, Nuévalos); C, *A. cirrhigerum* (Cádiz, Caños de Meca); D, *A. controversum* (Almería, Mojácar); E, *A. graniticum* subsp. *graniticum* (Madrid, Colmenar Viejo); F, *A. latifolium* (Isère, Alps, France); G, *A. linkianum* (Portugal); H*, A. majus* subsp. *majus* (Huesca, Biescas); I, *A. majus* subsp. *striatum* (Lérida, Planes); J, *A. onubensis* (Huelva, Aracena); K, *A. siculum* (Malta, Ghajn Tuffieh); L. *A. tortuosum* (Morocco, Taza); M, *A. charidemi* (Almería, Cabo de Gata); N, *A. grosii* (Ávila, Gredos, El Morezón); Ñ, *A. hispanicum* (Granada, Sierra Nevada, Fuente Alta); O, *A. lopesianum* (Salamanca); P, *A. microphyllum* (Cuenca, Buendía); Q, *A. molle* (Huesca, Gabasa); R, *A. mollissimum* (Almería, Barranco del Caballar); S, *A. pertegasii* (Castellón, Barranco del Infierno); T, *A. pulverulentum* (Guadalajara, Durón); U, *A. rupestre* (Almería, Abrucena); V, *A. sempervirens* (Huesca, Panticosa); W, *A. subbaeticum* (Albacete); X, *A. valentinum* (Valencia, Pla de Corrals); Y, *A. braun-blanquetii* (Cantabria, Bielva); and Z, *A. meonanthum* (Madrid, Rozas de Puerto Real). All photographs were taken in Spain by Pablo Vargas, except for F (van der Straten, Saxifraga Foundation), G (Luis Nunes; Wikipedia); K (Denis Barthel; Wikipedia), and W (José Quiles; www.florasilvestre.es).
